# pdCSM-GPCR: predicting potent GPCR ligands with graph-based signatures

**DOI:** 10.1093/bioadv/vbab031

**Published:** 2021-11-10

**Authors:** João Paulo L Velloso, David B Ascher, Douglas E V Pires

**Affiliations:** 1 Fundação Oswaldo Cruz, Instituto René Rachou, Belo Horizonte 30190-009, Brazil; 2 Structural Biology and Bioinformatics, Department of Biochemistry, University of Melbourne, Melbourne 3052, Australia; 3 Systems and Computational Biology, Bio21 Institute, University of Melbourne, Melbourne 3052, Australia; 4 Computational Biology and Clinical Informatics, Baker Heart and Diabetes Institute, Melbourne 3004, Australia; 5 Universidade Federal de Minas Gerais, Belo Horizonte 31270-901, Brazil; 6 Baker Department of Cardiometabolic Health, Melbourne Medical School, University of Melbourne, Melbourne 3052, Australia; 7 Department of Biochemistry, University of Cambridge, Cambridge CB2 1GA, UK; 8 School of Computing and Information Systems, University of Melbourne, Melbourne 3053, Australia

## Abstract

**Motivation:**

G protein-coupled receptors (GPCRs) can selectively bind to many types of ligands, ranging from light-sensitive compounds, ions, hormones, pheromones and neurotransmitters, modulating cell physiology. Considering their role in many essential cellular processes, they are one of the most targeted protein families, with over a third of all approved drugs modulating GPCR signalling. Despite this, the large diversity of receptors and their multipass transmembrane architectures make the identification and development of novel specific, and safe GPCR ligands a challenge. While computational approaches have the potential to assist GPCR drug development, they have presented limited performance and generalization capabilities. Here, we explored the use of graph-based signatures to develop pdCSM-GPCR, a method capable of rapidly and accurately screening potential GPCR ligands.

**Results:**

Bioactivity data (IC50, EC50, *K*i and *K*d) for individual GPCRs were curated. After curation, we used the data for developing predictive models for 36 major GPCR targets, across 4 classes (A, B, C and F). Our models compose the most comprehensive computational resource for GPCR bioactivity prediction to date. Across stratified 10-fold cross-validation and blind tests, our approach achieved Pearson’s correlations of up to 0.89, significantly outperforming previous methods. Interpreting our results, we identified common important features of potent GPCRs ligands, which tend to have bicyclic rings, leading to higher levels of aromaticity. We believe pdCSM-GPCR will be an invaluable tool to assist screening efforts, enriching compound libraries and ranking candidates for further experimental validation.

**Availability and implementation:**

pdCSM-GPCR predictive models and datasets used have been made available via a freely accessible and easy-to-use web server at http://biosig.unimelb.edu.au/pdcsm_gpcr/.

**Supplementary information:**

Supplementary data are available at *Bioinformatics Advances* online.

## 1 Introduction

G protein-coupled receptors (GPCRs) are members of the largest and most diverse group of membrane receptors in eukaryotes. GPCRs account for 4% of human genes (Kooistra *et al.*, 2021) and are responsible for approximately two-thirds of hormones and neurotransmitters (Foster *et al.*, 2019). Orchestrating one of the major eukaryotic signalling pathways, GPCRs and their associated signalling modules are conserved from excavates to animals (de Mendoza *et al.*, 2014).

Considering their key role in many fundamental physiological functions, it is unsurprising that they are correlated with many human pathological processes, including Parkinson’s and Alzheimer’s disease, anxiety, obesity, diabetes and neurological disorders. This has also led to them being of enormous interest as drug targets (Zhang and Xie, 2012). There are ∼700 approved drugs targeting GPCRs, which corresponds to 34% of all approved drugs by the United States Food and Drug Administration (Hauser *et al.*, 2017).

GPCRs can selectively bind to many types of ligands, ranging from light-sensitive compounds, ions, hormones, pheromones and neurotransmitters and broadcast signals from the outside of the cell to its intracellular environment. This information transmission is achieved through regulation of coupling and decoupling of their effector proteins, the heterotrimeric G proteins (composed of three different subunits) or arrestins. The message is amplified and modulates cell physiology.

GPCRs also exhibit great functional and structural plasticity, which is essential for their physiological functions. This conformational complexity and variability, in addition to being membrane bound, pose a number of challenges to their structure elucidation. Many modifications for structural studies are usually needed (Milić and Veprintsev, 2015) and in the last years, new approaches to facilitate GPCR structure elucidation were assessed including recombinant overexpression and purification strategies (Errey and Fiez-Vandal, 2020), crystallization platforms (Parker and Newstead, 2012) and detergent studies (Lee *et al.*, 2020). These experimental efforts coupled with the evolution of computational methods [molecular dynamics, integrative modelling and machine learning (Zhu *et al.*, 2021)] led to the development of high-quality models for 3D structures of GPCR deposited in dedicated repositories, such as the GPCRdb (Pándy-Szekeres *et al.*, 2018) and GPCR-EXP. Despite structural data availability, the absence of 3D structures for many GPCRs has limited the ability to employ rational structure-based drug development approaches (Heifetz *et al.*, 2015). More recently, the development of AlphaFold2 (Jumper *et al.*, 2021) has promised to contribute to further expanding the structural coverage of GPCRs and facilitate further receptor-based drug discovery. For instance, Congreve *et al.* (2012) and Langmead *et al.* (2012) demonstrated that lead identification targeting adenosine A_2__*A*_ receptor using Structure-Based Drug Discovery (SBDD) and discovered preclinical candidates for potential treatment of Parkinson’s disease using biophysical mapping and co-crystallized receptors with ligands. Langmead *et al.* (2012) carried out an *in silico* screening of 545,000 compounds, using the homology model of the receptor (based on the crystal structure of the turkey *β*1 adrenergic receptor complexed with cyanopindolol), which resulted in 20 confirmed hits *in vitro*. Christopher *et al.* (2015) performed a fragment screening of a thermostabilized mGlu5 receptor and, following this procedure, used a SBDD approach to optimize the lead and developed a high potent series of negative allosteric modulators for this metabotropic GPCR. Besides these studies, it is important to mention some reviews that focussed on identification of ligands for orphan GPCRs. Ngo *et al.* (2016) covered the methods used to establish the appropriate signalling assays to test orphan receptor activity; they also covered examples of structure-based methods for targeting orphan GPCRs. Huang *et al.* (2015) used a yeast-based screening against understudied GPR68, and SBDD and identified the benzodiazepine drug lorazepam as a non-selective GPR68 positive allosteric modulator.

In the absence of the receptor structure, alternative ligand-based techniques have been explored, including the use of quantitative structure–activity relationship models based on the knowledge of ligands known to interact with a receptor of interest (Acharya *et al.*, 2011). The availability of these datasets of small-molecule activity against GPCRs also opens the opportunity to harness other *in silico* ligand-based screening approaches, including the development of machine-learning models.

Relevant efforts have been dedicated to producing reliable predictors of GPCR ligands, most of which, however, are limited to one receptor type. Ahmed *et al.* (2021) developed a classifier for GPCRs combining molecular fingerprinting and ensemble machine-learning algorithms. Another example is the study by Seo *et al.* (2018), which also aimed to develop classification models. They used a mix of ligand information and GPCRs structural features to develop predictive models. According to them, their method reached an average area under the curve of 0.94. Both mentioned studies support the idea that mixing multiple ligand features is a good start point to characterize ligands. Other studies focussed on one type of GPCR only include Cannabinoid receptor (Hu *et al.*, 2016), Adenosine receptor (He *et al.*, 2016), Dopamine receptor (Koutsoukas *et al.*, 2017; Kuang *et al.*, 2016), Serotonin receptor (Kurczab *et al.*, 2016; Rataj *et al.*, 2018) and Olfactory receptors (Bushdid *et al.*, 2018). These studies show that a range of molecular properties and fingerprints can be used to effectively describe GPCR ligands. More recently, a few studies have attempted to produce more general workflows to predict ligands for multiple GPCRs classes (Wu *et al.*, 2018, 2019). Of note, Wu *et al.* (2018) used weighted deep learning and random forest to develop the WDL-RF method, predicting ligands for 26 types of GPCRs, covering four major classes (A, B, C and F). The WDL-RF method, even though contributed to advancing the field, has also presented limited reproducibility, considering that part of the dataset used in their training was not available. Adding to that, the robustness from their models has been put into question, because the results obtained using their web server were not consistent with the results presented in the original paper. We also consider that these studies could be extended to cover a more comprehensive set of GPCR types and classes. In 2019, they proposed an iteration of their method, SED (Wu *et al.*, 2019). It couples long extended-connectivity fingerprints with deep neural network training using a dataset of 16 types of GPCRs (covering classes A, B, C and F). The drawback of the SED method is the lack of a web server, which is essential for users with little programming expertise. Finally, both strategies applied the use of ‘controls’ on the datasets, which are artificially considered to have a very low bioactivity, without further experimental support.

Recently, Sakai *et al.* (2021) used graph convolutional neural networks for representing ligands and used this information to develop models to predict bioactivity of small molecules against 127 diverse targets, further demonstrating the effectiveness of graph-based methods for ligand discovery. We have also previously shown that using graph-based signatures to represent small-molecule chemical structures enables the accurate prediction of small-molecule pharmacokinetics, toxicity and bioactivity properties (Pires *et al.*, 2015) (see Supplementary Fig. S1 for more information about modelling small-molecule activity using graph-based signatures). Here, we therefore proposed to explore the utility of this approach to accurately identify potential GPCR ligands, by developing a computational platform dedicated to GPCR ligand design, pdCSM-GPCR. Our models are capable of quantitatively predicting ligand bioactivity for the most comprehensive set of GPCR types and classes (A, B, C and F) to date.

## 2 Methods

**Fig. 1.**
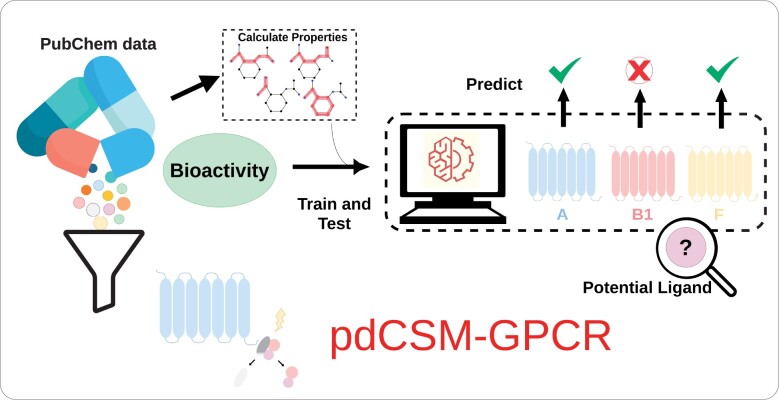
pdCSM-GPCR workflow. Initially, we collected ligand data for 36 different GPCRs from PubChem, then derived from them two types of features: compound general properties (including molecular properties, toxicophores and pharmacophores) and distance-based graph signatures. Afterwards, we used this information as the basis for the development of machine-learning models for predicting bioactivity for GPCRs

The general pdCSM-GPCR workflow is depicted in Figure 1. It is composed of three main steps including: (i) dataset acquisition, which refers to collecting experimental data from public repositories about ligands for 36 different GPCRs; (ii) feature engineering, which encompasses the generation and evaluation of features selected to model different aspects involved in binding between ligand and receptor and (iii) machine learning, which aims to train, test and validate predictive model via supervised learning, using the computed features and experimental values of bioactivity, as evidence.

### 2.1 Datasets

We initially retrieved small-molecule bioactivities for 26 different GPCRs, covering four major classes, from PubChem (Kim *et al.*, 2019), for the sake of performing a direct comparison with a previous method, WDL-RF (Wu *et al.*, 2018). The most targeted GPCR class has historically been class A, which is also the largest class, accounting for nearly 80% of GPCR genes (Davies *et al.*, 2007). We, however, have further expanded this set by curating more data from the literature to include seven new datasets for class A, a new predictor for the B1 class (UniProt ID: Q16602), one for class C (UniProt ID: Q14833) and one for an orphan GPCR (UniProt ID:Q96LB2). In total, bioactivity data for 36 different GPCRs were collected, making this the most comprehensive dataset to date.

The datasets are composed of small inorganic molecules represented as Simplified Molecular Input Line Entry System (SMILES) strings with their respective experimental bioactivity measurement in molar. The datasets have different sizes ranging from 718 (receptor UniProt ID: P41180, Extracellular calcium-sensing receptor) to over 1 million compounds (receptor UniProt ID: P08912, Muscarinic acetylcholine receptor M5).

The collections were made by searching on the PubChem server, for the UniProt IDs, belonging to GPCRs receptors of medical interest, according to the literature (for more information please check Supplementary Table S1). We collected all ligands available at section ‘Tested compounds’ on the PubChem protein webpage, belonging to the receptors. Following the acquisition of data, all datasets were curated, with only ligands containing SMILES and bioactivity measurements (*K*i, *K*d, IC50 or EC50), as done previously (Burggraaff *et al.*, 2020; Wu *et al.*, 2018, 2019). We also removed ligands with large experimental deviations between multiple experiments (see Supplementary Table S1 for final numbers of ligands after filtering). We considered bioactivity as −log10(activity), where activity is the raw bioactivity in molar concentration. For example, a molecule with *K*i of 1 µM would have a bioactivity value of six. This transformation allows for a comparison between different bioactivity measures (Bento *et al.*, 2014; Overington *et al.*, 2006) and has been used before (Wu *et al.*, 2018, 2019). The GPCRs included by this work cover four classes (A, B1, C and F) and two receptors described as orphans. A complete description of GPCRs considered in this work, their classes, and number of compounds with available bioactivity is described in Supplementary Table S1.

### 2.2 Feature engineering

Two main sets of molecular descriptors have been calculated based on the SMILES representation of the molecules and used in combination as evidence to train, test and validate machine-learning methods for predicting GPCR ligands: (i) a distance-based graph signature and (ii) general molecule chemical and topological property descriptors.

Graph-based signatures compose a general representation of biological entities, their topology and chemical composition, which have been extensively used and validated previously in different scenarios of application (Kaminskas *et al.*, 2019; Pires and Ascher, 2016; Pires *et al.*, 2013), including pharmacokinetics, toxicity and bioactivities (Pires and Ascher, 2020; Pires *et al.*, 2015, 2020). For small molecules, molecular graphs are generated by modelling atoms as nodes and their covalent bonds as edges. Atoms are labelled based on pharmacophore modelling. The following idea is applied: the distances between all pairs of atoms (nodes) are calculated, then according to a defined a range of distances (called cut-offs and defined by the sum of the bonds between the pair of atoms) and a distance step, the molecule is scanned through these distances, computing the frequency of pairs of atoms (categorized by pharmacophore type), that are close according to this distance threshold. The goal of the signatures is to represent molecule physicochemistry by extracting distance patterns from these graphs (Pires *et al.*, 2015), which are represented as cumulative distributions.

Additionally, auxiliary attributes are calculated and combined with graph-based signatures to train and test predictive methods. These refer to various molecular properties describing the general physicochemical properties of compounds, calculated using the RDKit cheminformatics library (RDKit: Open-source cheminformatics; http://www.rdkit.org, version 2018.09.3). The complete list of general properties used in pdCSM-GPCR is available in Supplementary Table S2.

### 2.3 Machine-learning methods

Prediction of compound bioactivities was framed as a regression task, with a range of different supervised learning algorithms being assessed, including Extra Trees (Geurts *et al.*, 2006), Random Forest (Breiman, 2001), Gradient Boost (Friedman, 2001) and XGBoost (Chen and Guestrin, 2016) regression. Random Forest is an ensemble method composed of a set of decision trees (forest) that use majority voting to make a prediction. The Gradient Boost algorithm creates serialized trees, where each tree tries to correct the mistakes of the previous one. The trees created on this algorithm are shallow so that the models can provide good predictions on part of the data. Extra trees are very similar to Random Forest, the main difference is that this algorithm selects its cut-point fully at random, independently of the target variable, instead of computing the locally optimal feature/split combination, for each feature under consideration. XGBoost stands for eXtreme Gradient Boosting. It has the same principle as gradient boosting, however, uses a more regularized model formalization to avoid overfitting.

The best performing models were selected based on Pearson’s, Spearman’s and Kendall’s correlation coefficients and root mean square error. The Scikit-learn library (version 0.20.3) for Python (version 2.7) (Pedregosa *et al.*, 2011) was used for training and testing the models. For all machine-learning algorithms, the parameter ‘random_state’ was set to 0, this parameter controls the random seed given to each Tree estimator at each boosting iteration. All other parameters were kept as default and no hyperparameter tuning was performed.

We evaluated the usability and reliability of pdCSM-GPCR using cross-validation protocols, low-redundancy independent blind tests and by comparing the performance with available methods. Datasets per GPCR were split into training (90%) and blind tests (10%). To guarantee low levels of similarity between training and blind tests and avoid overfitting, molecules were first clustered by similarity using Morgan fingerprints using radius =2 (Rogers and Hahn, 2010) and Butina clustering (Butina, 1999), generating molecule groups at 80% similarity. After the generation of clusters, these were randomly chosen to belong to either train or blind test, guaranteeing low levels of similarity between sets.

For each model, we employed stratified 5-, 10- and 20-fold cross-validation on the training set. Performance was also assessed on 90% of the data, after removing 10% of the worst predicted data point, to evaluate the effects of outliers in model prediction capabilities.

We compared our predictive model’s performances with WDL-RF (Wu *et al.*, 2018). The comparison was done using the datasets provided by the authors while training their models available online. Initially, SMILES for each dataset were submitted to the WDL-RF web server. The web server outputs a table containing a column with SMILES and another with the predicted bioactivity in nM. Predictions were converted to a standard value using −log10(bioactivity), consistent with what was performed by Wu *et al.* (2018). The same procedure was employed using our web server.

Identifying the best combination of attributes to reduce noise and dimensionality is challenging. In this work, we employed feature selection via a Forward Greedy approach (Caruana and Freitag, 1994).

### 2.4 Web server

The pdCSM-GPCR web server was implemented using Bootstrap 3.3.7 and via Flask framework, version 1.1.2. The 2D chemical structure depictions are generated by RDkit.

## 3 Results

Here, we present new bioactivity predictors for the study of 36 different GPCRs belonging to four classes (A, B1, C and F). We devised a range of experiments in order to better understand and contrast the molecular properties of ligands targeting different GPCRs, demonstrate the accuracy of pdCSM-GPCR models and compare their performance with other available methods.

### 3.1 Analysis of molecular properties: what makes a GPCR ligand?

In order to answer this question, we evaluated common molecular properties and substructures of potent GPCR ligands. The top 300 most potent ligands per receptor were selected or those with bioactivity >5 (meaning potency of 10 µM or higher—for receptor Q96LB2, only 87 molecules were selected). Molecular Substructure Miner (Borgelt *et al.*, 2005) was used to identify molecular substructures that were enriched in the group of potent ligands in comparison with the remainder of the dataset (Supplementary Fig. S2).

Aromatic rings and nitrogen-containing fragments were amongst the most enriched substructures in potent GPCR ligands across all classes. Our findings corroborate with van der Horst *et al.* (2009). This study also used frequent substructure mining to analyse the structural features of GPCR ligands. The largest substructures found by them involved ‘aromatic atoms and bonds’. They suggest that these findings could be linked to a symmetrical organization of lipophilicity (through aliphatic carbon atoms) around a heteroatom, which was specified as nitrogen. Strader *et al.* (1988) identified, using mutagenesis, a negatively charged aspartic acid residue in transmembrane domain 3 of the *β*-adrenoceptor. This residue is found to form a salt bridge with the ligands’ protonated amino group. The presence of the nitrogen-containing fragments also could be correlated to the importance of hydrogen donors in the interactions with their target.

Besides looking for molecular substructures, we also found a limited number of potent molecules being shared between at least different GPCRs (see Supplementary Fig. S3). The 21 ligands identified were shared between Class A receptors and, in general, their properties were consistent with what we observed for the most potent ligands across different receptors.

We also assessed common physicochemical properties of these potent ligands (Supplementary Figs S4–S11). We found that most potent ligands possessed between 20 and 40 heavy atoms, had a molecular weight between 200 and 500 daltons, <10 rotatable bonds, a polar surface area no >140 Å^2^ and a log*p* between 0 and 6 range (Ghose *et al.*, 1999). The most potent compounds also possess between 2 and 12 heteroatoms, between 2 and 6 rings and a LabuteASA in the range of 150–200 Å^2^. This is largely consistent with Lipinski’s Rule of 5 and may reflect a bias in original screening libraries used to identify these compounds. Similarly, Morphy and Rankovic (2006), evaluated the physicochemical properties for GPCRs ligands. They found that GPCRs ligands possess a median of eight rotatable bonds, median molecular weight of 450 (mean 503), median log *P*-value of 4.4 (mean 4.2) and a median for polar surface area of 67 Å^2^.

### 3.2 Developing GPCR ligand predictors

Final predictors’ models achieved Pearson’s, Spearman’s and Kendall’s correlations of up to 0.89, 0.88 and 0.70, respectively on 10-fold cross-validation (see Supplementary Table S3), which are depicted as scatter plots (Supplementary Figs S12 and S13). We also assessed our models using mean square error (MSE) to check how close our predictions were to the actual values. We reached a minimum of 0.24 and a maximum of 1.02. After 10% outlier removal, predictions improved substantially. For all receptors, the predictions reached a Pearson’s correlation above 0.74 and considering the MSE values they decreased on average 40% for all receptors. We searched for outliers in common and found one, Clotrimazole, shared between four different receptors, D (4) dopamine receptor (P21917), 5-hydroxytryptamine receptor 2C (P28335), adenosine receptor A1 (P30542) and 5-hydroxytryptamine 9 receptor 6 (P50406). This finding reflects general properties of outliers (see Supplementary Figs S22–S35) that tend to have less hydrogen bonds acceptors, hydrogen bonds donor, and negative ionizable atoms. We additionally found that other outliers tend to have also less positive ionizable atoms.

As mentioned, we also assessed the model under different cross-validation schemes stratified 5- and 20-fold cross-validation (see Supplementary Table S4) obtaining consistent results and demonstrating robustness of the models. Considering the supervised learning algorithms employed, 21 of the final models employed Random Forest and 10 Extra Trees, with the remaining 5 using XGBoost (see Supplementary Table S4). Intriguingly, Gradient Boost was not selected for any of the receptors.

The generalization capabilities of the models were further assessed via external validation, through low-redundancy independent blind tests. Histograms were built to provide the distributions of the bioactivity labels for both the training and the low-redundancy independent blind tests datasets (Supplementary Fig. S21). Despite the low level of similarity between them, their distributions were similar, and ranged from 4.5 to 9.5, and most of the molecules presented a bioactivity between six and seven. Zhang *et al.* (2015) summarized ligand affinity data in solved GPCR structures, and found that *K*i from ligands were generally values in the single-digit nM range, converting single-digit nM values using −log10(activity), we got a value of nine (meaning our datasets covered active and inactive small molecules). Predictive models for the 36 different GPCRs achieved very consistent Pearson’s correlations to those obtained under cross-validation (up to 0.89), further demonstrating their predictive capabilities (see Supplementary Table S5).

### 3.3 Feature usage

We also evaluated feature usage after feature selection per predictor. We filtered the top 10 features selected via the Forward Greedy Feature Selection approach for each of the machine-learning models and calculated commonly used features.

When considering only Class A receptors (Supplementary Fig. S14), two features were selected for nine types of receptors (out of 31 Class A receptors). These features were topological polar surface area and the presence of bicyclic fragments on the molecule (fr_bicyclic), which is consistent with the most found substructures in potent GPCR ligands. Five MOE-like approximate molecular surface area descriptors also stand out: SMR_VSA3, SMR_VSA7 (MR refers to Molecular refractivity), SlogP_VSA2, SlogP_VSA8, SlogP_VSA3 (SlogP refers to Log of the aqueous solubility), PEOE_VSA1 (refers to partial charges and surface area). SMR_VSA3 was used on eight receptor predictors, and the remaining on seven. Besides, four features encoding distance patterns were important, all involving pairs of aromatic atoms. These imply that the presence of aromatic rings on molecules is important aspects of GPCR Class A ligands. These selected features were consistent with considering all receptor types (Supplementary Fig. S15). We also added SHapley Additive exPlanations (SHAP) summary plots to illustrate feature importance (Supplementary Figs S16–S20), SHAP assigns each feature an importance value for a particular prediction (Lundberg and Lee, 2017). According to these plots, MOE-like approximate molecular surface area descriptors, also stood out as important features for the models in addition to many graph-based signatures involving aromaticity, or hydrogen bond acceptors. These findings supported our previous feature usage evaluation.

Aiming to understand the impacts of using different activity types on model performance (IC50, EC50, *K*i and *K*d), we have carried out a set of experiments training models using *K*i + *K*d values and testing them using IC50 + EC50 (and vice-versa), depending on the number of molecules available in each case. The largest subset of activity types was assigned as the training set. Given data availability, this was performed for 13 different receptors. The results showed a decrease in performance when predictors are trained and tested with different activity measures. This might be due to biases in dataset distributions, limited dataset sizes as well as inherent differences between bioactivity measurements (Supplementary Fig. S37 for performance information and Supplementary Fig. S38 for molecular activity distribution).

### 3.4 Comparative performance

**Fig. 2.**
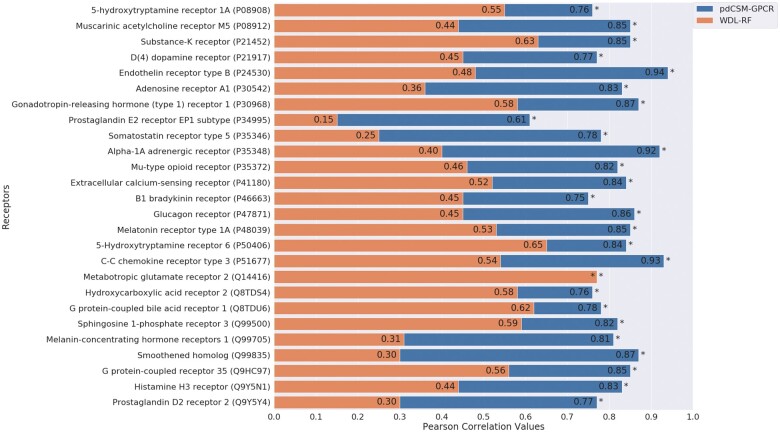
Performance comparison between pdCSM-GPCR and Wu *et al.* (2018) (WDL-RF) through Pearson correlation. *Indicates that the pdCSM-GPCR significantly outperforms (*P*-value <0.001 using a Fisher’s *Z* transformation). **Pearson Correlation values were 0.75 for pdCSM-GPCR and 0.77 for WDL-RF

In order to put our results into context, we compared the performance of our predictors with methods published previously (Wu *et al.*, 2018) (see Supplementary Table S6 for information regarding overlap between molecules used in pdCSM-GPCR and WDL-RF). The results are indicated in Figure 2, which shows that our predictors outperformed the alternative methods on almost all GPCR datasets, with statistically significant differences, except for the receptor Q14416 in which performances were very similar. The performances obtained in our models were comparable to the cross-validation performances, increasing our confidence in the method’s generalization capabilities. We also plotted scatter plots for this step (Supplementary Fig. S39 for our model’s performance and Supplementary Fig. S40 for WDL-RF performance, and a histogram which compares the activity outputs generated by the two servers, Supplementary Fig. S41). It was observed very high MSE measures for some WDL-RF models, which indicates high distance between predicted and experimental values. We have also included Spearman and Kendall metrics, results obtained with them are consistent with our previous findings.

Interestingly, when carrying out this comparative analysis, we found that our models performed significantly better. This was unexpected because we adopted for these comparisons the datasets, which were used for WDL-RF models training, and we expected at least values closer to the ones mentioned by them in the paper (Wu *et al.*, 2018). One possibility for this disparity, as already mentioned, might be the use of ‘control molecules’. For doing this step, they use for training, ligands that do not interact with the target GPCR, and they had hard set their bioactivity [−log10(activity)] to −10, which could have increased their performance artificially. The control molecules were not available at their website, and we could not use them for further testing. We, however, have also executed blind tests using a ‘non-ligand’ set. The small molecules for these ‘non-ligand’ sets were obtained through DUD-E (Mysinger *et al.*, 2012), a tool that generates decoys using active compounds. For this purpose, we used top potent ligands from our datasets. We added to our datasets 20% of decoys and the bioactivity of these were set to −1 (10 molar) (see Supplementary Fig. S42 and Table S7 to check the performance before and after adding decoys). The results, we obtained demonstrated an increase in performance in 22 models out of 36 and for 4 occurred very little variation in performance. This demonstrates the robustness of our approach, but also shows how including decoys might overestimate performance of newly developed methods.

### 3.5 pdCSM-GPCR web server

The pdCSM-GPCR web server was designed to provide a user-friendly and quick web interface to predict bioactivity for GPCR ligands. The web server allows users to submit a single compound SMILE or upload a list of them. Users can then choose which classes of receptor they want to generate bioactivity predictions for. When just a single compound is submitted, in addition to the bioactivity prediction result (in µM), the result’s page also includes a molecule depiction and general molecular properties of the compound. When multiple compounds are submitted, the prediction results are displayed in an interactive table. All results can be downloaded as a comma-separated values file (Supplementary Fig. S36).

## 4 Conclusion

We described the development of a new comprehensive computational platform for predicting the bioactivity of GPCRs ligands, based on graph signatures, which significantly outperformed alternative methods. Given the importance of GPCRs in many diseases, and that the previous methods of ligands predictions achieved poor results during evaluation, we believe that our tools will be widely used and will facilitate the GPCR drugs development process by enabling fast screening, evaluation and prioritization of compounds. Our models were also scalable, being capable of handling large datasets, an important requirement for screening initiatives.

Our results support the idea that the lack of elucidated structure for receptors is not a constraint for the development of ligand predictors. And the same procedure could be used for the development of any other receptor, which already had been screened for new ligands, such as kinases, which also composes a great family of proteins extremely important for human biochemistry (Manning, 2002).

Our predictors are all regression models with actual numeric outputs. This is of great importance during drug development because it allows the prioritization of ligands. Through prioritization, the process of finding new molecules can be faster and less costly (Schuffenhauer and Jacoby, 2004). Furthermore, we demonstrated that graph-based signatures combined with other general molecular physicochemical characteristics can be used to model ligand bioactivity applied to GPCRs drug discovery.

We have also evaluated common features of potent GPCRs ligands and found that aromatic rings and nitrogen-containing fragments were amongst the most enriched substructures across all GPCR classes. These correlated with important features to enable key interactions between ligand and transmembrane parts of GPCRs. These findings also illustrate the importance of interpretability of machine-learning models, from which insights about what makes GPCRs ligands can be drawn. We also observed that using control ligands, with arbitrary low affinities assigned, leads to an overestimation of performance, which decreases reliability of model assessment.

We have implemented pdCSM-GPCR as a user-friendly web server that will enable researchers to enrich molecule libraries for screening and support the rational design of GPCR ligands.

## Funding

This work was supported by a Melbourne Research Scholarship (to J.P.L.V.). D.B.A. and D.E.V.P. were funded by a Newton Fund RCUK-CONFAP Grant awarded by The Medical Research Council (MR/M026302/1). D.B.A. was supported by the Wellcome Trust (grant 093167/Z/10/Z) and an Investigator Grant from the National Health and Medical Research Council (NHMRC) of Australia (GNT1174405). Supported in part by the Victorian Government’s Operational Infrastructure Support Program. This study was financed in part by the Coordenação de Aperfeiçoamento de Pessoal de Nível Superior—Brasil (CAPES)—Finance Code 001.

*Conflict of Interest*: none declared.

## Supplementary Material

vbab031_Supplementary_Data
