# Supplementary material for: pdCSM-GPCR: predicting potent GPCR ligands with graph-based signatures
Source: Bioinform Adv. 2021 Nov 10;1(1):vbab031. doi: 10.1093/bioadv/vbab031 (PMC8651072; doi:10.1093/bioadv/vbab031)
Supplement: vbab031_Supplementary_Data [file vbab031_supplementary_data.pdf]

SUPPLEMENTARY MATERIALS

pdCSM-GPCR: predicting potent GPCR ligands with graph-based signatures

João Paulo L. Velloso [1,2,3,4,8], David B. Ascher [2,3,4,5,7*], Douglas E. V. Pires [2,3,4,6*]

[1] Instituto René Rachou, Fundação Oswaldo Cruz, Belo Horizonte, 30190-002, Brazil

[2] Structural Biology and Bioinformatics, Department of Biochemistry, University of Melbourne, Melbourne, Victoria, Australia

[3] Systems and Computational Biology, Bio21 Institute, University of Melbourne,Melbourne, Victoria, Australia

[4] Computational Biology and Clinical Informatics, Baker Heart and Diabetes Institute, Melbourne, Victoria, Australia

[5] Baker Department of Cardiometabolic Health, Melbourne Medical School, University of Melbourne, Melbourne, Victoria, Australia

[6] School of Computing and Information Systems, University of Melbourne, Melbourne, Victoria, Australia

[7] Department of Biochemistry, University of Cambridge, 80 Tennis Ct Rd, Cambridge CB2 1GA

[8] Department of Bioinformatics, Universidade Federal de Minas Gerais, Belo Horizonte, 31270-901, Brazil

*To whom correspondence should be addressed D.E.V.P. Email: douglas.pires@unimelb.edu.au. Correspondence may also be addressed to D.B.A. Tel: +61 90354794; Email: david.ascher@unimelb.edu.au.

TABLES

Table S1: Complete description of GPCRs considered in this work, their classes, and number of compounds with available bioactivity

Protein name	UniProt ID	Family	Subfamily	Medical interest	#Ligands collected	#Ligands after filtering
Muscarinic acetylcholine receptor M4	P08173	A	Aminergic receptors	Parkinson's disease (https://doi.org/10.1016/j.pharmthera.2007.09.009).	1097720	978
5-hydroxytryptamine receptor 1A	P08908	A	Aminergic receptors	Neuropsychiatric disorders such as anxiety, depression, and schizophrenia.	135544	3790
Muscarinic acetylcholine receptor M5	P08912	A	Aminergic receptors	Tobacco and cannabis dependence (10.1186/1471-2156-8-46).	1097830	959
Muscarinic acetylcholine receptor M5	P0DMS8	A	Aminergic receptors	Rheumatoid arthritis.	10929	3513
Muscarinic acetylcholine receptor M3	P20309	A	Aminergic receptors	Type 2 diabetes (https://doi.org/10.1016/j.cmet.2006.04.009)	6786	2008
Substance-K receptor	P21452	A	Peptide receptors	Inflammatory and pain responses (https://doi.org/10.1016/j.neulet.2005.06.011).	3153	922
D(4) dopamine receptor	P21917	A	Aminergic receptors	Parkinson's disease, schizophrenia, mania, depression, substance abuse, and eating disorders (https://doi.org/10.1021/cr050263h).	6251	2335

Endothelin receptor type B	P24530	A	Peptide receptors	Hirschsprung's disease (10.1074/jbc.273.18.11378).	1805	987
5-hydroxytryptamine receptor 2C	P28335	A	Aminergic receptors	Neuroendocrine responses to stress (10.1523/JNEUROSCI.2584-06.2007)	8179	3118
Adenosine receptor A2b	P29275	A	Nucleotide receptors	Asthma and gastrointestinal disorders (https://doi.org/10.1016/B978-0-12-803724-9.00001-6).	6714	2109
Adenosine receptor A1	P30542	A	Nucleotide receptors	Cardiac ischemia, stroke, hypertension, and epilepsy.	13364	3833
Gonadotropin-releasing hormone (type 1) receptor 1	P30968	A	Peptide receptors	Hypogonadotropic hypogonadism (https://doi.org/10.1038/ng0198-14).	3017	1373
Prostaglandin E2 receptor EP1 subtype	P34995	A	Lipid receptors	Treatment of neuropathic pain (10.1097/00000539-200110000-00043).	1631	741
Somatostatin receptor type 5	P35346	A	Peptide receptors	Inhibit the release of many hormones and other secretory proteins (10.1159/000054651).	1361	747
Alpha-1A adrenergic receptor	P35348	A	Aminergic receptors	Noradrenergic modulation of olfactory driven behaviours (10.1113/jphysiol.2012.248591).	4034	1898
Mu-type opioid	P35372	A	Peptide	Morphine-induced	691466	5275

receptor			receptors	analgesia and itch (10.1016/j.cell.2011.08.043).		
B1 bradykinin receptor	P46663	A	Peptide receptors	Inflammatory injuries that follow ischaemia and reperfusion (10.4049/jimmunol.172.4.2542).	1491	756
P2 purinoceptor subtype Y1	P47900	A	Nucleotide receptors	Platelet shape and platelet aggregation (10.1042/bj3360513).	1200	568
Melatonin receptor type 1A	P48039	A	Peptide receptors	Circadian and neuroendocrine disorders (0.1006/geno.1995.1056).	3003	1043
5-Hydroxytryptamine receptor 6	P50406	A	Aminergic receptors	Learning process and memory (https://doi.org/10.1016/B978-0-12-800836-2.00011-8).	8230	3044
C-C chemokine receptor type 3	P51677	A	Protein receptors	Binds and responds to a variety of chemokines, HIV infection (10.1016/s0092-8674(00)81313-6 0).	1675	1131
Hydroxycarboxylic acid receptor 2	Q8TDS4	A	Alicarboxylic acid receptors	Dyslipidemia (10.2217/pgs.15.79).	1664	504
G protein-coupled bile acid receptor 1	Q8TDU6	A	Steroid receptors	Immune and inflammatory liver diseases (10.1002/hep.24525).	1014	443
Mas-related G protein-coupled receptor X1	Q96LB2	A	Orphan receptors	Modulation of nociception (https://doi.org/10.1096/fj.202001667RR).	936090	93

Sphingosine 1-phosphate receptor 3	Q99500	A	Lipid receptors	Glioblastoma (10.1016/j.freeradbiomed.2005.09.015).	232193	1088
Melanin-concentrating hormone receptors 1	Q99705	A	Peptide receptors	Obesity (10.2174/092986708784049621).	8628	3721
Sphingosine 1-phosphate receptor 5	Q9H228	A	Lipid receptors	Huntington's disease (10.1093/hmg/ddy153).	782	417
G protein-coupled receptor 35	Q9HC97	A	Orphan receptors	Albright hereditary osteodystrophy-like phenotype (10.1111/j.1399-0004.2004.00363.x).	293497	480
Histamine H3 receptor	Q9Y5N1	A	Aminergic receptors	Attention deficit hyperactivity disorder, Alzheimer's disease and schizophrenia (10.1038/bjp.2008.147).	6873	3597
Prostaglandin D2 receptor 2	Q9Y5Y4	A	Lipid receptors	Inflammatory disease of the upper airways (10.1016/j.prostaglandins.2003.12.002).	5017	2749
Glucagon receptor	P47871	B1	Peptide receptors	Type 2 diabetes (10.1038/ng0395-299).	2053	1006
Calcitonin gene-related peptide type 1 receptor	Q16602	B1	Peptide receptors	Migraine (10.1177/1756285610388343).	1663	757
Extracellular calcium-sensing receptor	P41180	C	Ion receptors	Ischemic brain injury (10.1002/acn3.118).	718	535
Metabotropic	Q14416	C	Amino acid	Pain mechanisms and	2475	1168

glutamate receptor 2			receptors	behavioral modulation (https://doi.org/10.3389/fnmol.2018.00383).		
Metabotropic glutamate receptor 4	Q14833	C	Amino acid receptors	Parkinson Disease (10.1007/s11481-016-9655-z).	3457	579
Smoothened homolog	Q99835	F	Protein receptors	Carcinogenesis (https://doi.org/10.1016/j.lfs.2020.117302).	1366	718

Table S2: All general descriptors used as auxiliary signatures

**Name**	**Description**	**Reference**
HeavyAtomCount	Number of Non-Hydrogen atoms in a given molecule	
MolLogP	Particular ratio of the concentrations of a solute between the two solvents (a biphase of liquid phases), one of the solvents is water and the other is a non-polar solvent	(Wildman and Crippen, 1999)
NumHeteroatoms	Number of heavy atoms a molecule. (Non-hydrogens)	
NumRotatableBonds	Number of Rotatable Bonds	
RingCount	Number of rings.	
TPSA	Topological polar surface area (TPSA) of a molecule is defined as the surface sum over all polar atoms, primarily oxygen and nitrogen, also including their attached hydrogen atom.	(Ertl et al., 2000)
LabuteASA	Labute's Approximate Surface Area	(Labute, 2000)
MolWt	Molecular Weight	
Fcount	Number of Fluorine atoms	
Tox	Toxicophores	(Kazius et al., 2005)
BalabanJ:	Balaban's connectivity topological index	(Balaban, 1982)
BertzCT	A topological index meant to quantify “complexity” of molecules. Consists of a sum of two terms, one representing the complexity of the bonding, the other representing the complexity of the distribution of heteroatoms.	(Bertz, 1981)
Chi0, Chi1	Atomic connectivity index (order 0) . This is calculated as the sum of 1/sqrt(di) overall heavy atoms i with di > 0.	(Hall and Kier, 2007)
Chi0n - Chi4n		(Hall and Kier, 2007)
Chi0v - Chi4v	Atomic connectivity index (order 1). This is calculated as the sum of 1/sqrt(didj) overall bonds between heavy	(Hall and Kier, 2007)

	atoms i and j where i < j.	
chi0v_C, chi1v_C	Carbon valence connectivity index (order 0). This is calculated as the sum of 1/sqrt(vi) overall carbon atoms i with vi > 0.	(Hall and Kier, 2007)
HallKierAlpha		(Hall and Kier, 2007)
Kappa1- Kappa3		(Hall and Kier, 2007)
PEOE_VSA1 - PEOE_VSA14	MOE-type descriptors using partial charges and surface area contributions	
SMR_VSA1 - SMR_VSA10	MOE-type descriptors using MR contributions and surface area contributions	
SlogP_VSA1 - SlogP_VSA12	MOE-type descriptors using SLogP contributions and surface area contributions	
EState_VSA1 - EState_VSA11	MOE-type descriptors using EState indices and surface area contributions	
VSA_EState1 - VSA_EState10	MOE-type descriptors using surface area contributions and Estate indices	
Organic functions	fr_Al_COO,fr_Al_OH,fr_Al_OH_noTert,fr_ArN,fr_Ar_COO,fr_Ar_N,fr_Ar_NH,fr_Ar_OH,fr_COO,fr_COO2,fr_C_O,fr_C_O_noCOO,fr_C_S,fr_HOCCN,fr_Imine,fr_NH0,fr_NH1,fr_NH2,fr_N_O,fr_Ndealkylation1,fr_Ndealkylation2,fr_Nhpyrrole,fr_SH,fr_aldehyde,fr_alkyl_carbamate,fr_alkyl_halide,fr_allylic_oxid,fr_amide,fr_amidine,fr_aniline,fr_aryl_methyl,fr_azide,fr_azo,fr_barbitur,fr_benzene,fr_benzodiazepine,fr_bicyclic,f_diazo,fr_dihydropyridine,fr_epoxide,fr_ester,fr_ether,fr_furan,fr_guanido,fr_halogen,fr_hdrzine,fr_hdrzone,fr_imidazole,fr_imide,fr_isocyan,fr_isothiocyan,fr_ketone,fr_ketone_Topliss,fr_lactam,fr_lactone,fr_methoxy,fr_morpholine,fr_nitrile,fr_nitro,fr_nitro_arom,fr_nitro_arom_nonortho,fr_nitroso,fr_oxazole,fr_oxime,fr_para_hydroxylation,fr_phenol,fr_phenol_noOrthoHbond,fr_phos_acid,fr_phos_ester,fr_piperdine,fr_piperzine,fr_priamide,fr_prisulfonamd,	

	fr_pyridine,fr_quatN,fr_sulfide,fr_sulfonamd,fr_sulfone,fr_term_acetylene,fr_tetrazole,fr_thiazole,fr_thiocyan,fr_thiophene,fr_unbrch_alkane,fr_urea	

Table S3: Final Predictors performance on 10-fold cross-validation

Receptor	pearson	spearman	kendall	MSE	pearson after outlier removal	spearman after outlier removal	kendall after outlier removal	MSE after outlier removal
Muscarinic acetylcholine receptor M4-(P08173)	0.77	0.75	0.57	0.59	0.87	0.83	0.65	0.29
5-hydroxytryptamine receptor 1A-(P08908)	0.75	0.74	0.56	0.60	0.85	0.83	0.64	0.31
Muscarinic acetylcholine receptor M5-(P08912)	0.76	0.75	0.56	0.52	0.87	0.82	0.63	0.23
Muscarinic acetylcholine receptor M5-(P0DMS8)	0.79	0.80	0.62	0.55	0.91	0.90	0.72	0.23
Muscarinic acetylcholine receptor M3-(P20309)	0.85	0.85	0.68	0.70	0.94	0.94	0.77	0.31
Substance-K receptor-(P21452)	0.85	0.85	0.67	0.52	0.92	0.92	0.75	0.26
D(4) dopamine receptor-(P21917)	0.69	0.70	0.52	0.53	0.83	0.82	0.63	0.27
Endothelin receptor type B-(P24530)	0.89	0.86	0.68	0.34	0.94	0.92	0.75	0.18
5-hydroxytryptamine receptor 2C-(P28335)	0.72	0.71	0.53	0.52	0.83	0.82	0.62	0.29
Adenosine receptor A2b-(P29275)	0.79	0.80	0.63	0.46	0.91	0.90	0.73	0.20
Adenosine receptor A1-(P30542)	0.76	0.75	0.57	0.50	0.87	0.84	0.65	0.23
Gonadotropin-releasing hormone (type 1) receptor 1-(P30968)	0.80	0.79	0.60	0.52	0.88	0.88	0.69	0.29
Prostaglandin E2 receptor EP1 subtype-(P34995)	0.78	0.77	0.59	0.51	0.88	0.85	0.67	0.27
Somatostatin receptor type 5-(P35346)	0.84	0.82	0.63	0.45	0.91	0.89	0.71	0.25
Alpha-1A adrenergic receptor-(P35348)	0.78	0.78	0.59	0.63	0.87	0.87	0.68	0.35
Mu-type opioid receptor-(P35372)	0.87	0.88	0.70	0.62	0.94	0.94	0.78	0.29
Extracellular calcium-sensing receptor-(P41180)	0.74	0.74	0.55	0.50	0.85	0.83	0.64	0.25
B1 bradykinin receptor-(P46663)	0.77	0.74	0.55	0.55	0.87	0.84	0.64	0.38
Glucagon receptor-(P47871)	0.83	0.80	0.61	0.38	0.90	0.87	0.69	0.20
P2 purinoceptor subtype Y1-(P47900)	0.73	0.75	0.56	0.56	0.86	0.84	0.65	0.25
Melatonin receptor type 1A-(P48039)	0.73	0.73	0.54	1.02	0.82	0.80	0.60	0.62
5-Hydroxytryptamine receptor 6-(P50406)	0.79	0.78	0.60	0.53	0.89	0.88	0.69	0.28
C-C chemokine receptor type 3-(P51677)	0.84	0.83	0.65	0.49	0.90	0.90	0.71	0.27
Metabotropic glutamate receptor 2-(Q14416)	0.84	0.85	0.67	0.26	0.91	0.91	0.73	0.12
Metabotropic glutamate receptor 4-(Q14833)	0.80	0.58	0.431	1.071	0.91	0.67	0.50	0.24

Calcitonin gene-related peptide type 1 receptor-(Q16602)	0.83	0.83	0.64	0.83	0.91	0.91	0.73	0.43
Hydroxycarboxylic acid receptor 2-(Q8TDS4)	0.67	0.67	0.48	0.52	0.78	0.78	0.58	0.32
G protein-coupled bile acid receptor 1-(Q8TDU6)	0.70	0.70	0.50	0.70	0.80	0.79	0.58	0.44
Mas-related G protein-coupled receptor X1-(Q96LB2)	0.69	0.49	0.36	0.47	0.74	0.70	0.52	0.25
Sphingosine 1-phosphate receptor 3-(Q99500)	0.78	0.77	0.59	0.43	0.89	0.87	0.68	0.21
Melanin-concentrating hormone receptors 1-(Q99705)	0.77	0.74	0.57	0.50	0.88	0.85	0.66	0.25
Smoothened homolog-(Q99835)	0.74	0.73	0.55	0.27	0.85	0.84	0.65	0.13
Sphingosine 1-phosphate receptor 5-(Q9H228)	0.86	0.85	0.67	0.44	0.93	0.91	0.75	0.21
G protein-coupled receptor 35-(Q9HC97)	0.84	0.76	0.59	0.24	0.92	0.81	0.64	0.11
Histamine H3 receptor-(Q9Y5N1)	0.79	0.79	0.61	0.47	0.89	0.87	0.69	0.23
Prostaglandin D2 receptor 2-(Q9Y5Y4)	0.74	0.73	0.54	0.54	0.84	0.82	0.62	0.30

mse: refers to the mean squared error of an estimator, it measures the average squared difference between the estimated values and what is estimated.

Table S4: Final Predictors cross-validation results using Pearson correlations on 5, 10 and 20-fold

**Receptor**	**Final algorithm**	**Cross-validation 5-fold**	**Cross-validation 10-fold**	**Cross-validation 20-fold**
Muscarinic acetylcholine receptor M4-(P08173)	Extra Trees	0.76	0.77	0.76
5-hydroxytryptamine receptor 1A-(P08908)	Random Forest	0.76	0.75	0.76
Muscarinic acetylcholine receptor M5-(P08912)	Extra Trees	0.77	0.76	0.77
Muscarinic acetylcholine receptor M5-(P0DMS8)	Random Forest	0.79	0.79	0.79
Muscarinic acetylcholine receptor M3-(P20309)	Extra Trees	0.85	0.85	0.85
Substance-K receptor-(P21452)	Random Forest	0.84	0.85	0.85
D(4) dopamine receptor-(P21917)	Random Forest	0.71	0.69	0.71
Endothelin receptor type B-(P24530)	Extra Trees	0.87	0.89	0.88
5-hydroxytryptamine receptor 2C-(P28335)	Random Forest	0.74	0.72	0.73
Adenosine receptor A2b-(P29275)	Random Forest	0.81	0.79	0.82
Adenosine receptor A1-(P30542)	Random Forest	0.77	0.76	0.77
Gonadotropin-releasing hormone (type 1) receptor 1-(P30968)	Random Forest	0.80	0.80	0.80
Prostaglandin E2 receptor EP1 subtype-(P34995)	Random Forest	0.79	0.78	0.80
Somatostatin receptor type 5-(P35346)	Extra Trees	0.85	0.84	0.84
Alpha-1A adrenergic receptor-(P35348)	Extra Trees	0.78	0.78	0.79
Mu-type opioid receptor-(P35372)	Extra Trees	0.87	0.87	0.87
Extracellular calcium-sensing receptor-(P41180)	XGBoost	0.75	0.74	0.75
B1 bradykinin receptor-(P46663)	XGBoost	0.75	0.77	0.76
Glucagon receptor-(P47871)	Extra Trees	0.84	0.83	0.83
P2 purinoceptor subtype Y1-(P47900)	Random Forest	0.71	0.73	0.74
Melatonin receptor type 1A-(P48039)	Random Forest	0.76	0.73	0.76
5-Hydroxytryptamine receptor 6-(P50406)	Random Forest	0.80	0.79	0.80
C-C chemokine receptor type 3-(P51677)	Random Forest	0.86	0.84	0.86
Metabotropic glutamate receptor 2-(Q14416)	Random Forest	0.85	0.84	0.85
Metabotropic glutamate receptor 4-(Q14833)	Random Forest	0.74	0.80	0.72

Calcitonin gene-related peptide type 1 receptor-(Q16602)	Random Forest	0.84	0.83	0.84
Hydroxycarboxylic acid receptor 2-(Q8TDS4)	XGBoost	0.67	0.67	0.67
G protein-coupled bile acid receptor 1-(Q8TDU6)	XGBoost	0.70	0.70	0.70
Mas-related G protein-coupled receptor X1-(Q96LB2)	XGBoost	0.68	0.69	0.69
Sphingosine 1-phosphate receptor 3-(Q99500)	Random Forest	0.76	0.78	0.77
Melanin-concentrating hormone receptors 1-(Q99705)	Random Forest	0.78	0.77	0.78
Smoothened homolog-(Q99835)	Random Forest	0.73	0.74	0.73
Sphingosine 1-phosphate receptor 5-(Q9H228)	Extra Trees	0.86	0.86	0.86
G protein-coupled receptor 35-(Q9HC97)	Random Forest	0.85	0.84	0.84
Histamine H3 receptor-(Q9Y5N1)	Extra Trees	0.80	0.79	0.80
Prostaglandin D2 receptor 2-(Q9Y5Y4)	Random Forest	0.76	0.74	0.75

Table S5: Blind test results

Receptor	Blind test using all Features (r)	Blind test after feature selection (r)
Muscarinic acetylcholine receptor M4-(P08173)	0.59	0.59
5-hydroxytryptamine receptor 1A-(P08908)	0.63	0.67
Muscarinic acetylcholine receptor M5-(P08912)	0.63	0.69
Muscarinic acetylcholine receptor M5-(P0DMS8)	0.76	0.78
Muscarinic acetylcholine receptor M3-(P20309)	0.85	0.86
Substance-K receptor-(P21452)	0.83	0.86
D(4) dopamine receptor-(P21917)	0.54	0.62
Endothelin receptor type B-(P24530)	0.85	0.85
5-hydroxytryptamine receptor 2C-(P28335)	0.70	0.72
Adenosine receptor A2b-(P29275)	0.73	0.76
Adenosine receptor A1-(P30542)	0.71	0.72
Gonadotropin-releasing hormone (type 1) receptor 1-(P30968)	0.79	0.80
Prostaglandin E2 receptor EP1 subtype-(P34995)	0.64	0.71
Somatostatin receptor type 5-(P35346)	0.71	0.75
Alpha-1A adrenergic receptor-(P35348)	0.77	0.77
Mu-type opioid receptor-(P35372)	0.80	0.80
Extracellular calcium-sensing receptor-(P41180)	0.65	0.73
B1 bradykinin receptor-(P46663)	0.79	0.81
Glucagon receptor-(P47871)	0.59	0.69
P2 purinoceptor subtype Y1-(P47900)	0.75	0.77
Melatonin receptor type 1A-(P48039)	0.60	0.68
5-Hydroxytryptamine receptor 6-(P50406)	0.77	0.78
C-C chemokine receptor type 3-(P51677)	0.82	0.84
Metabotropic glutamate receptor 2-(Q14416)	0.79	0.82
Metabotropic glutamate receptor 4-(Q14833)**	0.89	0.88

Calcitonin gene-related peptide type 1 receptor-(Q16602)	0.76	0.80
Hydroxycarboxylic acid receptor 2-(Q8TDS4)	0.54	0.63
G protein-coupled bile acid receptor 1-(Q8TDU6)	0.76	0.82
Mas-related G protein-coupled receptor X1-(Q96LB2)	0.20	0.77
Sphingosine 1-phosphate receptor 3-(Q99500)	0.52	0.68
Melanin-concentrating hormone receptors 1-(Q99705)	0.67	0.68
Smoothened homolog-(Q99835)**	0.84	0.82
Sphingosine 1-phosphate receptor 5-(Q9H228)	0.80	0.86
G protein-coupled receptor 35-(Q9HC97)	0.74	0.84
Histamine H3 receptor-(Q9Y5N1)**	0.70	0.68
Prostaglandin D2 receptor 2-(Q9Y5Y4)	0.71	0.71

**For these receptors the feature selection was incapable of improving prediction performance, and for these models we used all generated features.

Table S6: Number of molecules for training pdCSM-GPCR that overlapped with datasets from WDL-RF

Receptor	Num. of molecules used in pdCSMS-GPCR	Num. of molecules used in WDL-RF	Num. of molecules overlap	Percentage of overlap (%)
5-hydroxytryptamine receptor 1A-(P08908)	3790	2294	1666	43
Muscarinic acetylcholine receptor M5-(P08912)	959	369	261	27
Substance-K receptor-(P21452)	922	696	411	44
D(4) dopamine receptor-(P21917)	2335	1679	1251	53
Endothelin receptor type B-(P24530)	987	1019	791	80
Adenosine receptor A1-(P30542)	3833	3016	1937	50
Gonadotropin-releasing hormone (type 1) receptor 1-(P30968)	1373	1124	961	69
Prostaglandin E2 receptor EP1 subtype-(P34995)	741	236	133	17
Somatostatin receptor type 5-(P35346)	747	689	371	49
Alpha-1A adrenergic receptor-(P35348)	1898	1027	805	42
Mu-type opioid receptor-(P35372)	5275	3828	2194	41
Extracellular calcium-sensing receptor-(P41180)	535	940	378	70
B1 bradykinin receptor-(P46663)	756	452	375	49
Glucagon receptor-(P47871)	1006	1129	731	72
Melatonin receptor type 1A-(P48039)	1043	683	586	56
5-Hydroxytryptamine receptor 6-(P50406)	3044	1421	1032	33
C-C chemokine receptor type 3-(P51677)	1131	781	692	61
Metabotropic glutamate receptor 2-(Q14416)	1168	1810	506	43
Hydroxycarboxylic acid receptor 2-(Q8TDS4)	504	271	229	45
G protein-coupled bile acid receptor 1-(Q8TDU6)	443	1153	379	85
Sphingosine 1-phosphate receptor 3-(Q99500)	1088	317	220	20
Melanin-concentrating hormone receptors 1-(Q99705)	3721	2052	1781	47
Smoothened homolog-(Q99835)	718	1523	487	67
G protein-coupled receptor 35-(Q9HC97)	480	1579	369	76
Histamine H3 receptor-(Q9Y5N1)	3597	2092	1638	45
Prostaglandin D2 receptor 2-(Q9Y5Y4)	2749	641	572	20

Table S7: Performance comparison between pdCSM-GPCR with and without decoys through Pearson correlation. Green means that the model performed better when using decoys.

Receptor	Blind test without Decoys (r)	Blind test using Decoys (r)
Muscarinic acetylcholine receptor M4-(P08173)	0.59	0.66
5-hydroxytryptamine receptor 1A-(P08908)	0.67	0.93
Muscarinic acetylcholine receptor M5-(P08912)	0.69	0.72
Muscarinic acetylcholine receptor M5-(P0DMS8)	0.78	0.5
Muscarinic acetylcholine receptor M3-(P20309)	0.86	0.84
Substance-K receptor-(P21452)	0.86	0.92
D(4) dopamine receptor-(P21917)	0.62	0.79
Endothelin receptor type B-(P24530)	0.85	0.92
5-hydroxytryptamine receptor 2C-(P28335)	0.72	0.79
Adenosine receptor A2b-(P29275)	0.76	0.69
Adenosine receptor A1-(P30542)	0.72	0.59
Gonadotropin-releasing hormone (type 1) receptor 1-(P30968)	0.80	0.61
Prostaglandin E2 receptor EP1 subtype-(P34995)	0.71	0.9
Somatostatin receptor type 5-(P35346)	0.75	0.79
Alpha-1A adrenergic receptor-(P35348)	0.77	0.44
Mu-type opioid receptor-(P35372)	0.80	0.92
Extracellular calcium-sensing receptor-(P41180)	0.73	0.75
B1 bradykinin receptor-(P46663)	0.81	0.65
Glucagon receptor-(P47871)	0.69	0.93
P2 purinoceptor subtype Y1-(P47900)	0.77	0.95
Melatonin receptor type 1A-(P48039)	0.68	0.44
5-Hydroxytryptamine receptor 6-(P50406)	0.78	0.88
C-C chemokine receptor type 3-(P51677)	0.84	0.98
Metabotropic glutamate receptor 2-(Q14416)	0.82	0.88
Metabotropic glutamate receptor 4-(Q14833)**	0.89	0.52
Calcitonin gene-related peptide type 1 receptor-(Q16602)	0.80	0.78
Hydroxycarboxylic acid receptor 2-(Q8TDS4)	0.63	0.9
G protein-coupled bile acid receptor 1-(Q8TDU6)	0.82	0.26
Mas-related G protein-coupled receptor X1-(Q96LB2)	0.77	0.54
Sphingosine 1-phosphate receptor 3-(Q99500)	0.68	0.78
Melanin-concentrating hormone receptors 1-(Q99705)	0.68	0.92
Smoothened homolog-(Q99835)**	0.84	0.89
Sphingosine 1-phosphate receptor 5-(Q9H228)	0.86	0.81
G protein-coupled receptor 35-(Q9HC97)	0.84	0.84
Histamine H3 receptor-(Q9Y5N1)**	0.70	0.89
Prostaglandin D2 receptor 2-(Q9Y5Y4)	0.71	0.97

FIGURES

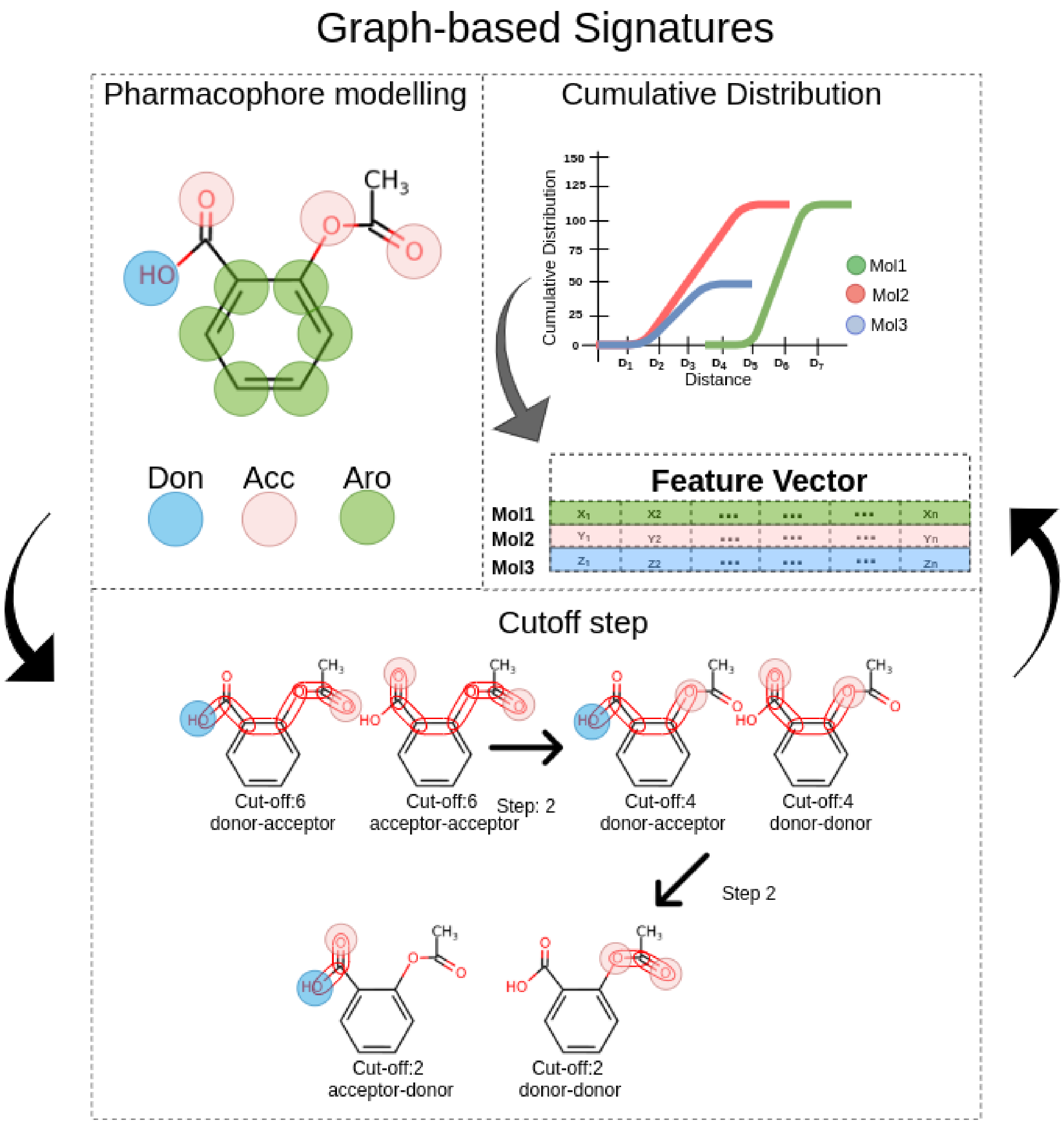

Figure S1: Modelling small molecule activity using graph-based signatures. Small molecules are modelled as unweighted, undirected graphs where nodes represent atoms and edges represent chemical bonds, with atoms labelled via pharmacophore modelling (top-left panel). Then distance between all pairs of atoms (nodes) are calculated, and according to a defined range of distances (called cut offs and defined by the sum of the bonds between the pair of atoms) and a distance step, the molecule is scanned through these distances, computing the frequency of pairs of atoms (categorized by pharmacophore type), that are close according to this distance threshold. We picture on the image a small molecule being scanned for two types of pharmacophores, hydrogen bond acceptor and donor. We started with a distance cut off of 6 bonds, and found two pairs of pharmacophores, donor-acceptor and acceptor-acceptor. Following a distance step of 2, we then used a distance cutoff of 4 and found also two pharmacophores, donor-acceptor, donor-donor. At last, we used a cut off of 2 and found also two pairs of pharmacophores, acceptor-donor, donor-donor (bottom panel). The small molecule is then represented as cumulative distributions of the pair of pharmacophores (top-right panel).

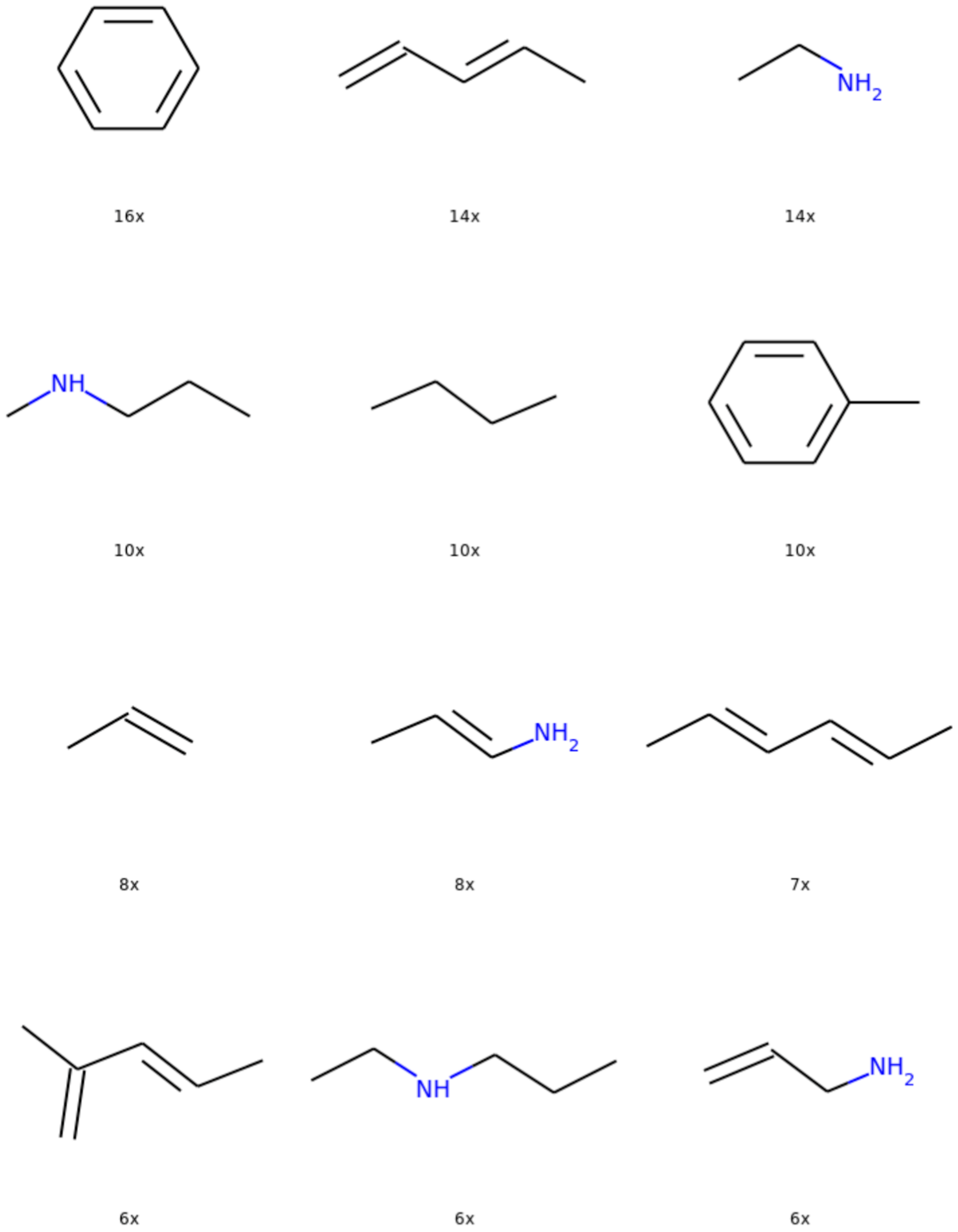

Figure S2: Distribution of the top ten most frequent fragments present on the most active ligand of all receptors.

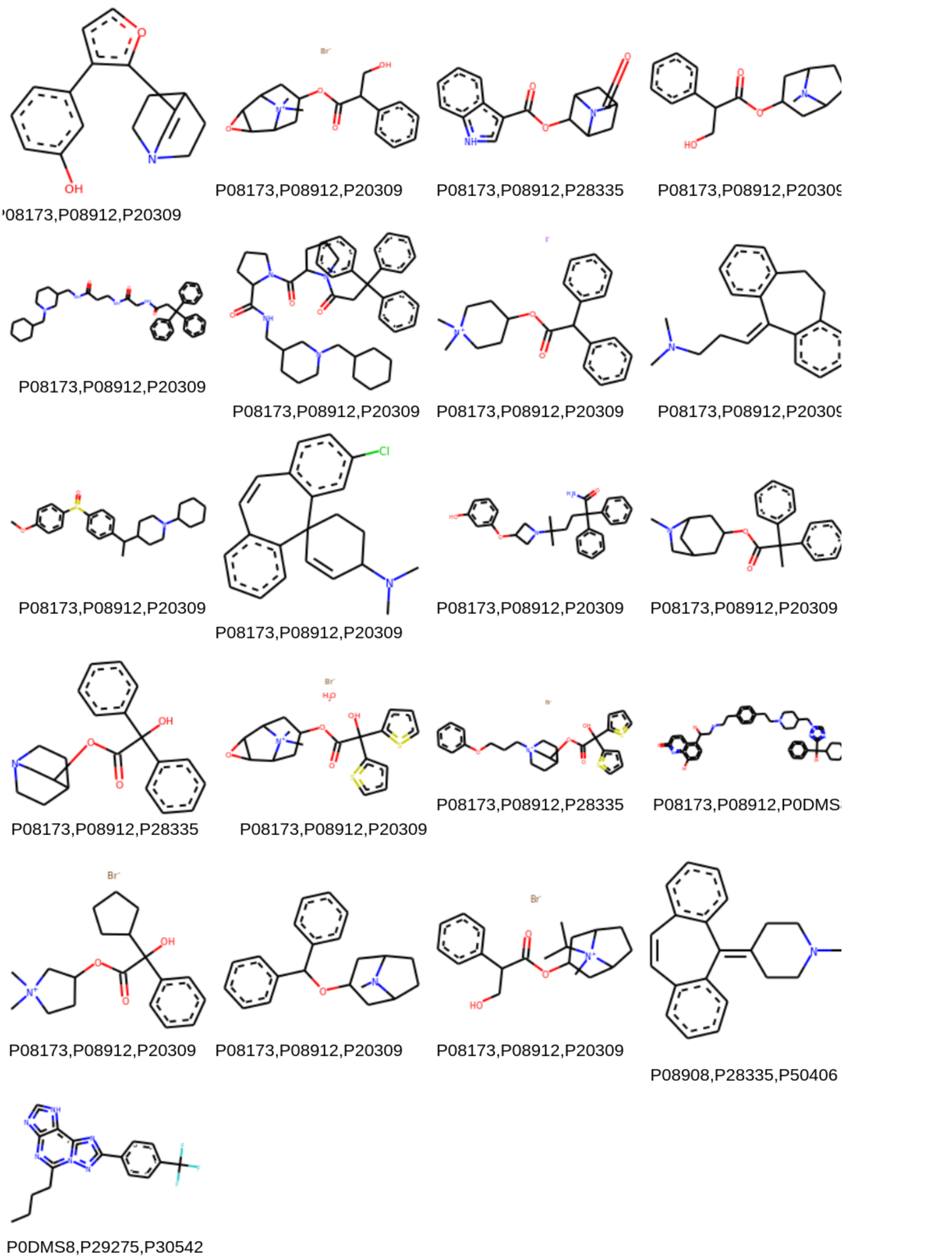

Figure S3: Shows the distribution of top potent ligands shared between at least 3 different GPCRs of all classes.

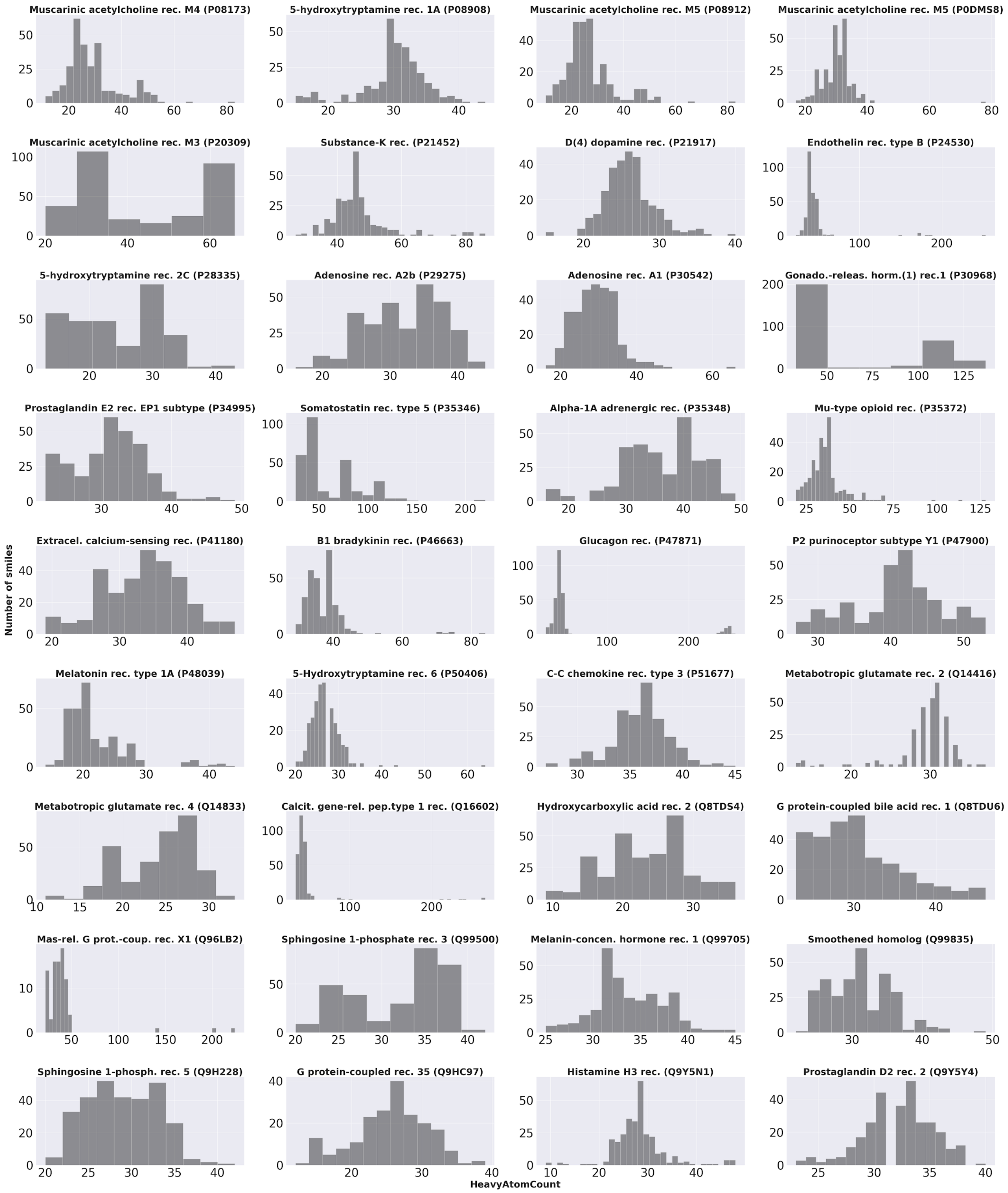

Figure S4: Potent ligands - Histograms considering heavy atoms count distribution for all datasets.

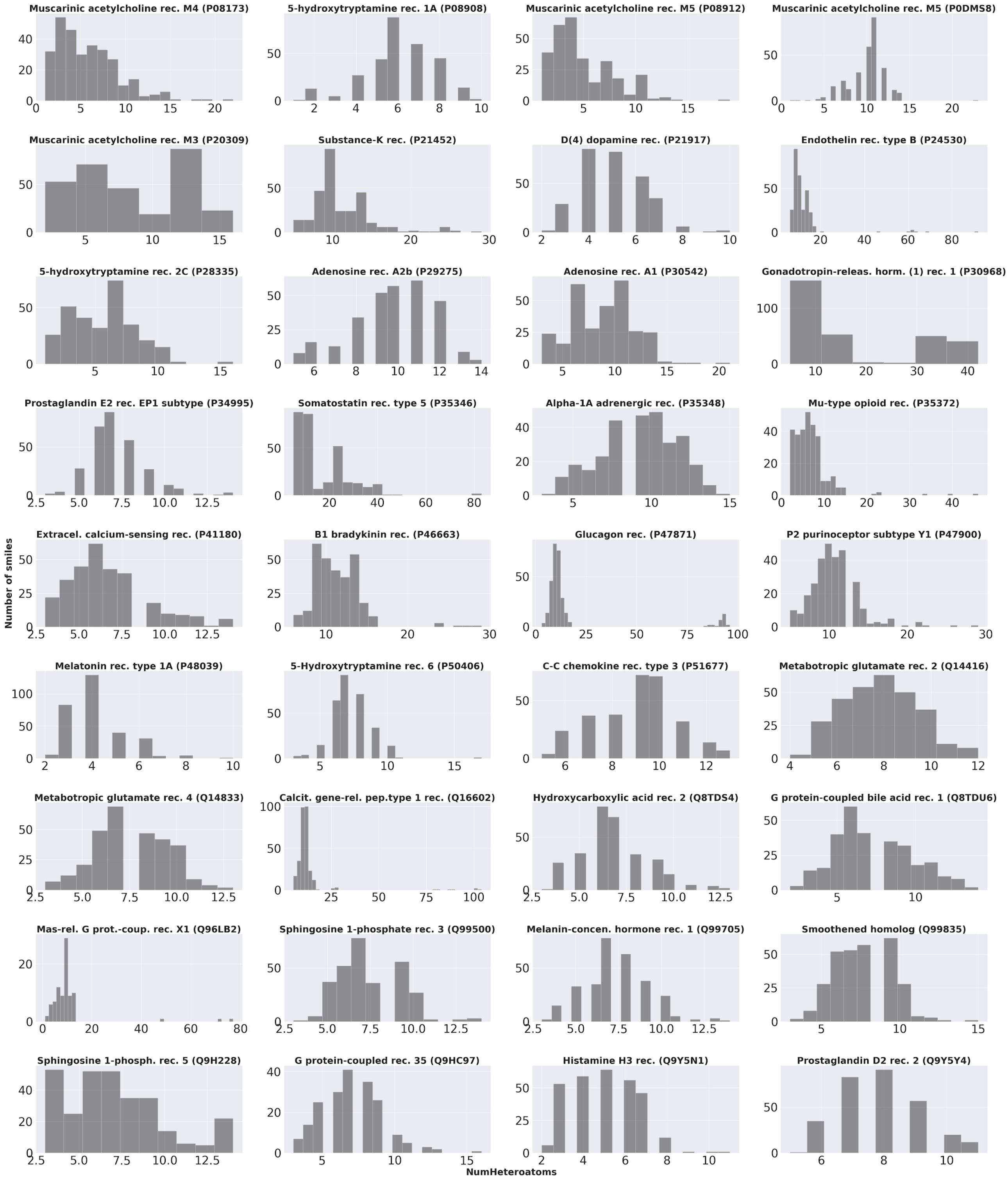

Figure S5: Potent ligands - Histograms considering number of heteroatoms distribution for all datasets.

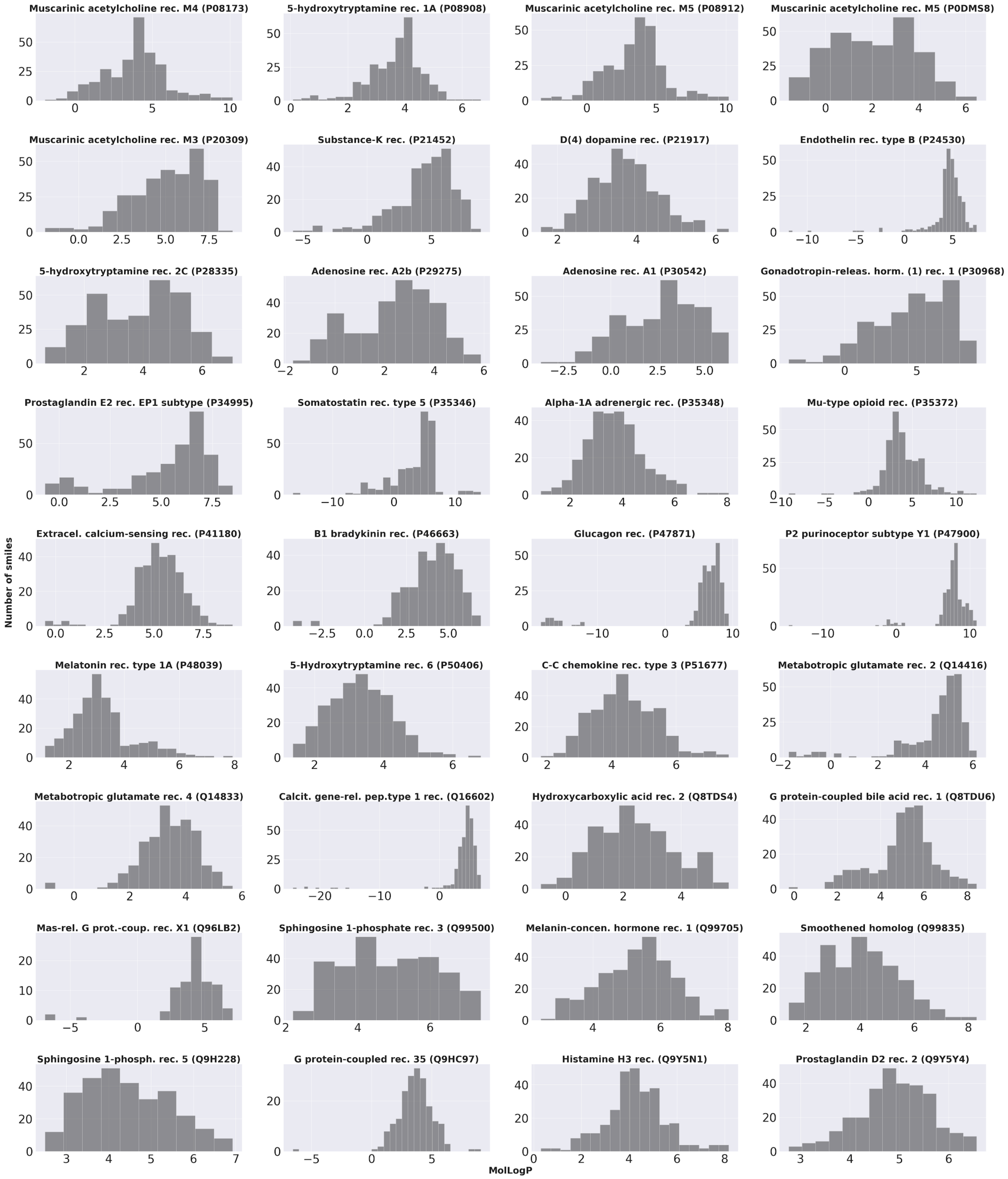

Figure S6: Potent ligands - Histograms considering log *P* distribution for all datasets.

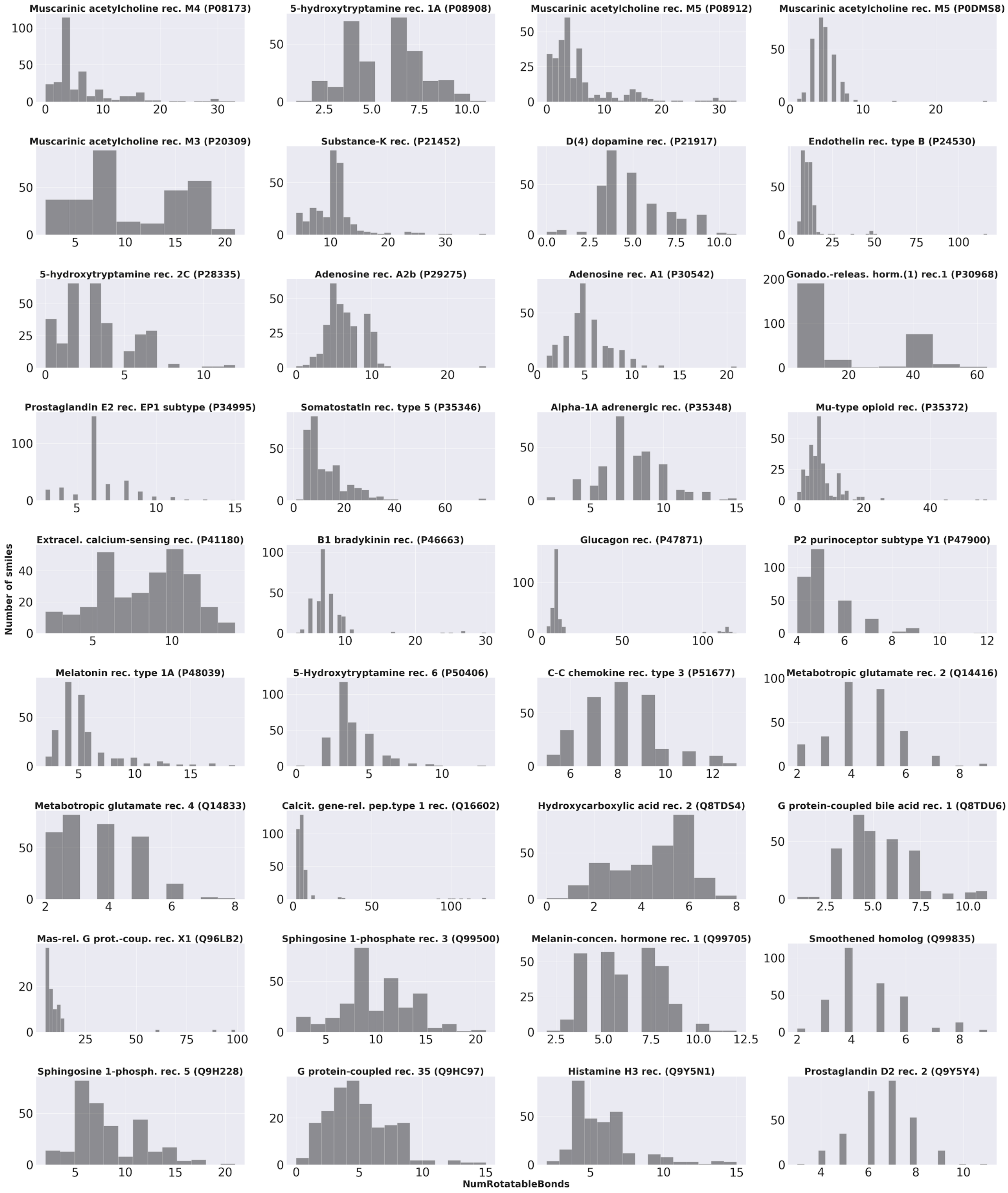

Figure S7: Potent ligands - Histograms considering number of rotatable bonds distribution for all datasets.

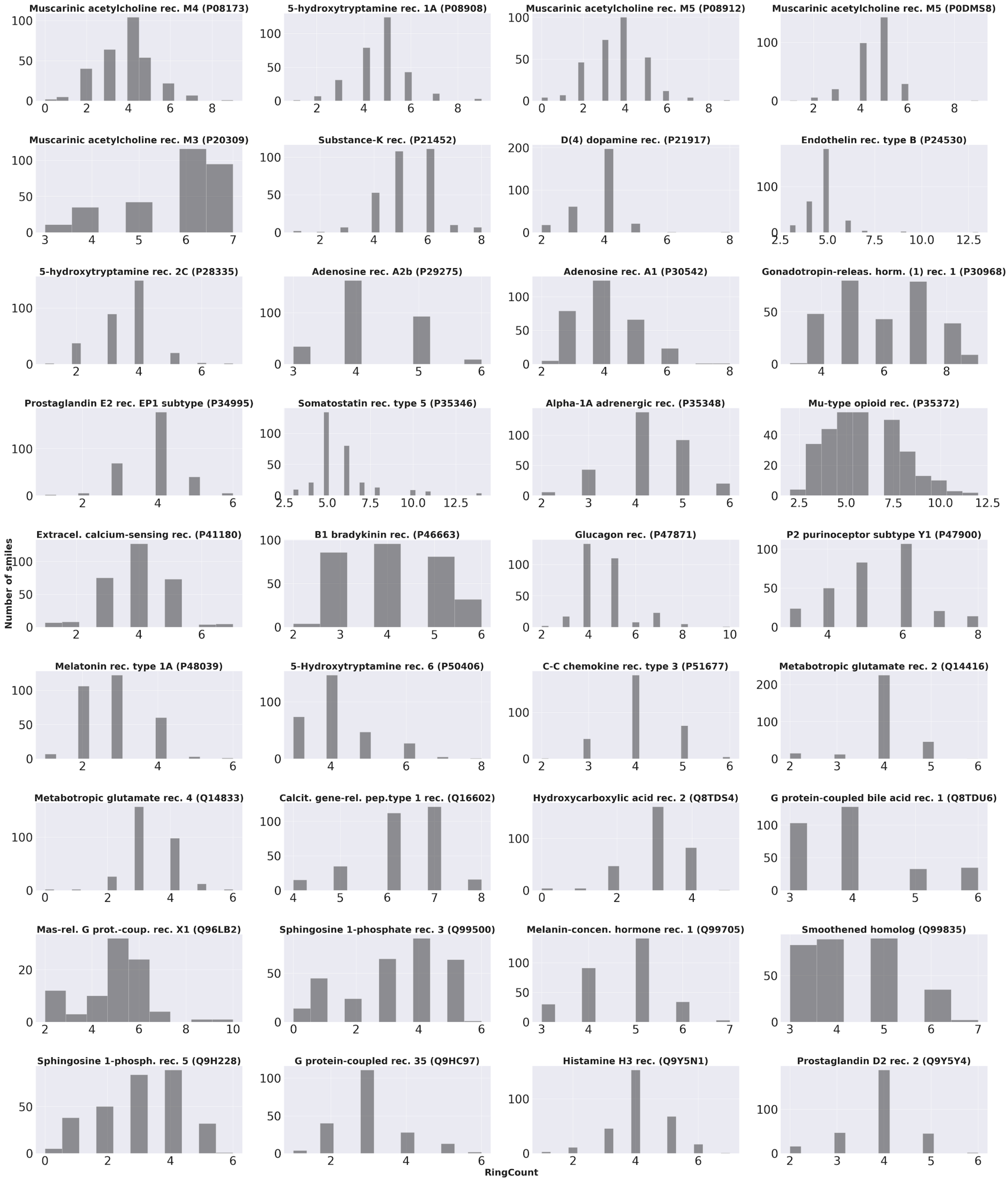

Figure S8: Potent ligands - Histograms considering number of rings count distribution for all datasets.

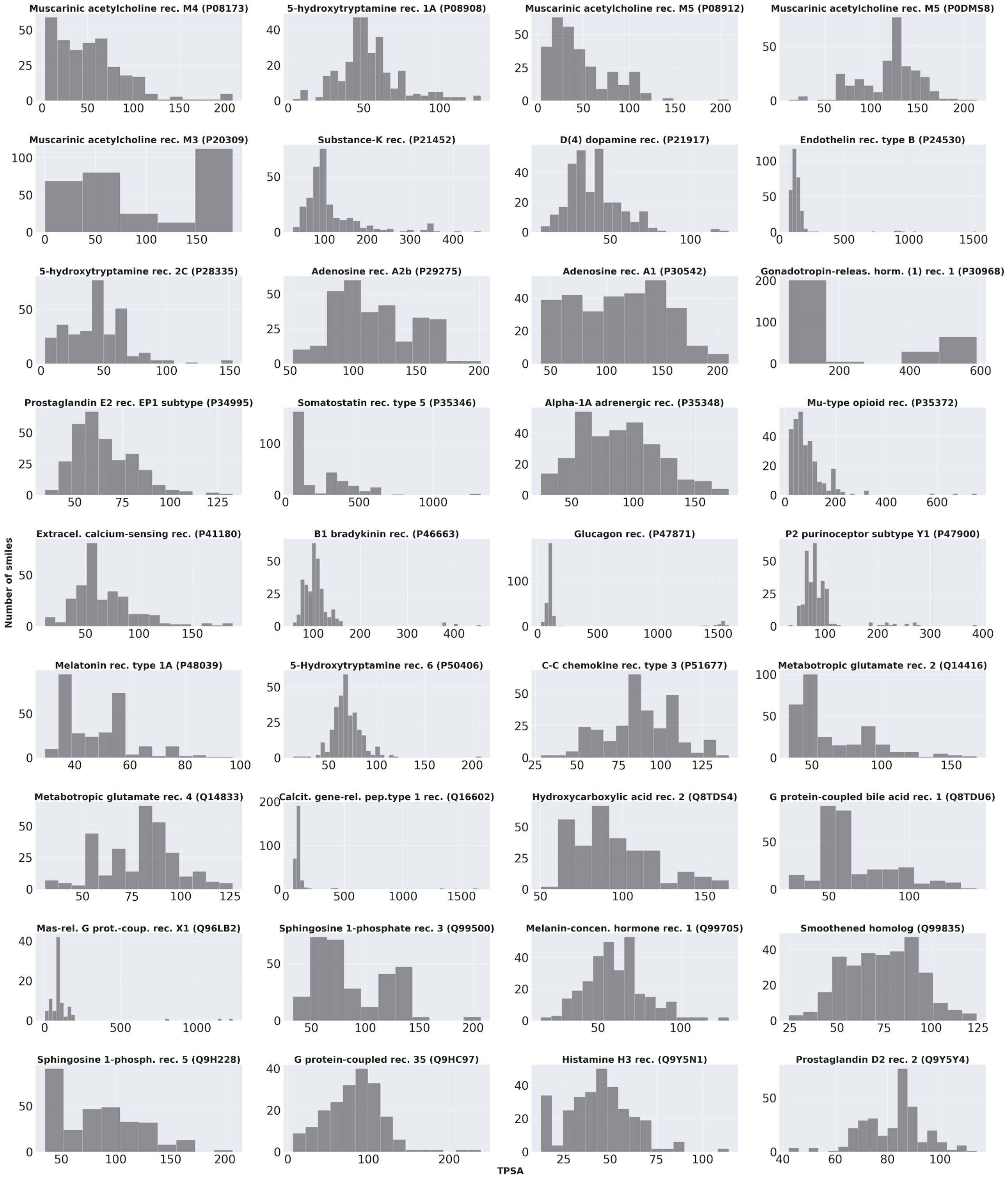

Figure S9: Potent ligands - Histograms considering topological polar surface distribution for all datasets.

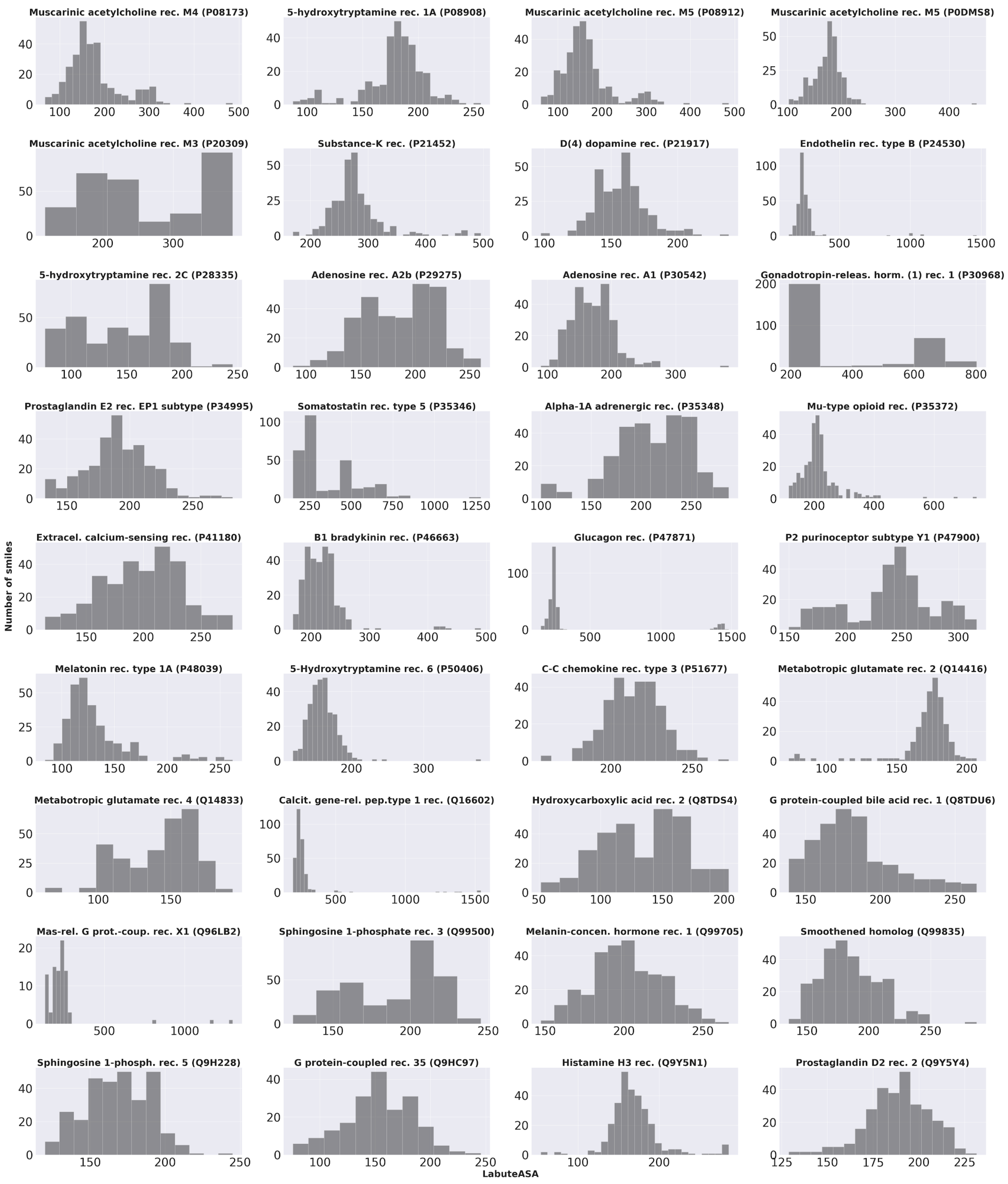

Figure S10: Potent ligands - Histograms considering Labute's Approximate Surface Area distribution for all datasets.

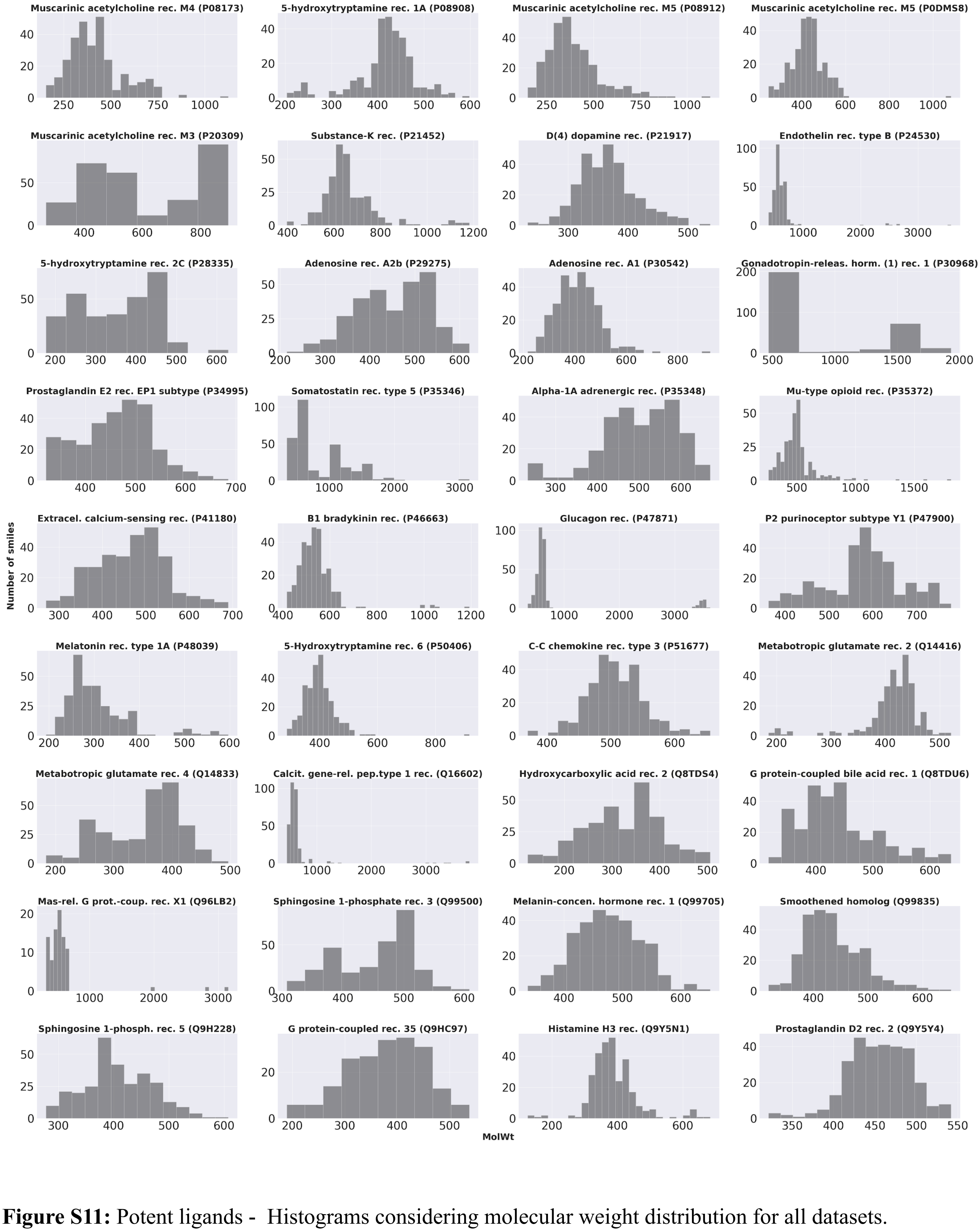

Figure S11: Potent ligands - Histograms considering molecular weight distribution for all datasets.

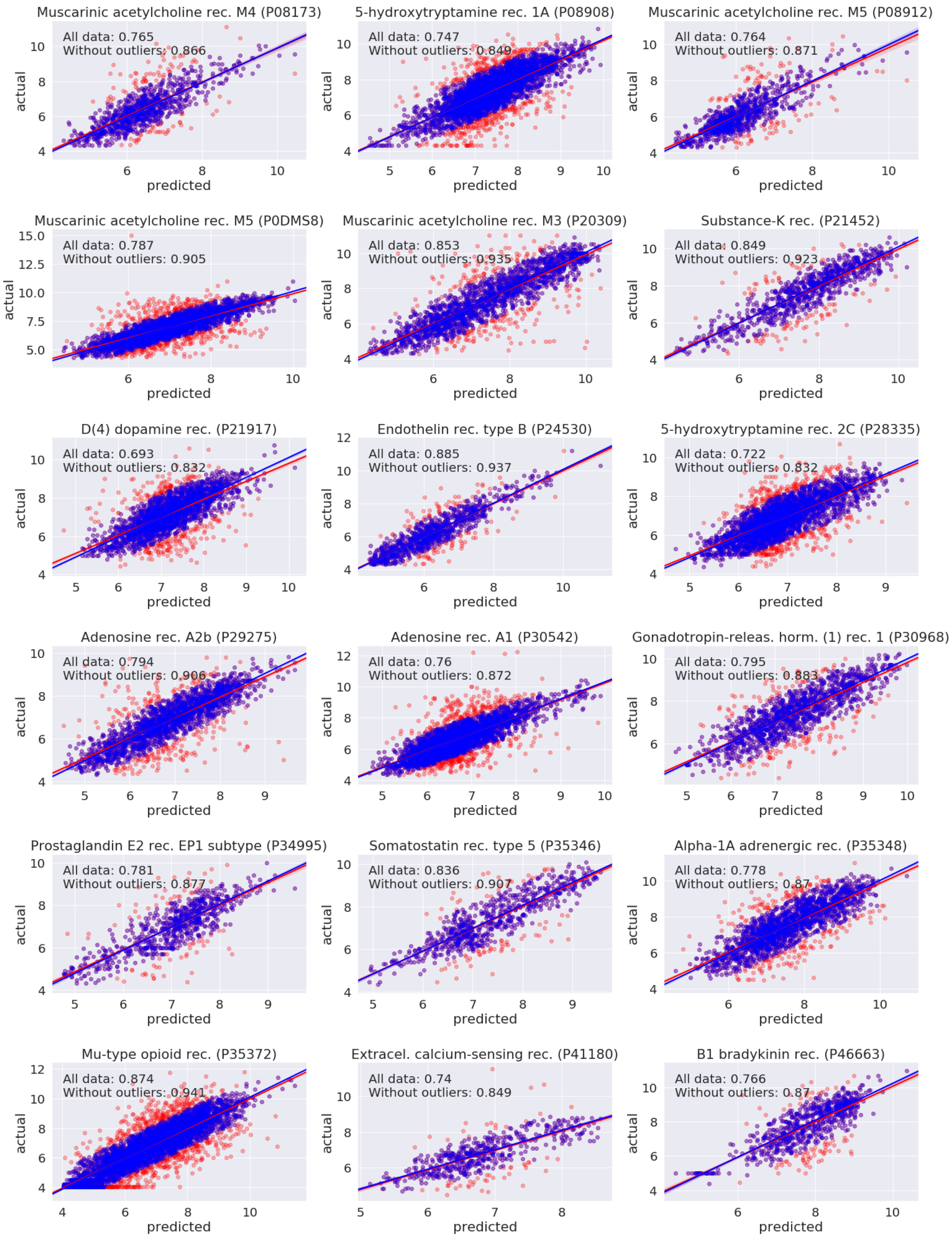

Figure S12: Scatter plots, part 1 - Regression analysis considering cross-validation schemes. Pearson's correlation coefficients are also shown in the top-left corner. The graphs show the correlation between experimental and predicted values.

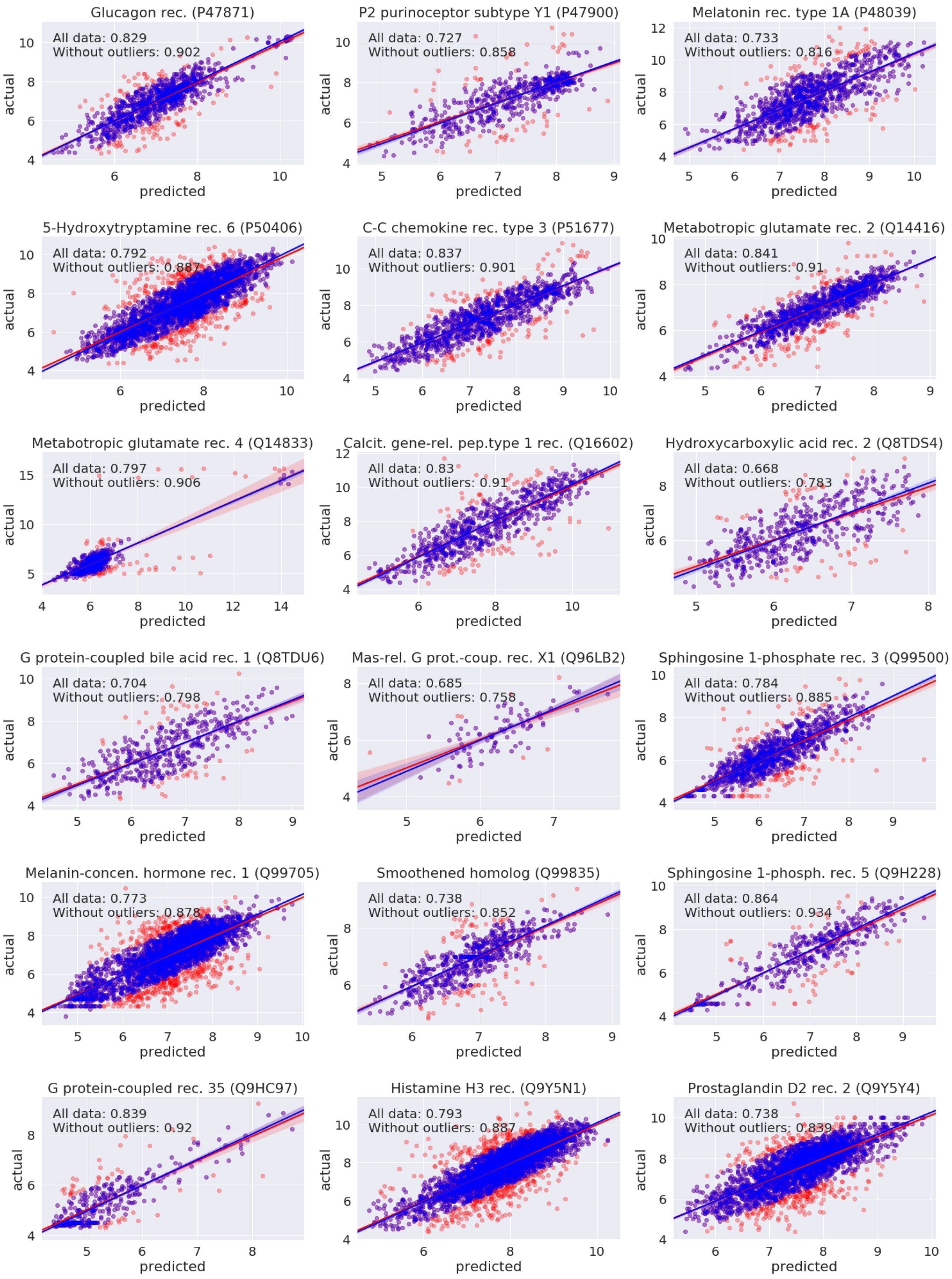

Figure S13: Scatter plots, part 2 - Regression analysis considering cross-validation schemes. Pearson's correlation coefficients are also shown in the top-left corner. The graphs show the correlation between experimental and predicted values.

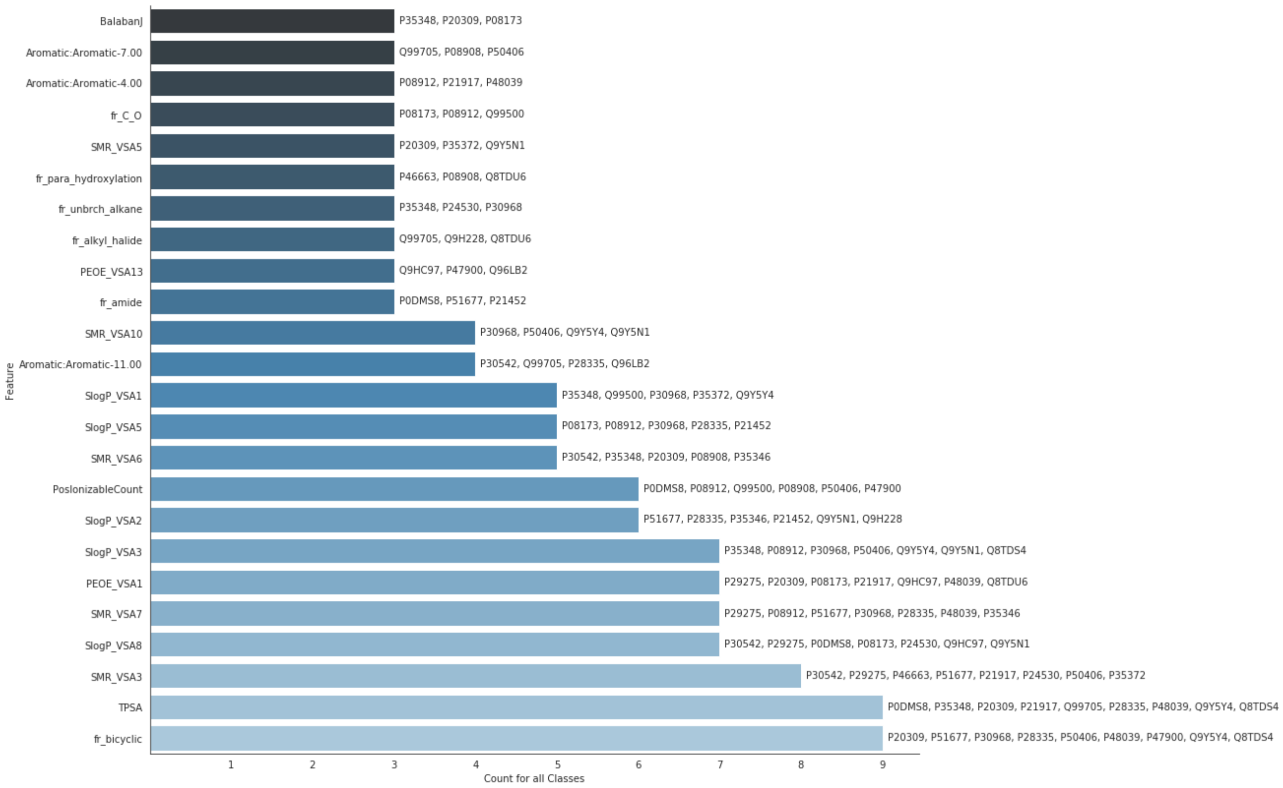

Figure S14: Shows the distribution of the top ten features selected via forward Greedy approach for Class A only receptors.

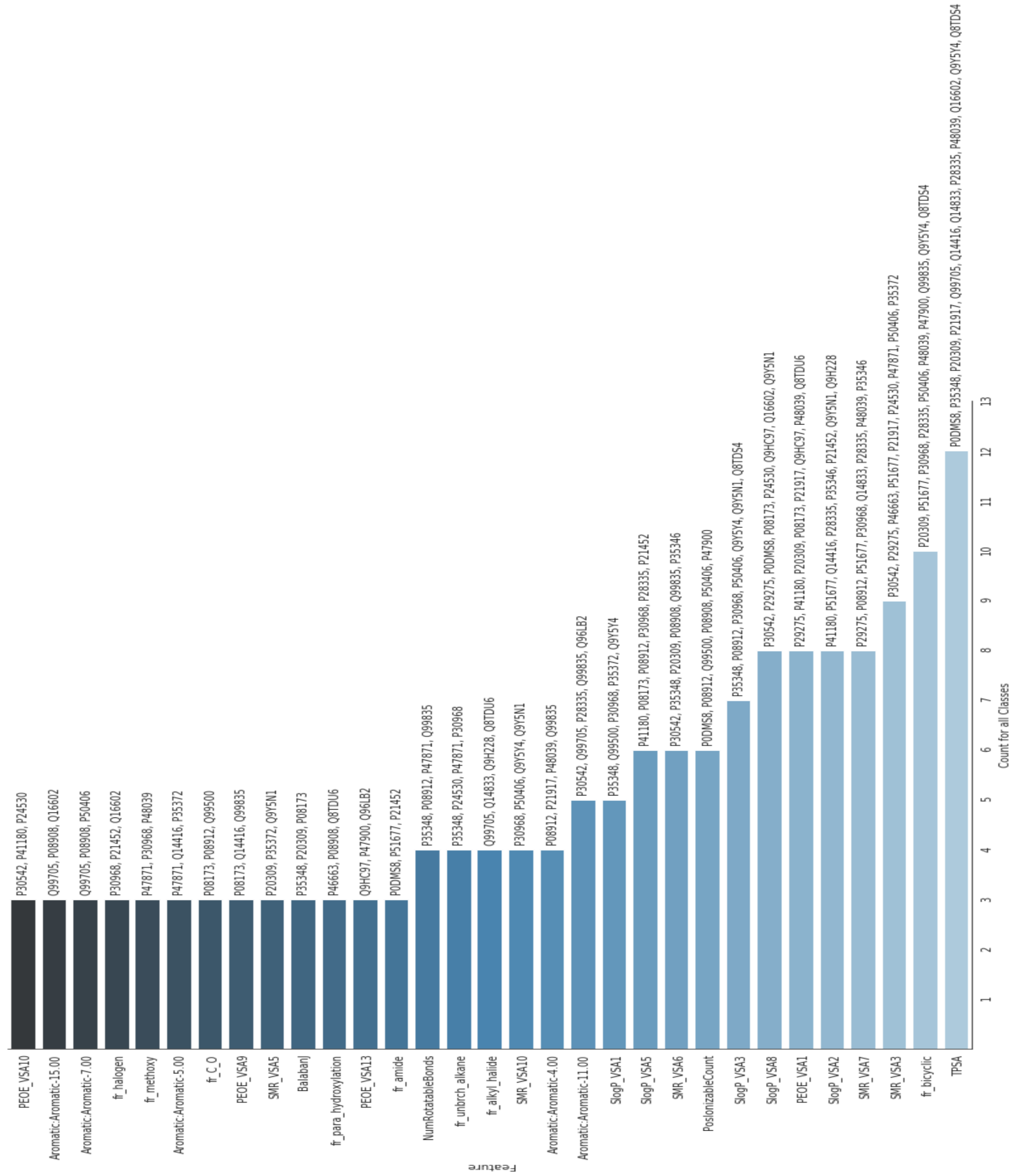

Figure S15: Shows the distribution of the top ten features selected via forward Greedy approach for all receptors.

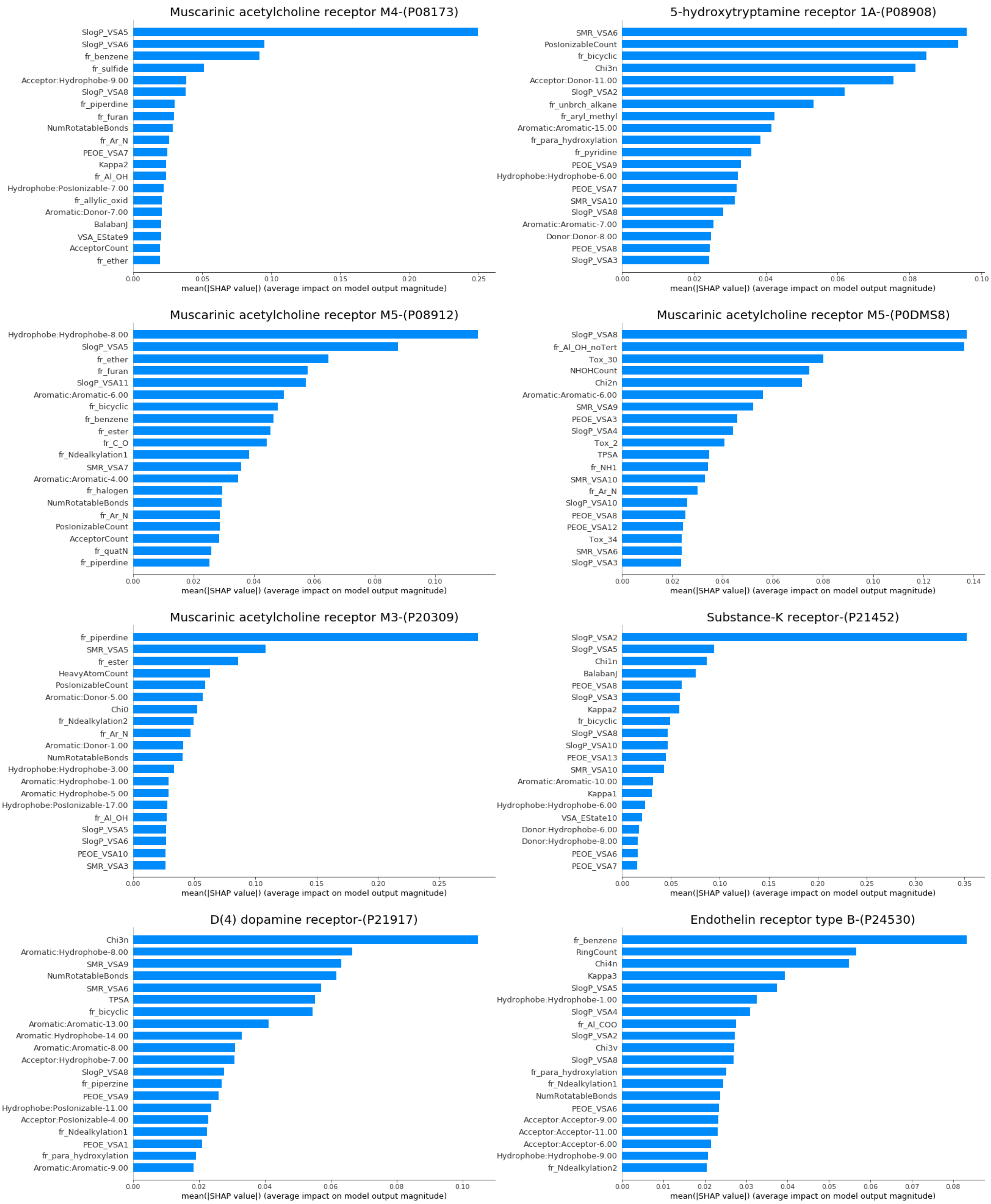

Figure S16: SHAP bar plots - Feature importance plots, the bars represent how strongly different input features affect the output of the respective model. Features are listed in descending order of importance.

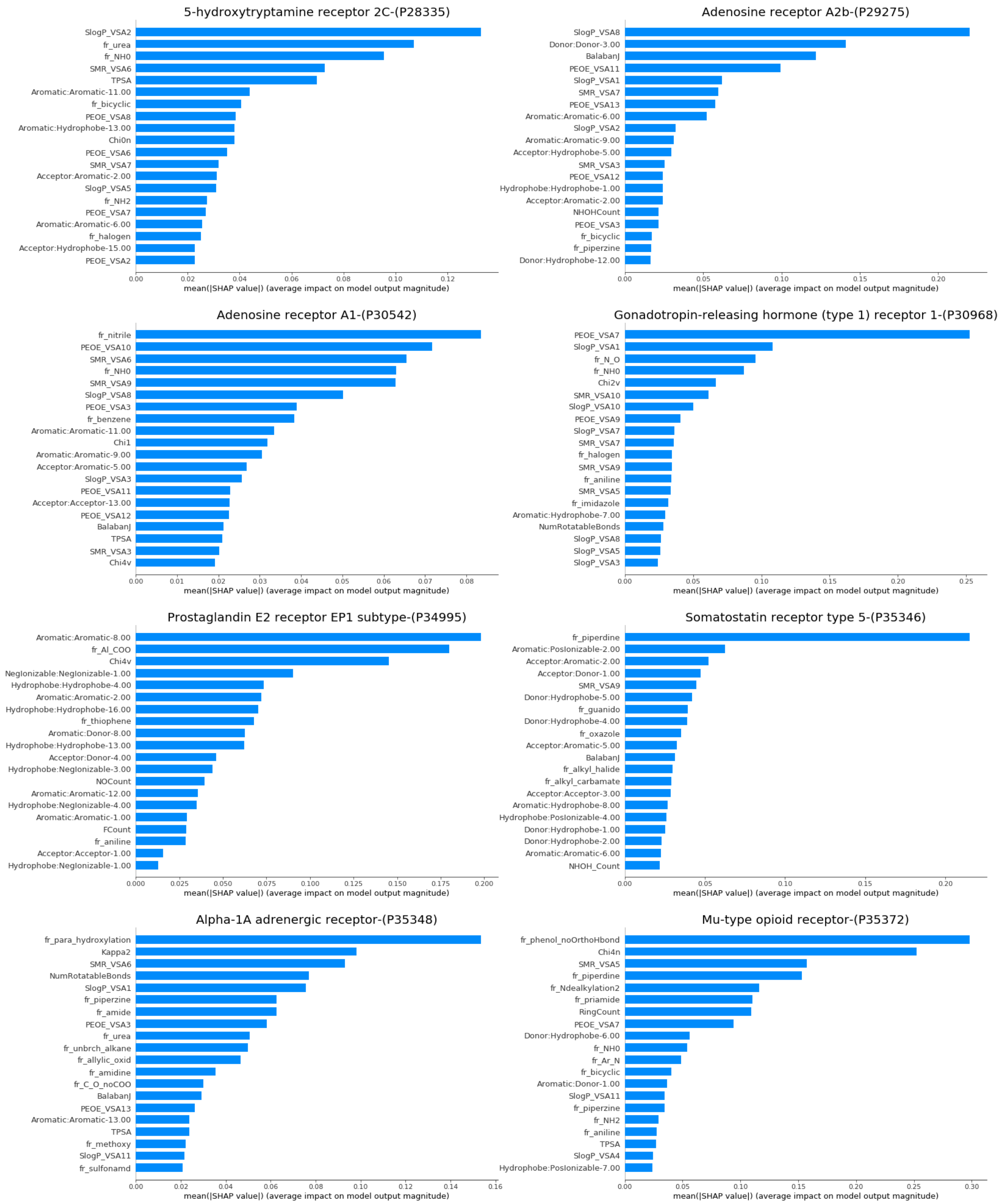

Figure S17: SHAP bar plots - Feature importance plots, the bars represent how strongly different input features affect the output of the respective model. Features are listed in descending order of importance.

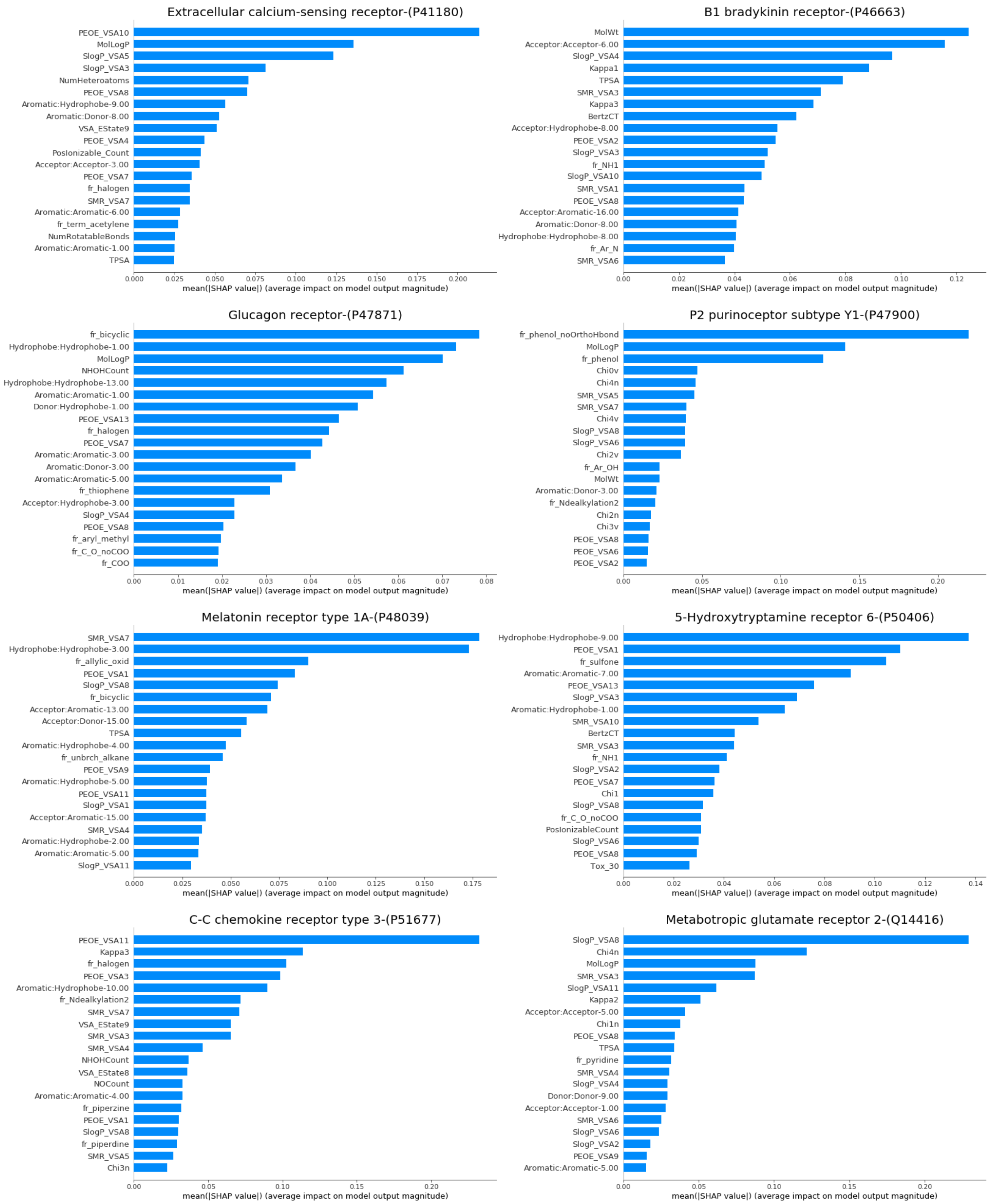

Figure S18: SHAP bar plots - Feature importance plots, the bars represent how strongly different input features affect the output of the respective model. Features are listed in descending order of importance.

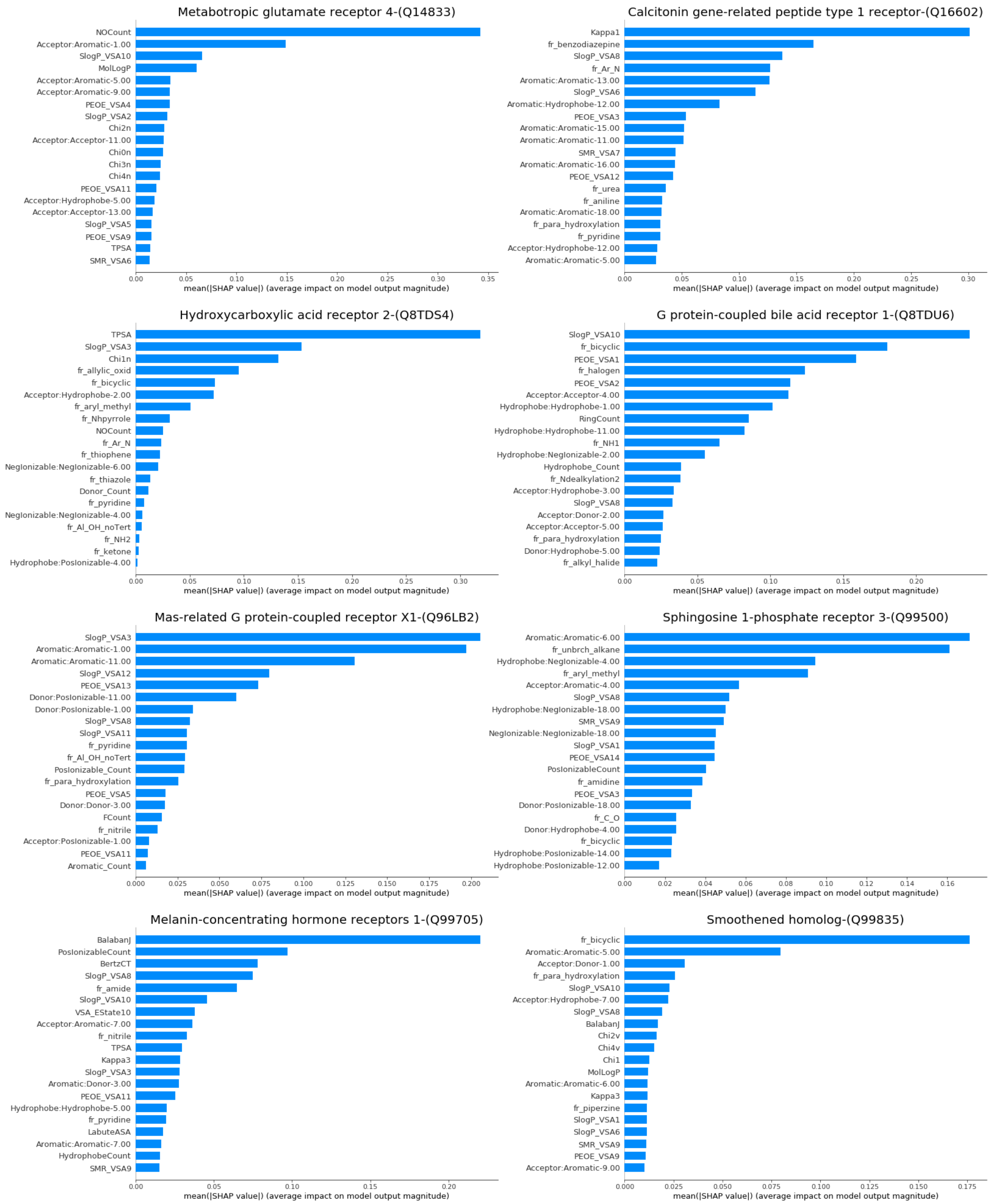

Figure S19: SHAP bar plots - Feature importance plots, the bars represent how strongly different input features affect the output of the respective model. Features are listed in descending order of importance.

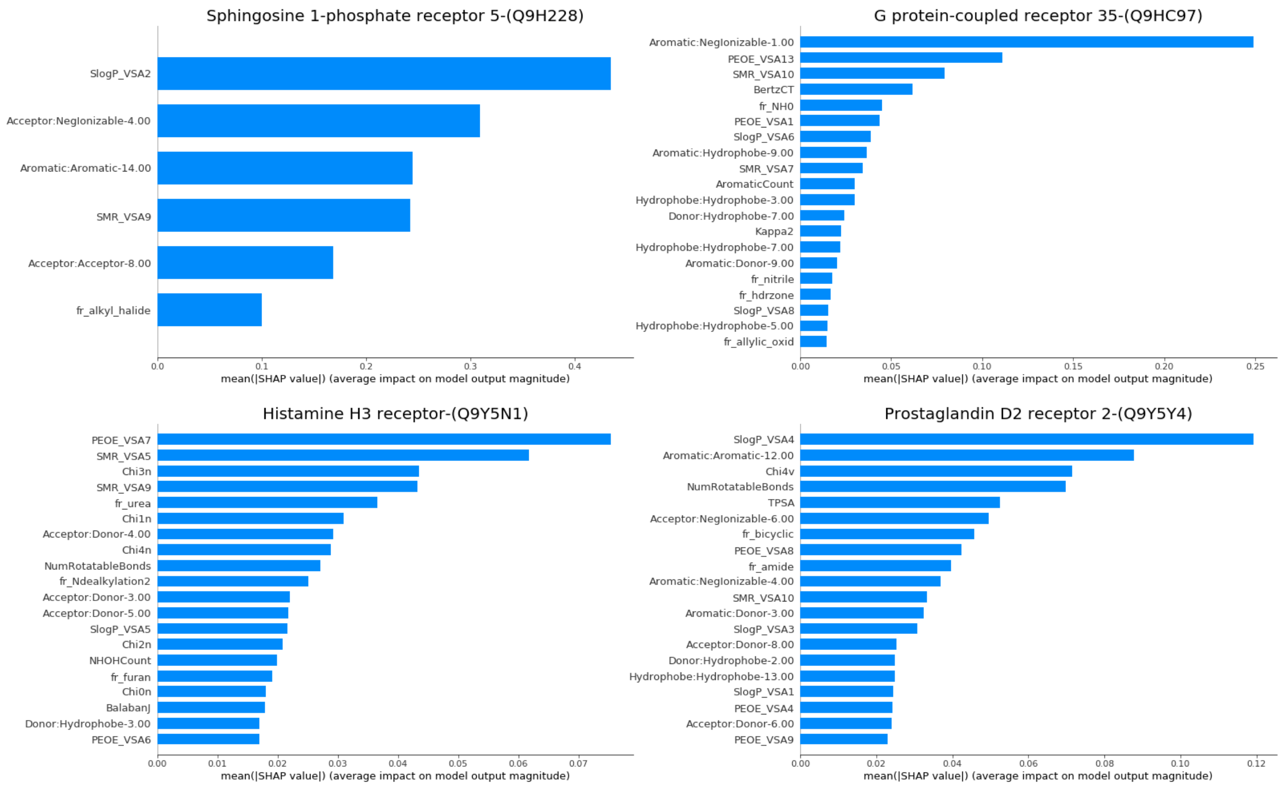

Figure S20: SHAP bar plots - Feature importance plots, the bars represent how strongly different input features affect the output of the respective model. Features are listed in descending order of importance.

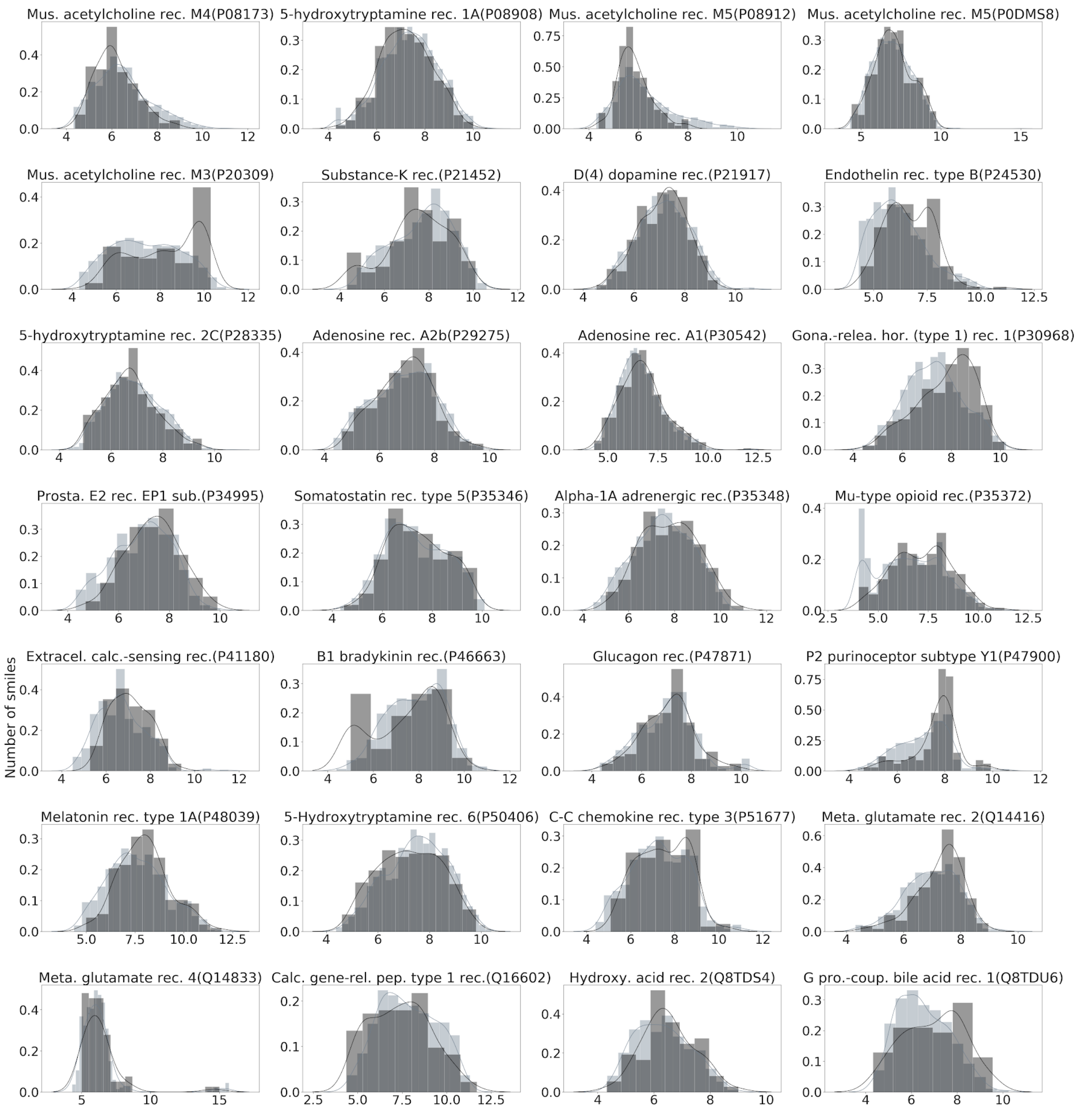

Figure S21: Histograms considering molecular activity distribution for training and low-redundancy independent blind tests datasets. The histogram in light grey color represents training and the histogram in dark grey color represents testing datasets.

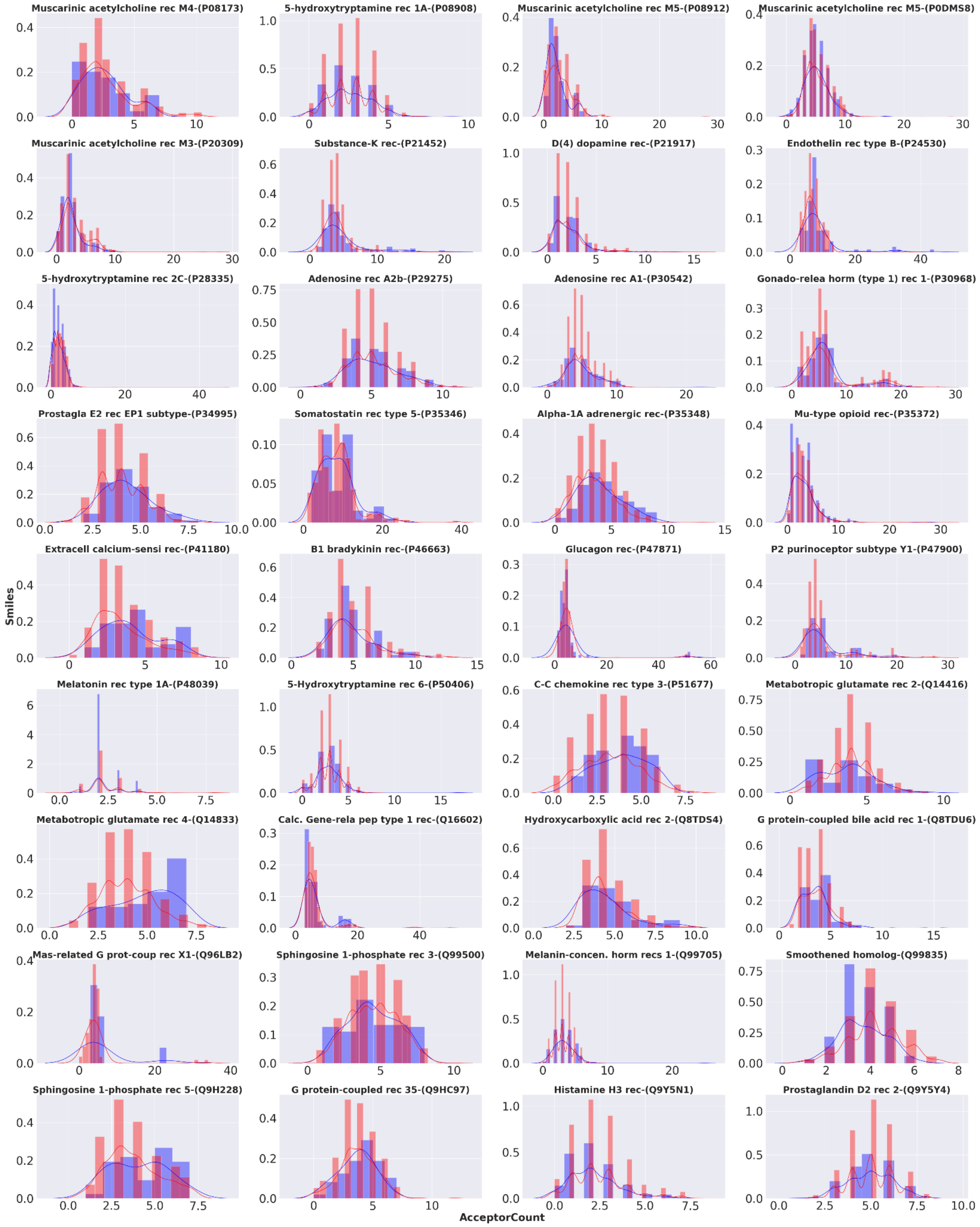

Figure S22: Histograms considering count of hydrogen bonds acceptor distribution for datasets without outliers (from cross-validation schemes) in red and considering only outliers in blue.

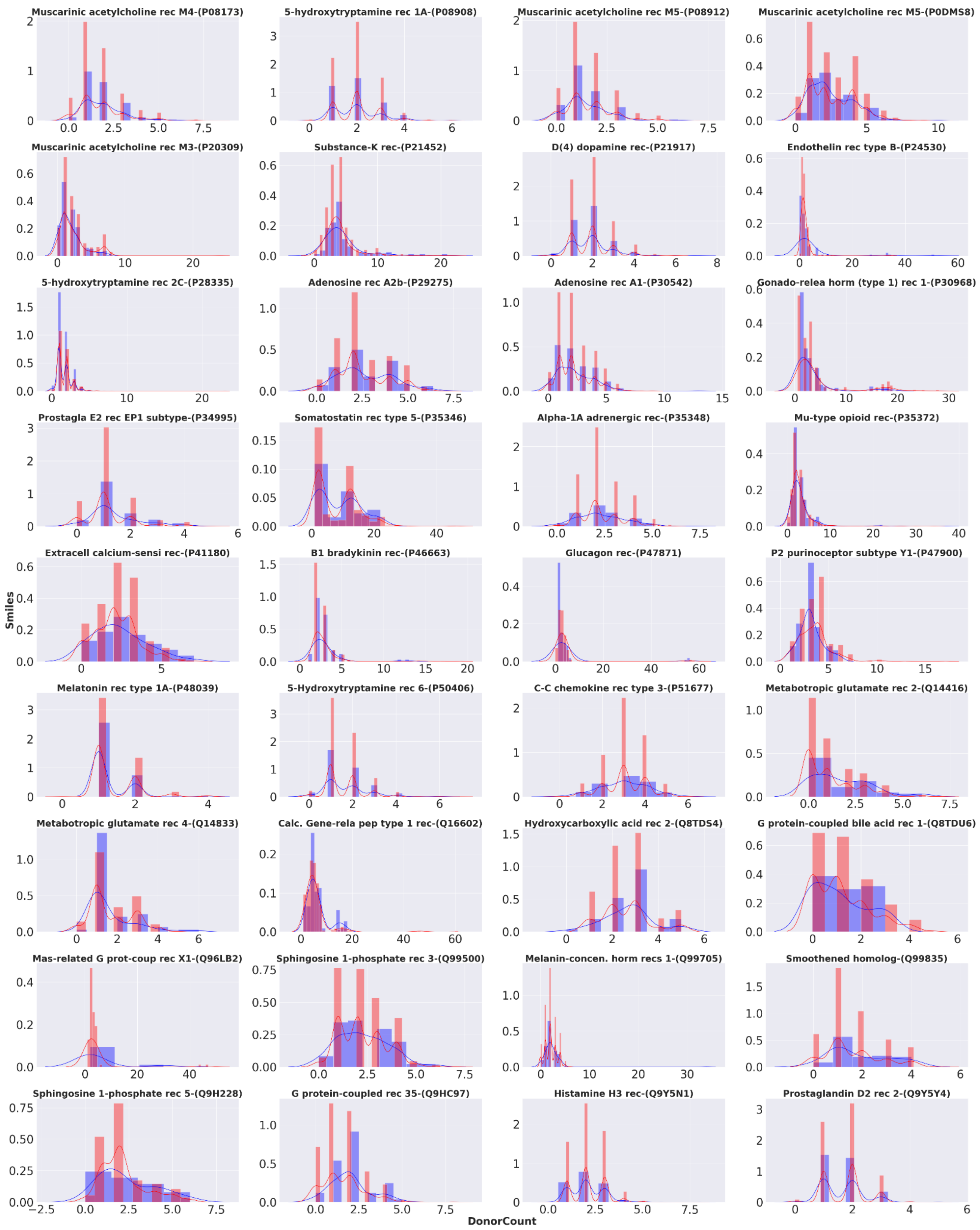

Figure S23: Histograms considering count of hydrogen bonds donor distribution for datasets without outliers (from cross-validation schemes) in red and considering only outliers in blue.

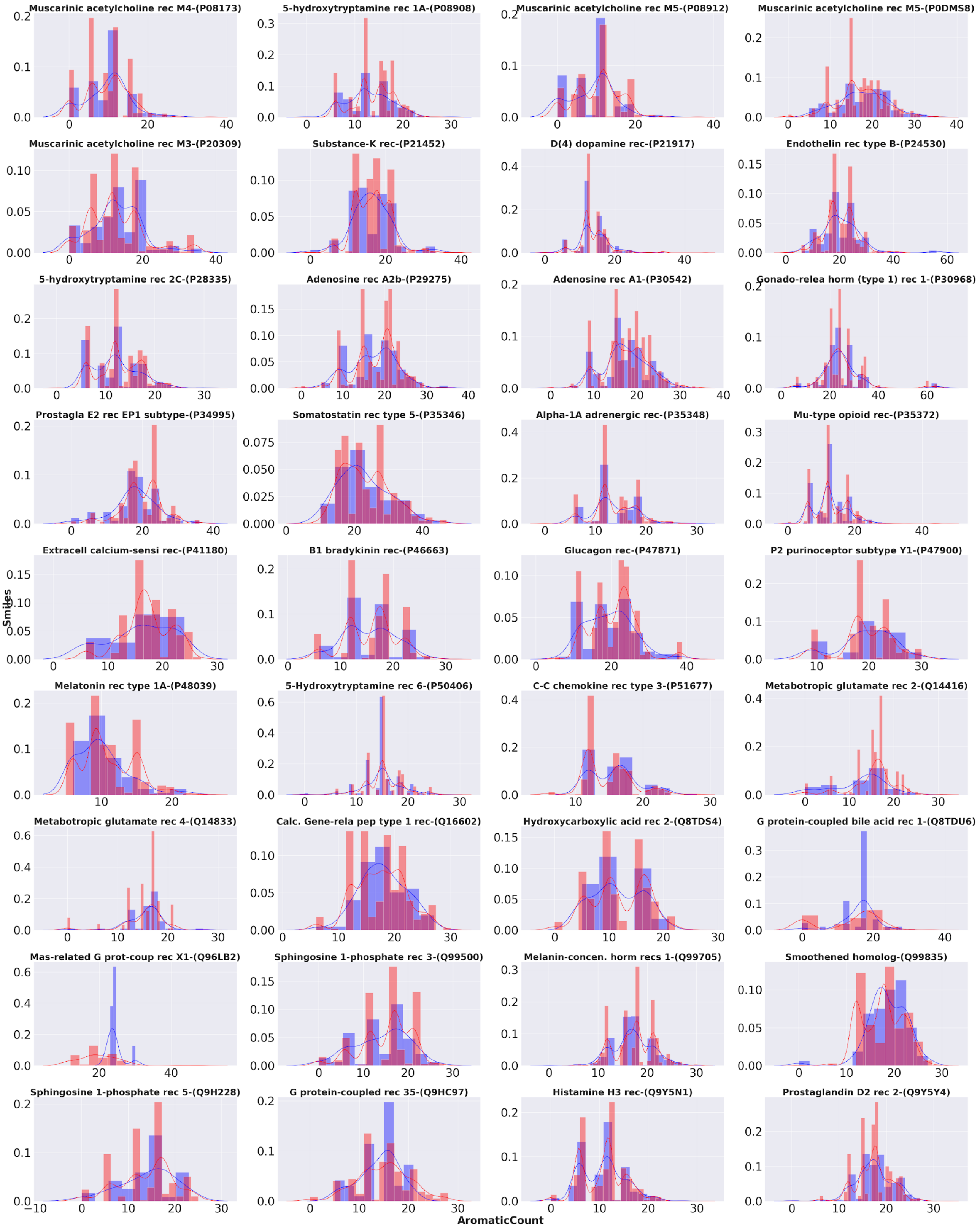

Figure S24: Histograms considering aromaticity distribution for datasets without outliers (from cross-validation schemes) in red and considering only outliers in blue.

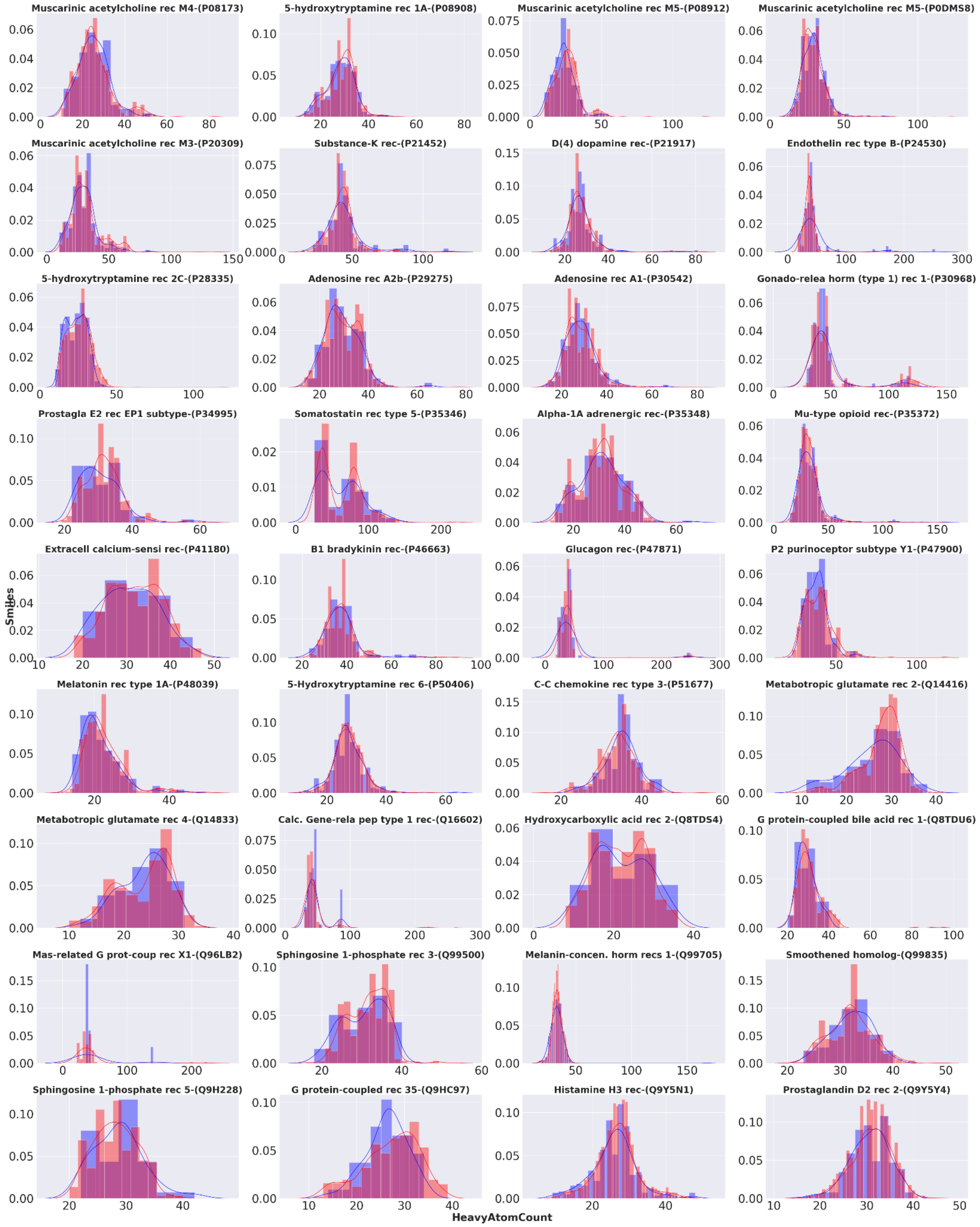

Figure S25: Histograms considering count of heavy atoms distribution for datasets without outliers (from cross-validation schemes) in red and considering only outliers in blue.

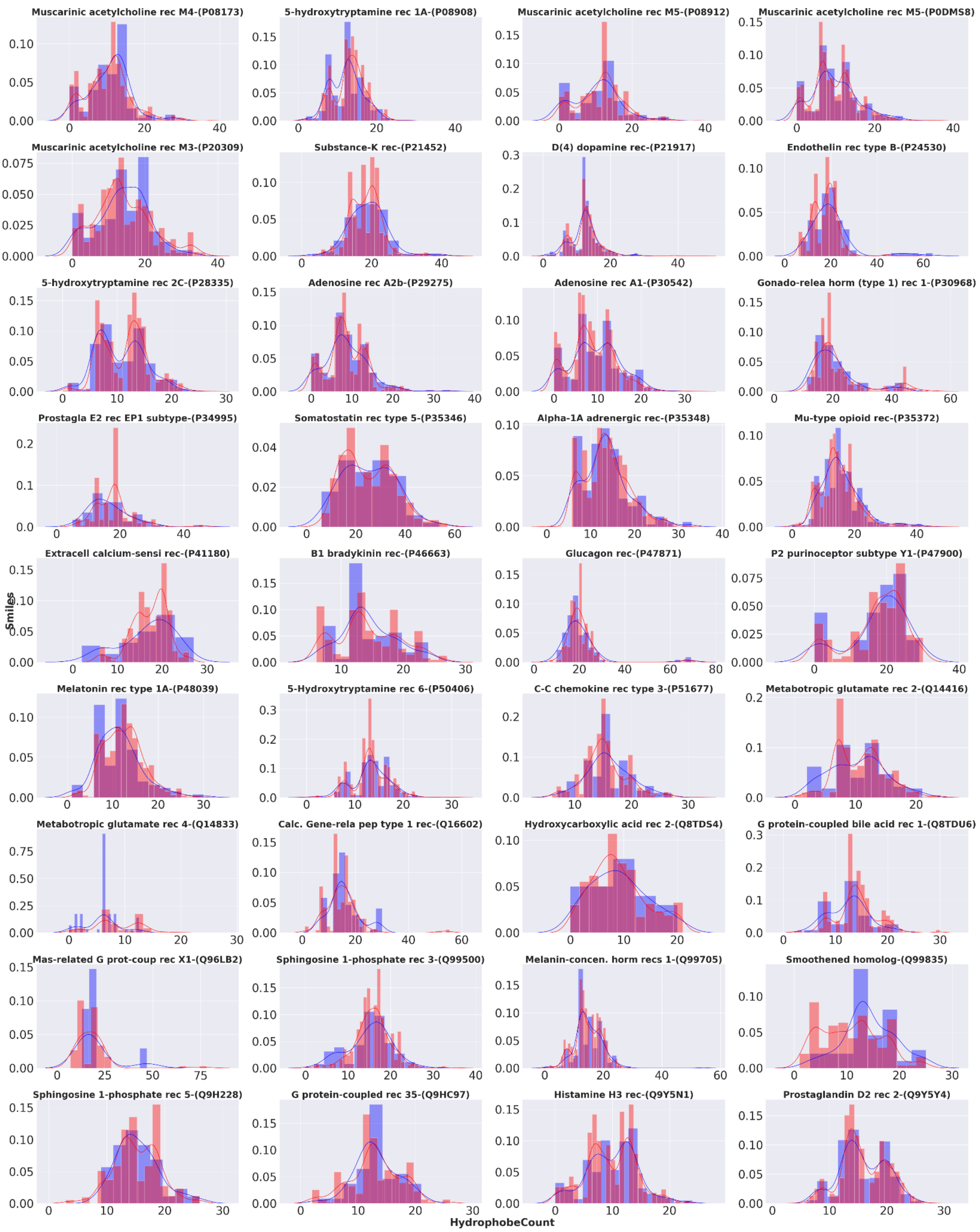

Figure S26: Histograms considering hydrophobicity distribution for datasets without outliers (from cross-validation schemes) in red and considering only outliers in blue.

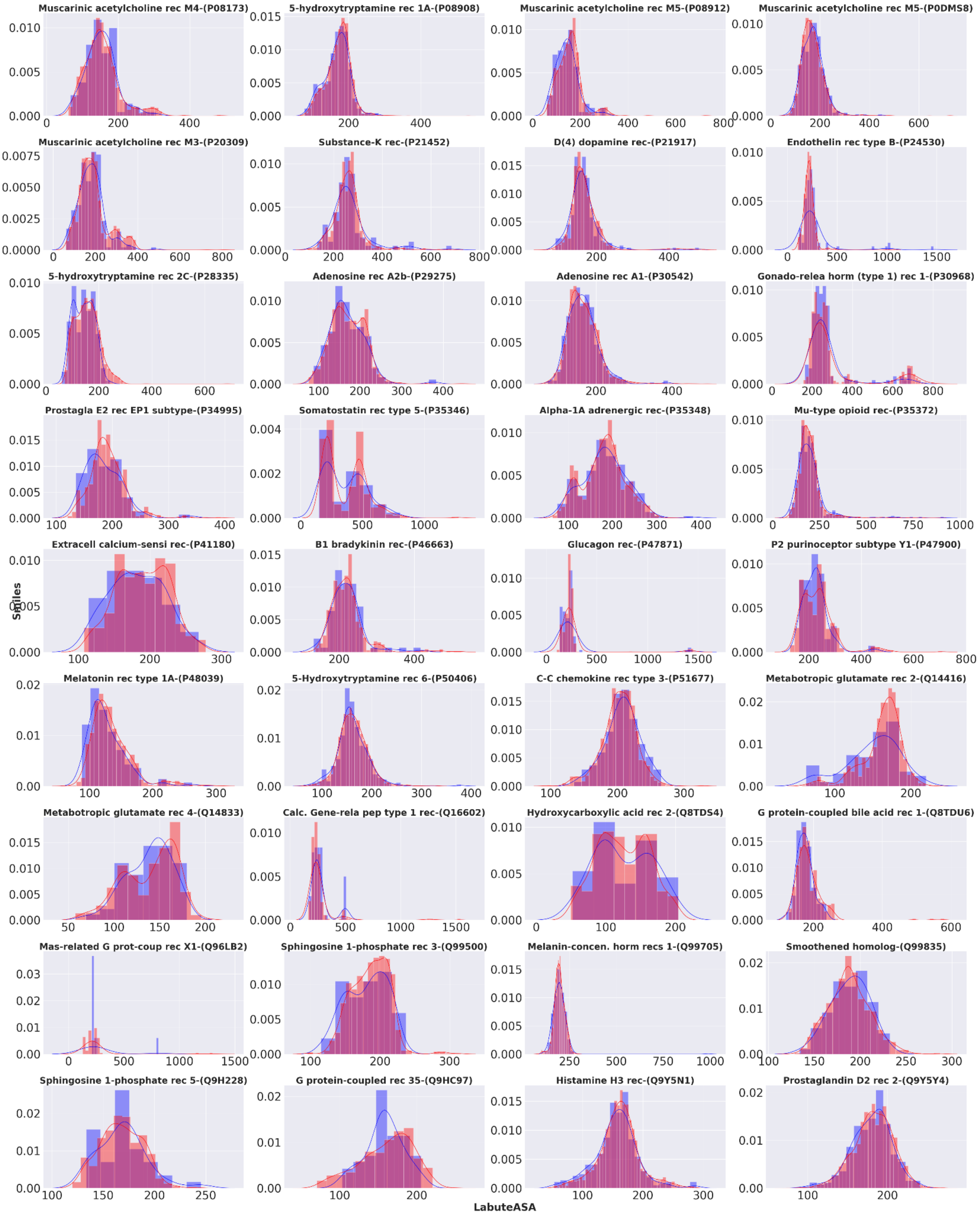

Figure S27: Histograms considering Labute's Approximate Surface Area distribution for datasets without outliers (from cross-validation schemes) in red and considering only outliers in blue.

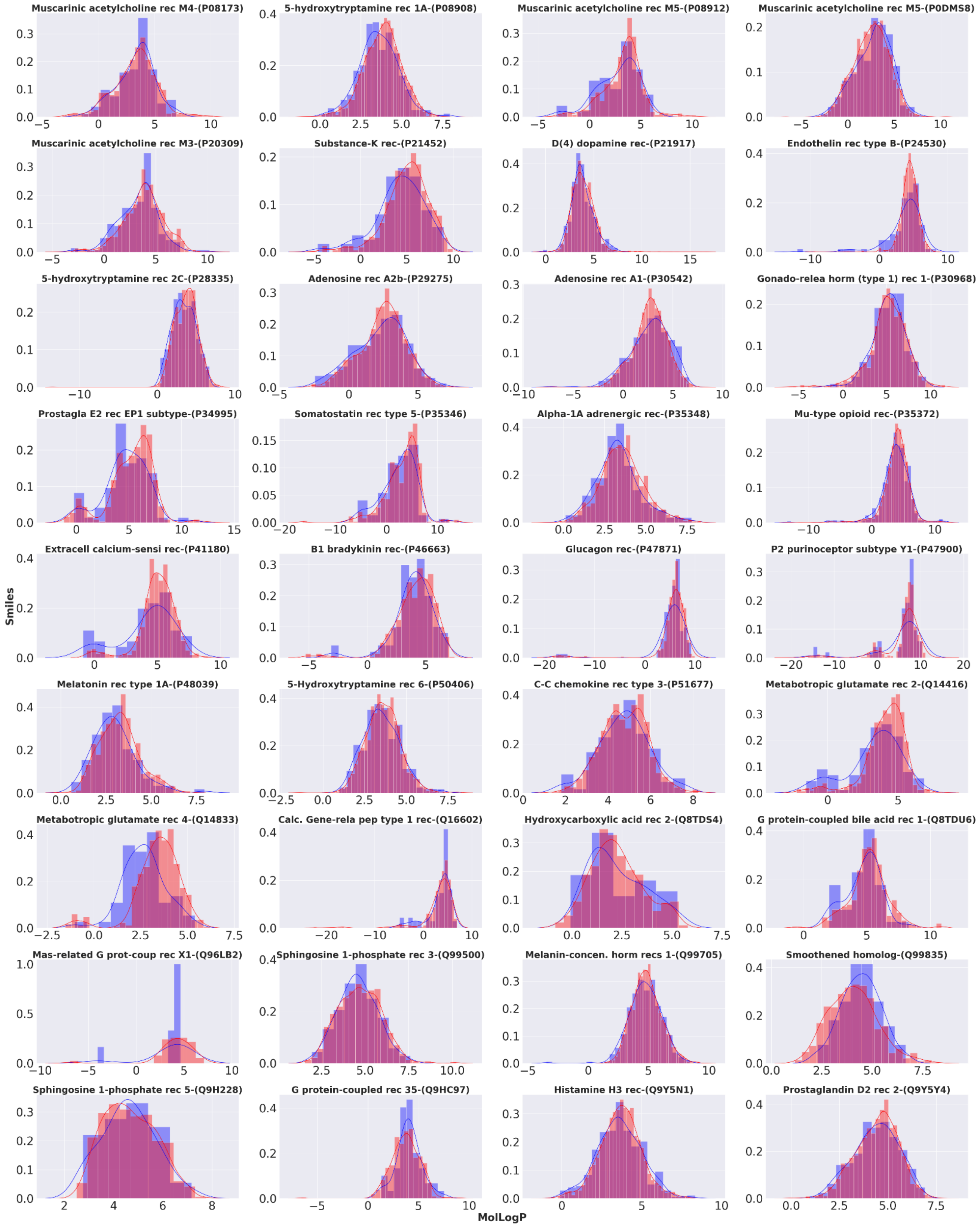

Figure S28: Histograms considering log *P* distribution for datasets without outliers (from cross-validation schemes) in red and considering only outliers in blue.

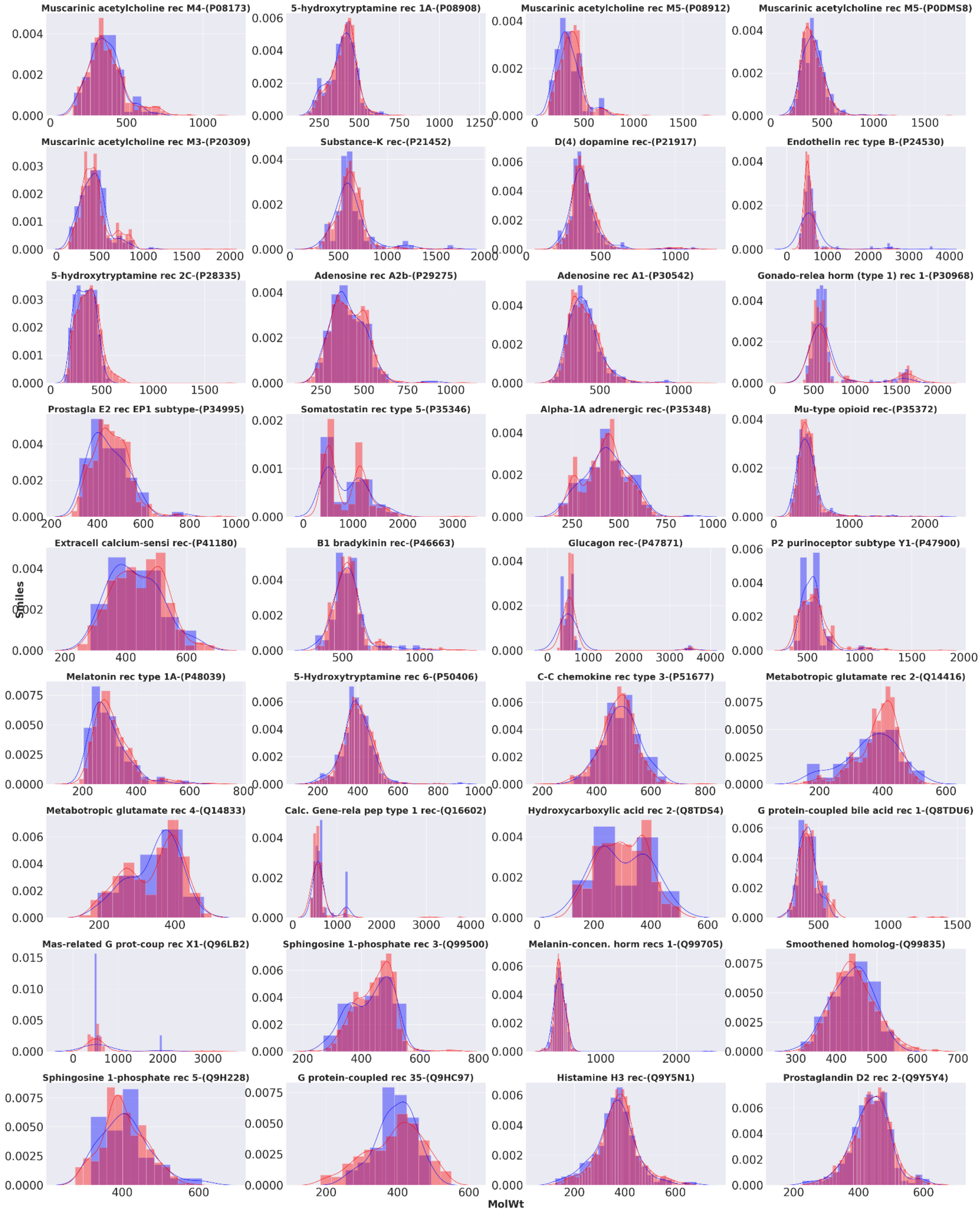

Figure S29: Histograms considering molecular weight distribution for datasets without outliers (from cross-validation schemes) in red and considering only outliers in blue.

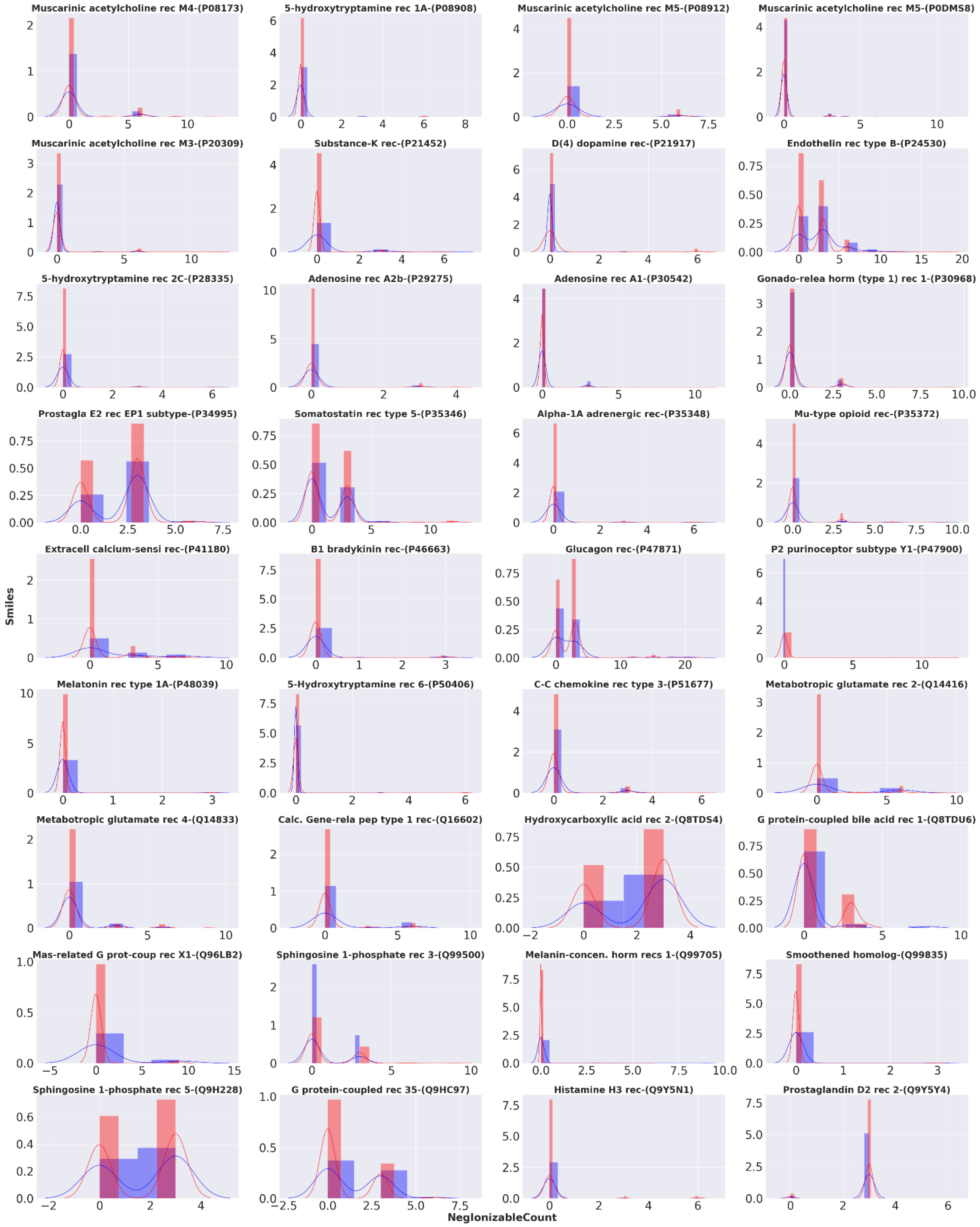

Figure S30: Histograms considering count of negative ionizable atoms for datasets without outliers (from cross-validation schemes) in red and considering only outliers in blue.

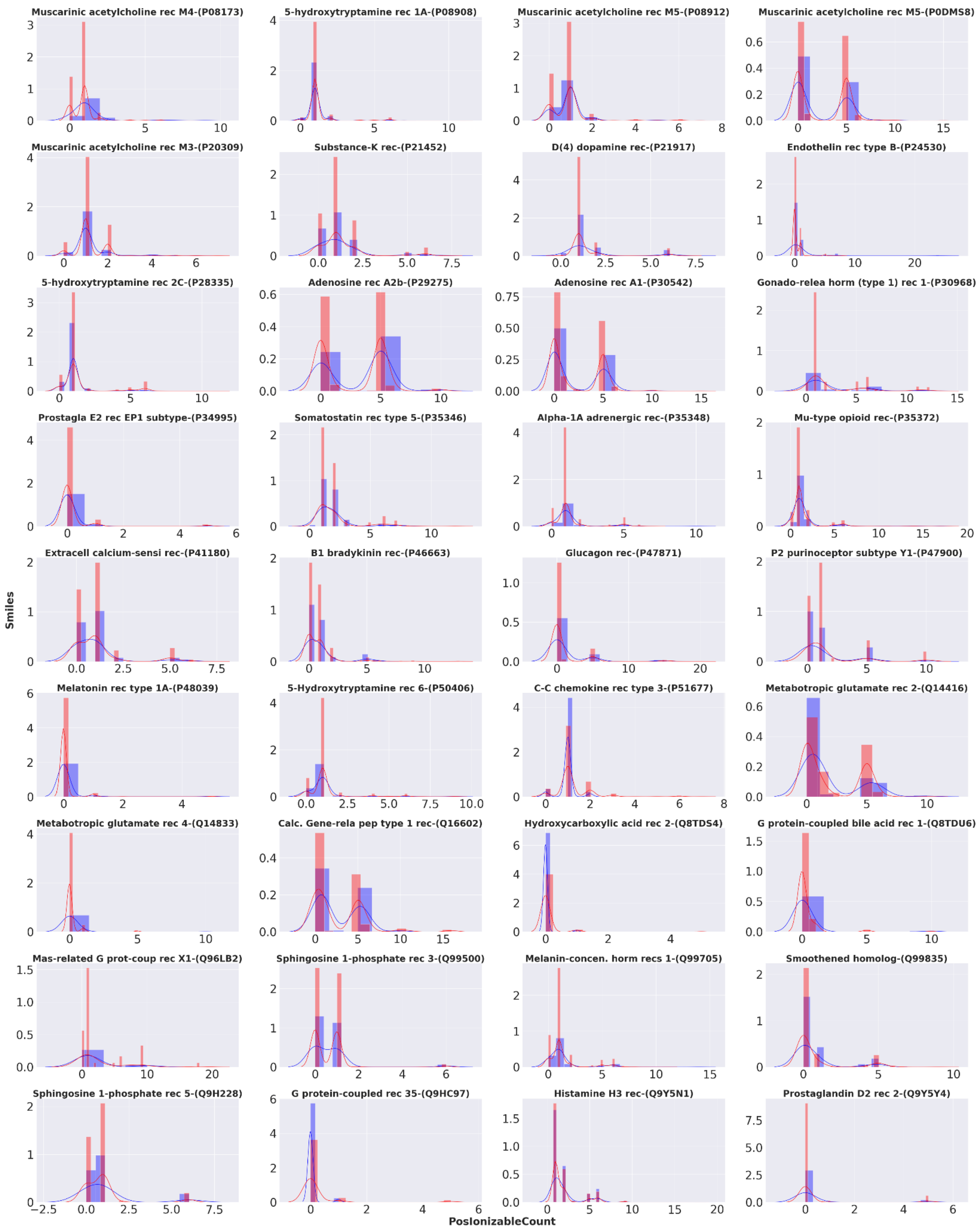

Figure S31: Histograms considering count of positive ionizable atoms for datasets without outliers (from cross-validation schemes) in red and considering only outliers in blue.

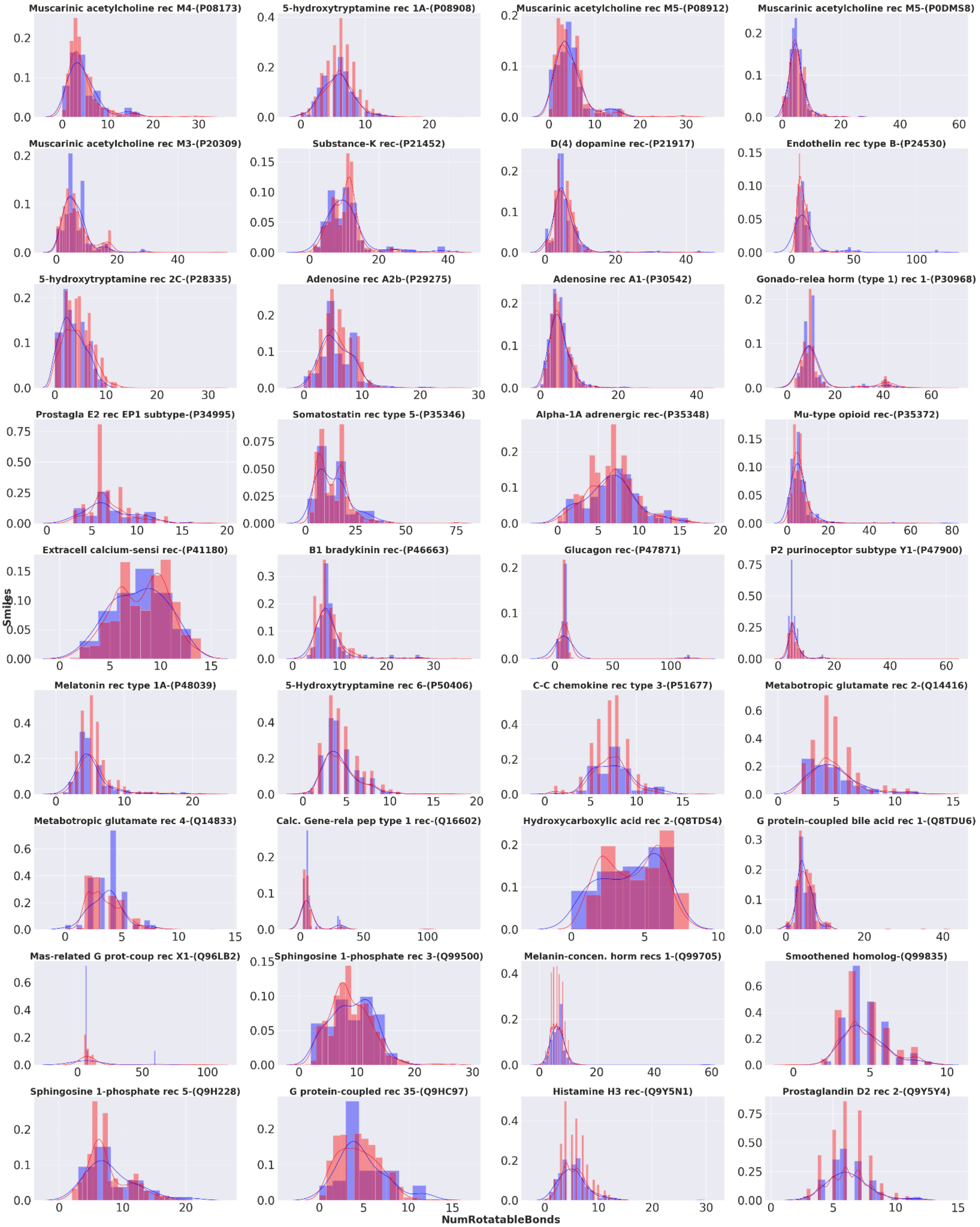

Figure S32: Histograms considering count of Rotatable bonds distribution for datasets without outliers (from cross-validation schemes) in red and considering only outliers in blue.

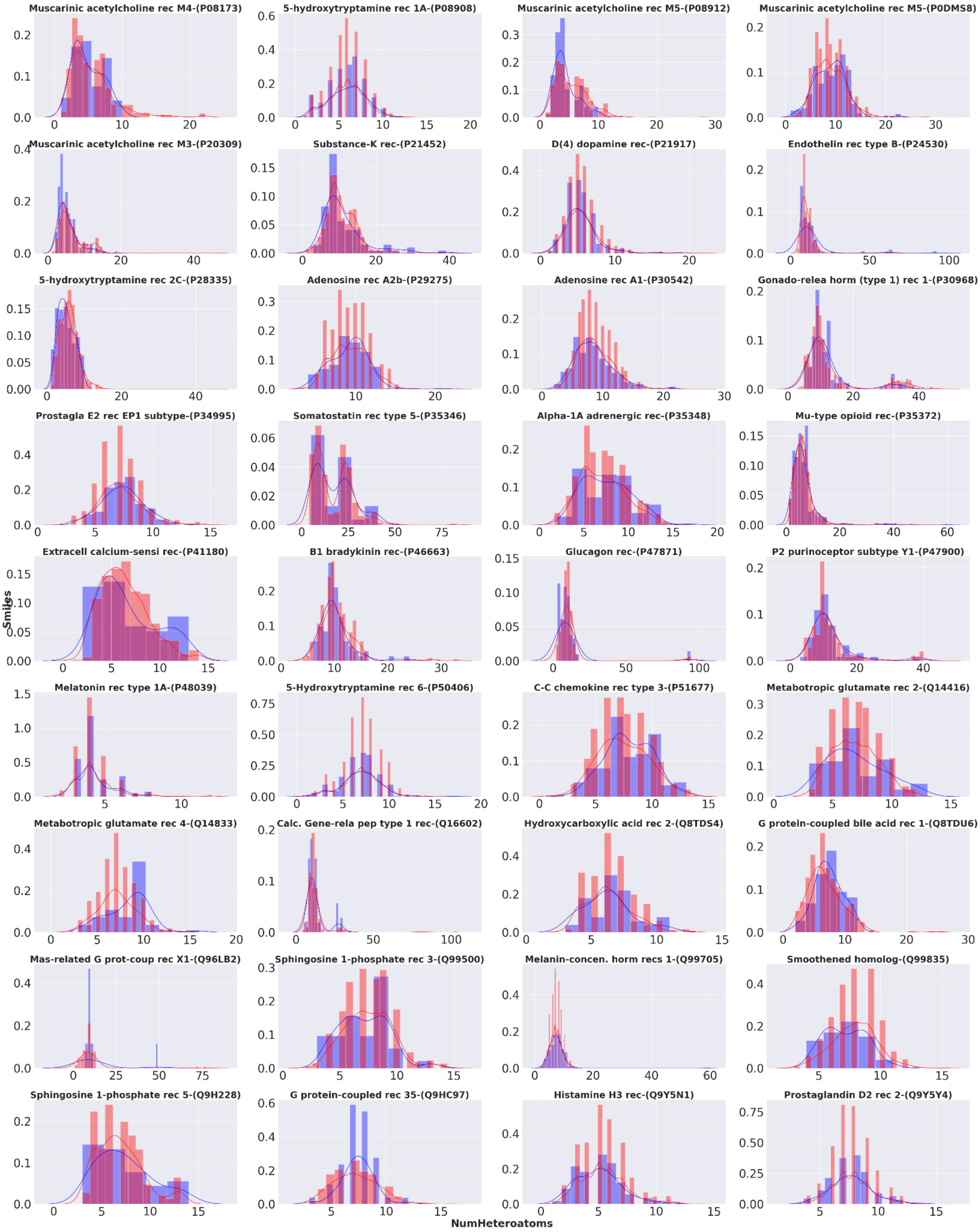

Figure S33: Histograms considering count of heteroatoms for datasets without outliers (from cross-validation schemes) in red and considering only outliers in blue.

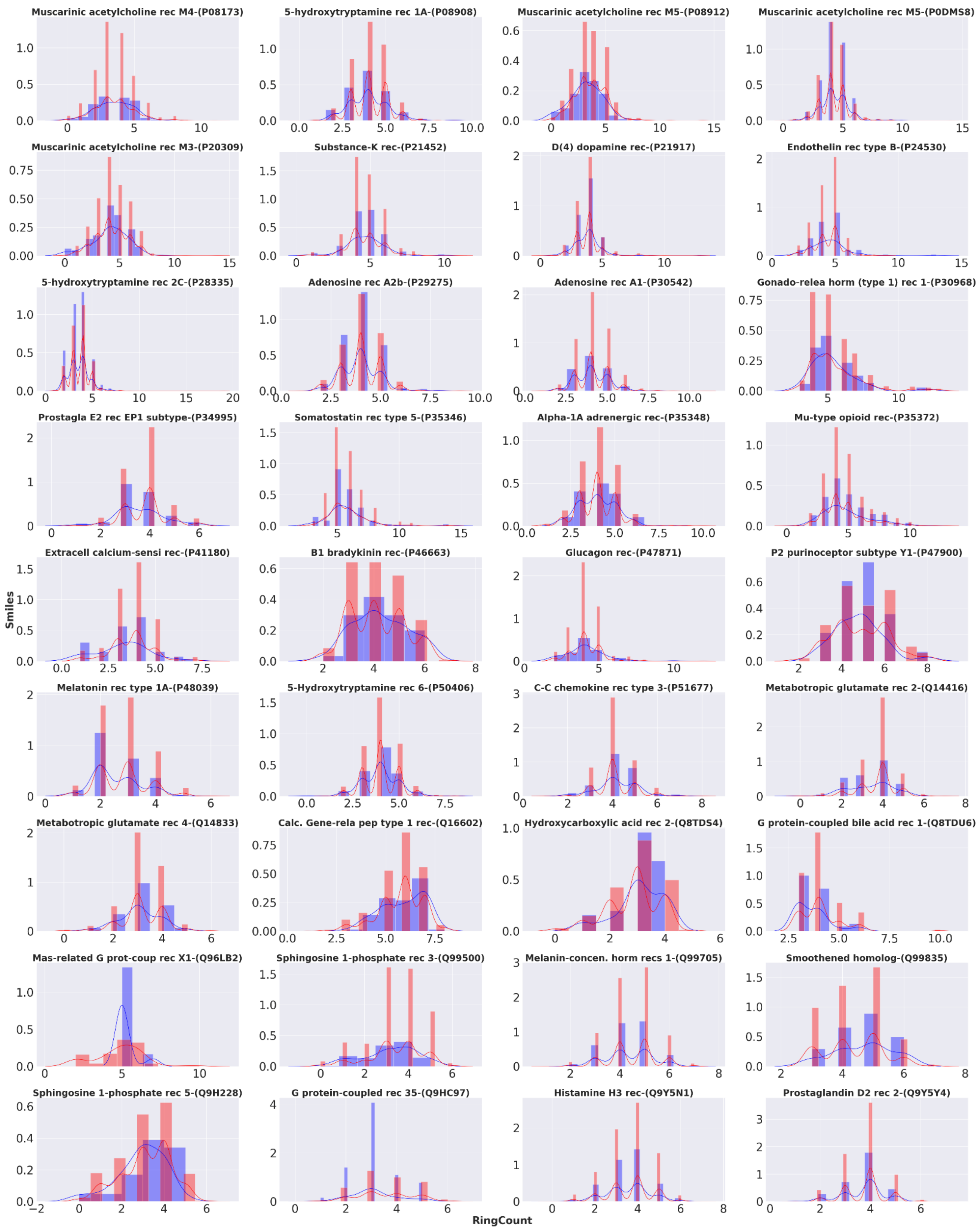

Figure S34: Histograms considering count of rings for datasets without outliers (from cross-validation schemes) in red and considering only outliers in blue.

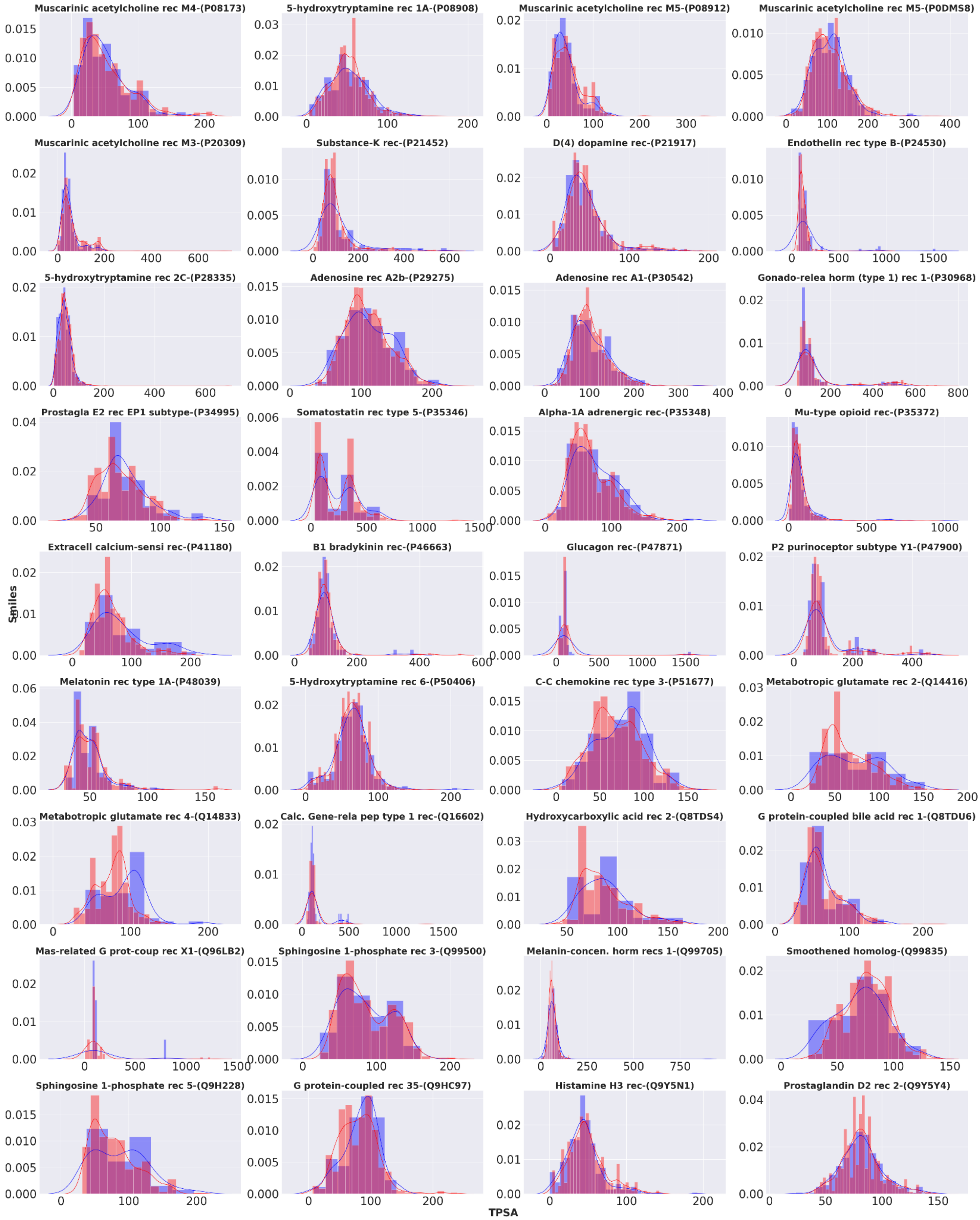

Figure S35: Histograms considering topological polar surface distribution for datasets without outliers (from cross-validation schemes) in red and considering only outliers in blue.

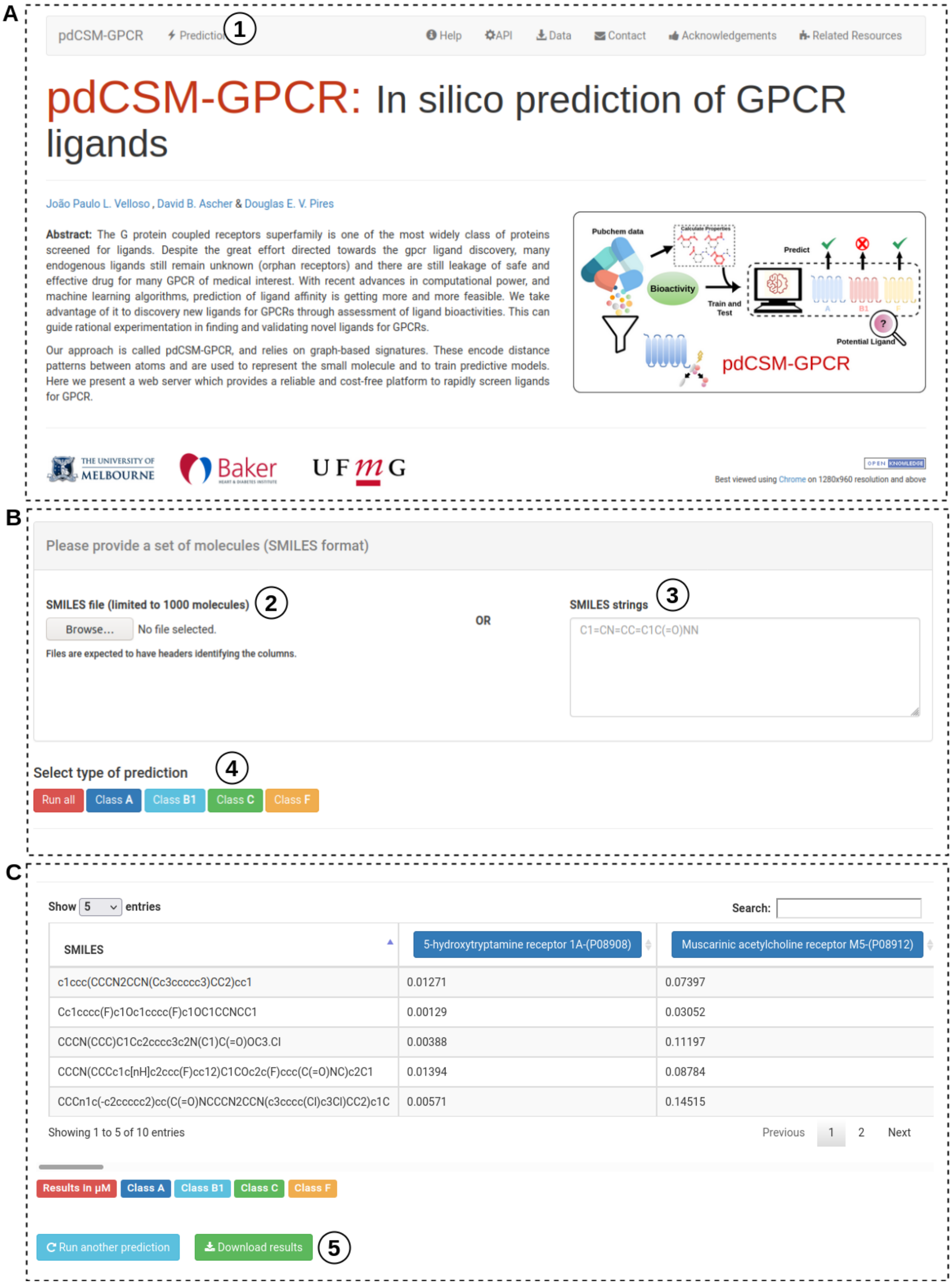

Figure S36: pdCSM-GPCR web server. (A) depicts the landing page for the resource. By clicking on "Prediction" (1) at the top menu, users are directed to the job submission page (B). There users have the options to either provide a set of molecules as a SMILES file (2) or individual molecules as a SMILES string (3). Users can select the type of prediction (4). After selecting the type of prediction, and once calculations are complete, users are redirected to a results page (C) where predictions for GPCRs bioactivity are presented (5). Users have the option to download the results (6).

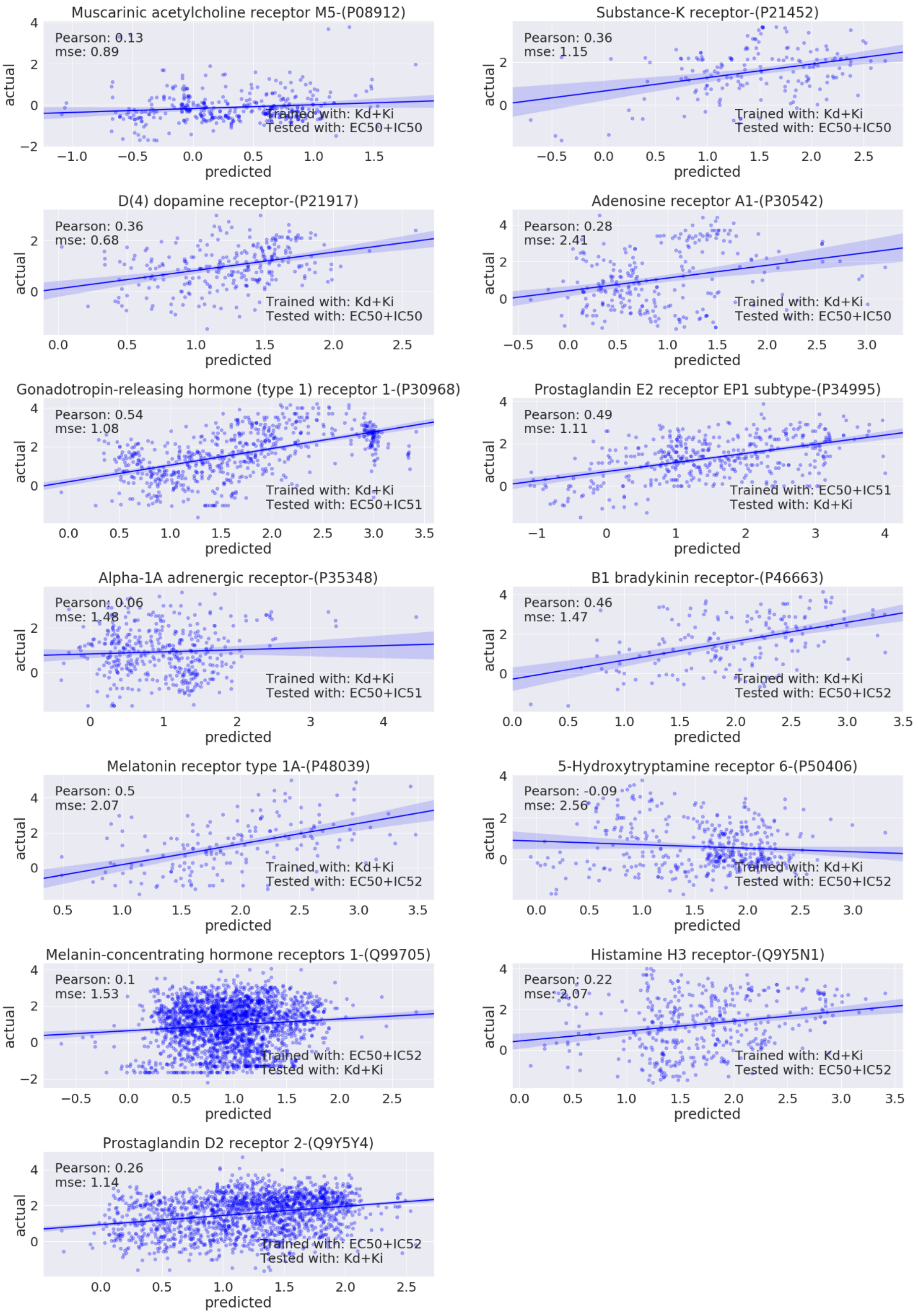

Figure S37: Scatter plots - Regression analysis for training with a bioactivity and testing with another. Pearson's correlation coefficients and MSE are also shown in the top-left corner. The graphs show the correlation between experimental and predicted values.

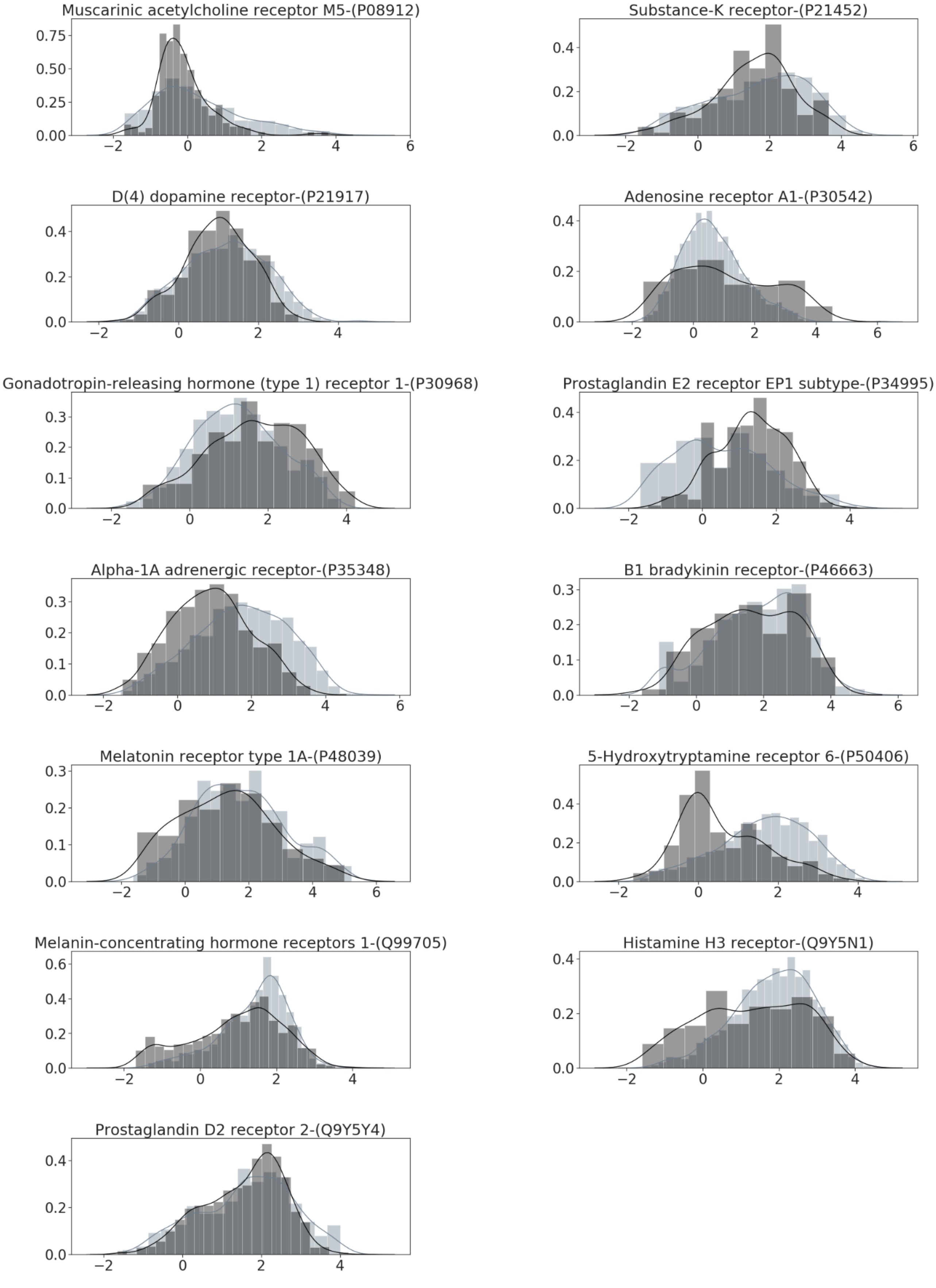

Figure S38: Histograms considering molecular activity distribution for training with a bioactivity and testing with another. The histogram in light grey colour represents training and the histogram in dark grey colour represents testing datasets.

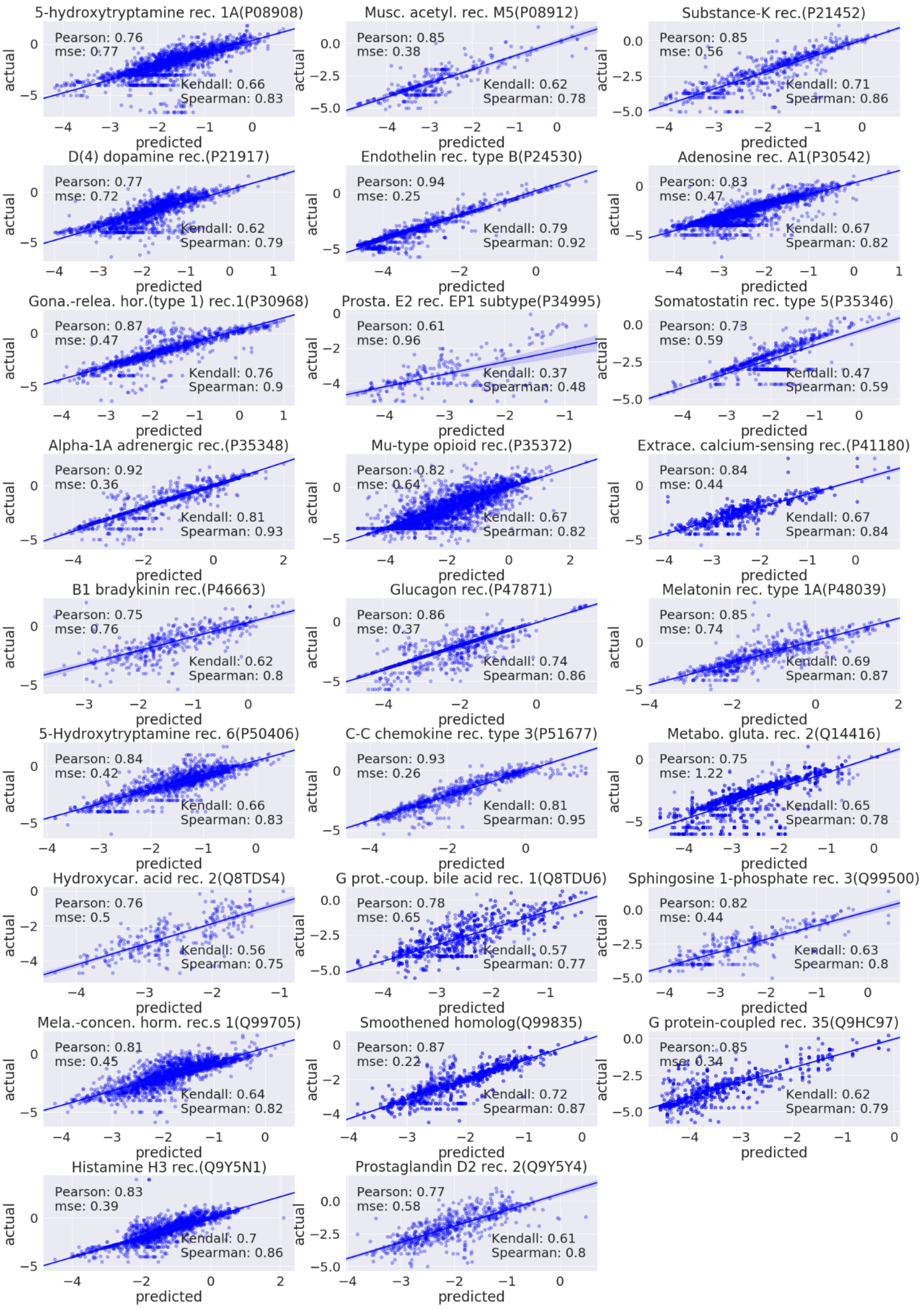

Figure S39: Scatter plots - Regression analysis for pdCSM-GPCR when testing with WDL-RF datasets. Pearson's correlation coefficients and MSE are also shown in the top-left corner. The graphs show the correlation between experimental and predicted values.

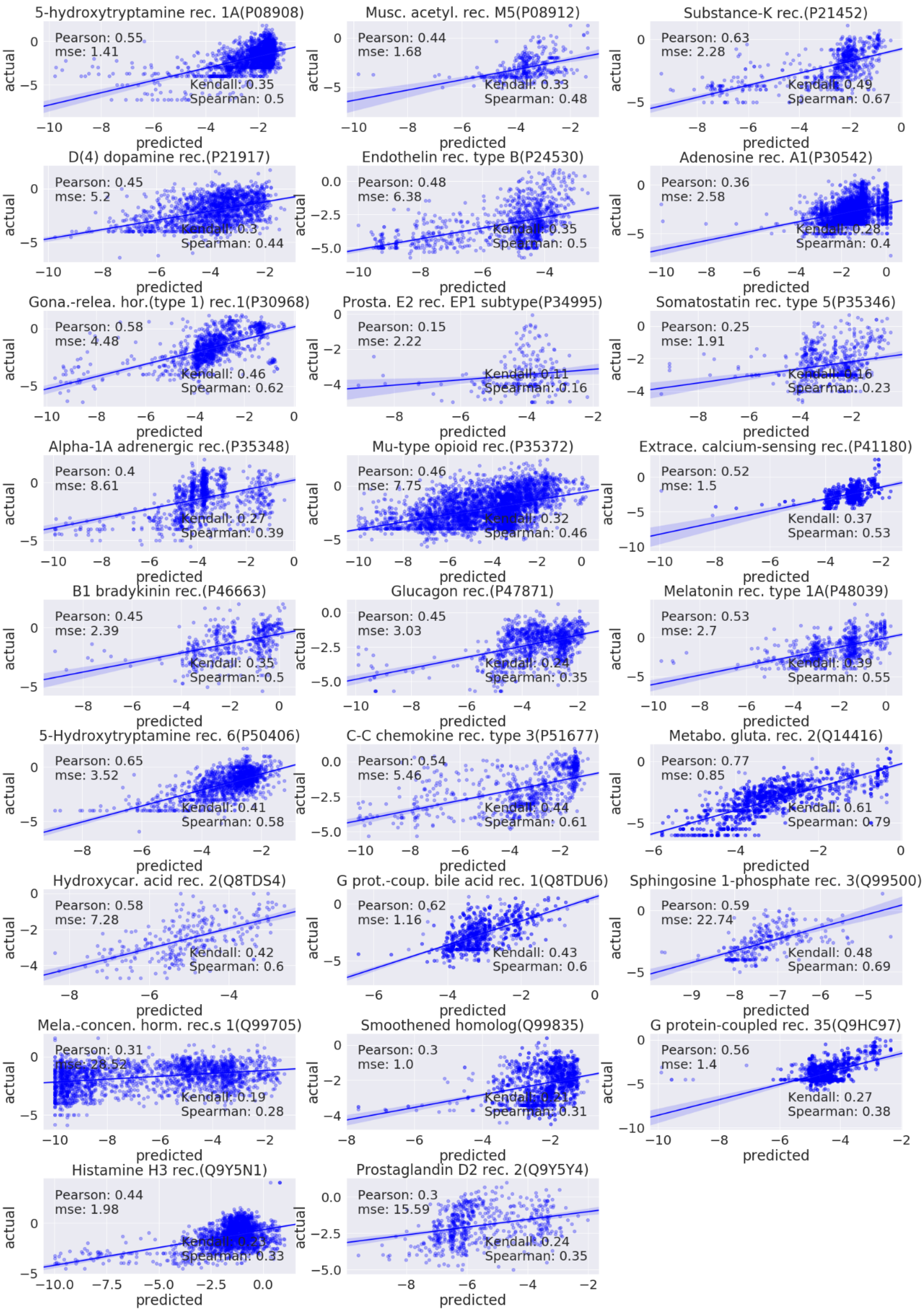

Figure S40: Scatter plots - Regression analysis for WDL-RF when testing with WDL-RF datasets. Pearson's correlation coefficients and MSE are also shown in the top-left corner. The graphs show the correlation between experimental and predicted values.

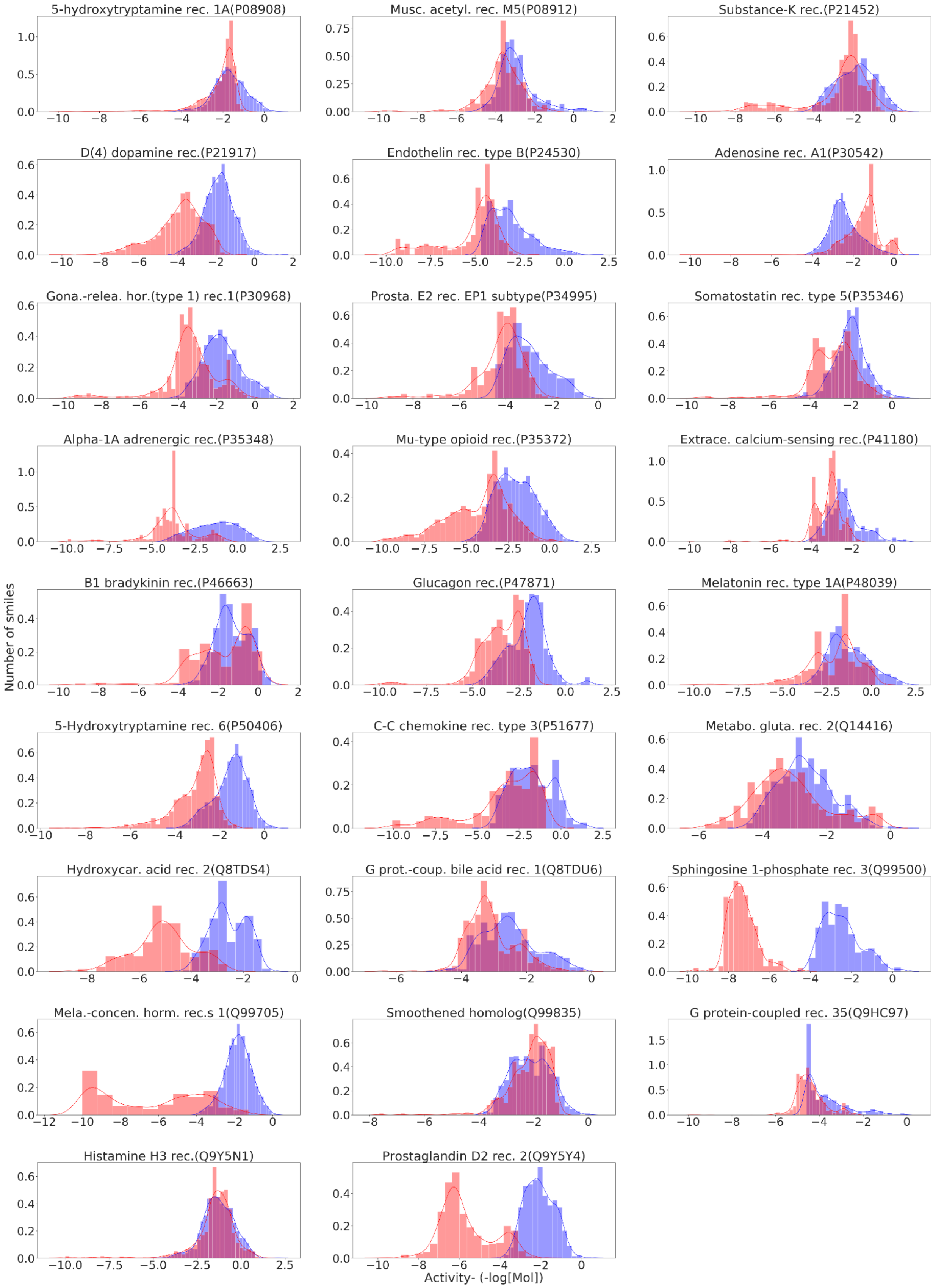

Figure S41: Histogram - comparing the activity outputs generated by the two servers, WDL-RF (red), pdCSM-GPCR (blue).

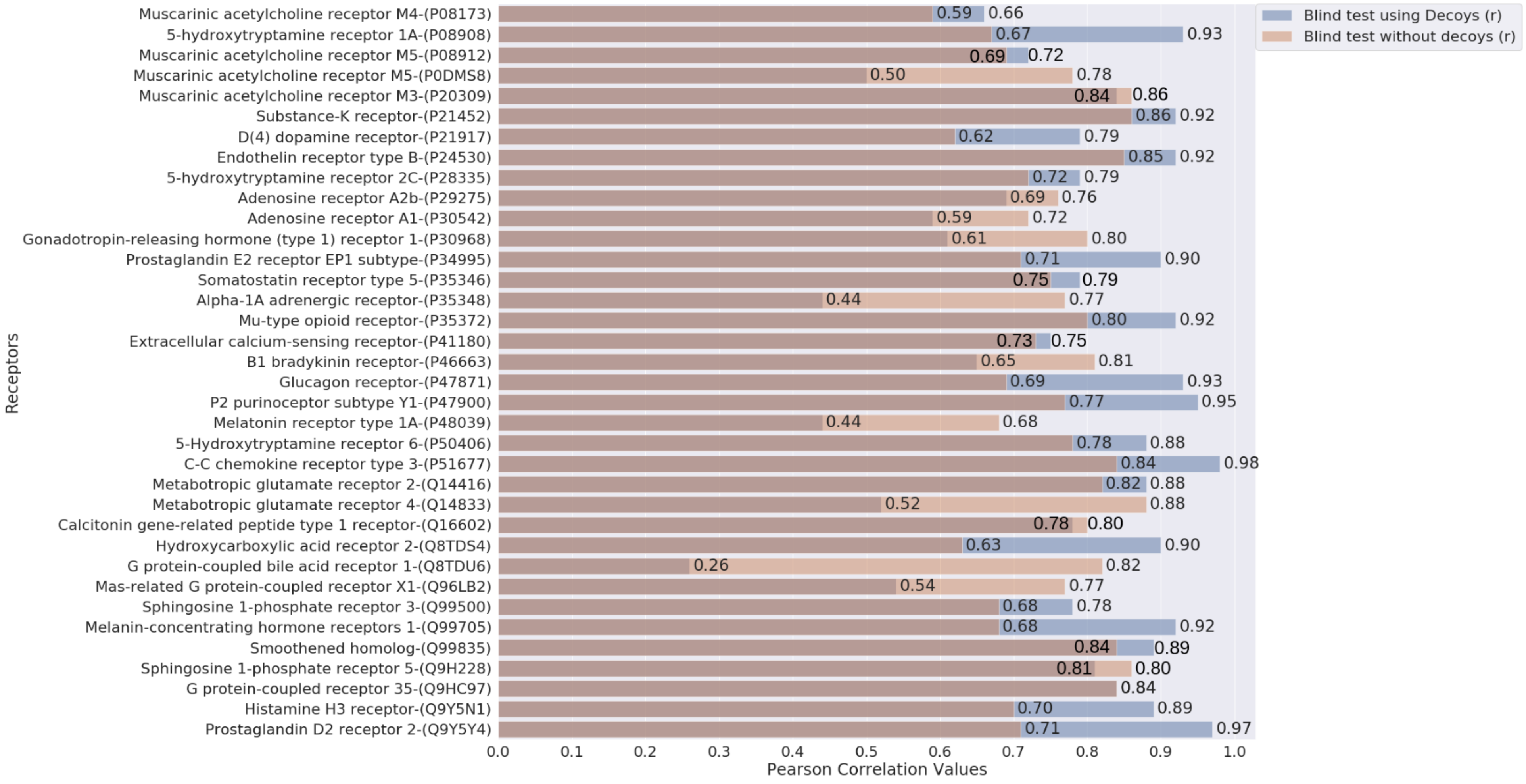

Figure S42: Performance comparison between pdCSM-GPCR with and without decoys.
